# Next-generation functional diagnostic paradigm for musculoskeletal diseases based on live SWIR imaging

**DOI:** 10.1038/s41467-026-77137-4

**Published:** 2026-09-14

**Authors:** Huizhu Li, Sijia Feng, Juan Liyau, Huaixuan Sheng, Shunyao Li, Xiao Zhang, Mo Chen, Liman Sai, Lu Fang, Chengxuan Yu, Kai Xu, Yan Wo, You Liang, Shiyi Chen, Yueming Wang, Fuchun Chen, Yunxia Li, Jun Chen

**Affiliations:** 1 Department of Sports Medicine, Huashan Hospital, Fudan University, Shanghai, China; 2 Department of Mathematics, Toronto Metropolitan University, Toronto, ON Canada; 3 Department of Joint Replacement, Sports Medicine, and Trauma,Department of Orthopedics, Renji Hospital, Shanghai Jiaotong University School of Medicine, Shanghai, China; 4 Department of Physics, Shanghai Normal University, Shanghai, China; 5 Shanghai Institute of Technical Physics (SITP) of the Chinese Academy of Sciences (CAS), Shanghai, China; 6 Department of Anatomy and Physiology, Shanghai Jiao Tong University School of Medicine, Shanghai, China; 7 Institutes of Integrative Medicine, Fudan University, Shanghai, China

**Keywords:** Fluorescence imaging, Translational research, Optical imaging, Bone, Ligaments

## Abstract

The pursuit of accurate diagnosis for musculoskeletal diseases has driven significant advancements in imaging technologies. However, fully understanding the complexity of the diseases requires monitoring multiple functional components, particularly during locomotion. Here, we present a live short-wave infrared (SWIR) imaging strategy designed to capture functional variations of musculoskeletal diseases in male murine models, and further extend it to male rabbit models and human knee joint. This approach directly visualized the kinematics of synovial fluid during normal locomotion, identifying distinct peak of fluid flow. When applied to injury evaluation, it revealed significant kinematic differences in patellar maltracking between isolated tendon injuries and combined tendon and muscle injuries, providing mechanical insights into their long-term outcomes. The versatility of this technique was further demonstrated by simultaneously examining bone and vascular kinematics during bone healing. This enabled the monitoring of large arteries in bone, and revealed an initial divergence from the bone regeneration. Overall, this advanced live SWIR imaging methodology enables dynamic, motion-robust, and multi-componential assessments of functional variations, positioning it as a next-generation functional diagnostic tool for musculoskeletal diseases.

## Abstract (web summary)

Here, the authors develop a live SWIR imaging strategy to enable dynamic, multi-component functional diagnosis of musculoskeletal diseases. It visualizes synovial fluid flow during locomotion, reveals patellar maltracking, and shows bone and vascular kinematics during healing.

## Introduction

The diagnosis of musculoskeletal diseases has substantially relied on the revolution of imaging technologies. X-ray, computed tomography (CT), ultrasound (US), and magnetic resonance imaging (MRI) have been widely employed over the century, enabling the identification of anatomic structures^1–3^. Today, imaging technologies have increasingly focused on the functional assessment of musculoskeletal diseases, including locomotion^4,5^ and vascular microenvironment^6^. This emphasis arises from the understanding that abnormal motion kinematics exclusively occurs in locomotion, as well as interruption of blood supply could produce a variety of musculoskeletal diseases such as knee osteoarthritis, patellofemoral syndrome and avascular necrosis of bones, etc.^7–11^. Therefore, the development of imaging technology incorporating functional assessment is anticipated to contribute to a holistic diagnosis of musculoskeletal diseases.

The imaging technologies for functional assessment have experienced technical advances in the last few decades. For example, gait analysis^12^, surface-tracking^13^, and dual fluoroscopy^14^ were developed and enabled the assessment of motion kinematics. Though motion-robust, they fall short of delineating precise anatomic structures and vasculature due to intrinsically limited technical features such as spatial resolution. In contrast, dynamic MRI^4^, MRI arthrography^15^, CT and MRI angiography^16^, as well as experimental in vivo multi-photon imaging^17,18^ approaches achieved multi-componential assessments of musculoskeletal diseases along with vasculature. Unfortunately, these approaches require the limb being imaged to be immobilized or relatively static to prevent deterioration of the spatial and temporal resolution. As a result, holistic assessment of musculoskeletal functions has remained challenging due to the lack of imaging technology enabling both motion-robust and multi-componential assessment.

Recently, short-wave infrared (SWIR, 1000–1700 nm) imaging is opening up a door to explore musculoskeletal diseases from a novel, dynamic perspective. As an optical imaging, substantially enhanced temporal resolution (up to ~100 frames per second) enabled real-time functional assessments of dynamic physiological events including blood perfusion, lymphatic pump activity, etc^19–21^. Besides, it has shown unparalleled contrast and resolution for in vivo visualization of deep-seated anatomic structures, including joints and bones^22–27^, as well as vasculature^28–32^. Consequently, SWIR imaging has emerged as a competitive technique for visualizing multiple functional components with high temporal resolution, yet its potential in the functional diagnosis of musculoskeletal diseases, which involves moving macro-objects, remains uncharted territory.

In this study, in vivo, real-time, long-term SWIR imaging (live SWIR imaging) was designed for the next-generation assessment of musculoskeletal functions during locomotion. Firstly, deep key anatomic structures of the knee joint and their motion kinematics were assessed during motion in normal rat models. Then, the real-time alterations of both anatomic structures and motion kinematics were investigated in tendon injury and combined tendon and muscle injury models. The imaging system was further extended to rabbit models and the human knee joint specimen. Subsequently, another functional component, longitudinal spatiotemporal progression of vascular remodeling and bone healing, was monitored in the mouse tibial defect model. Finally, all the above-mentioned vessels, bones and joints were sequentially assessed in the same individual animal model. The proposed live SWIR imaging enabled motion-robust and multi-componential assessments, providing an approach for the next-generation functional diagnosis of musculoskeletal diseases.

## Results

### Identification of deep anatomic structures of musculoskeletal system

**Fig. 1:**
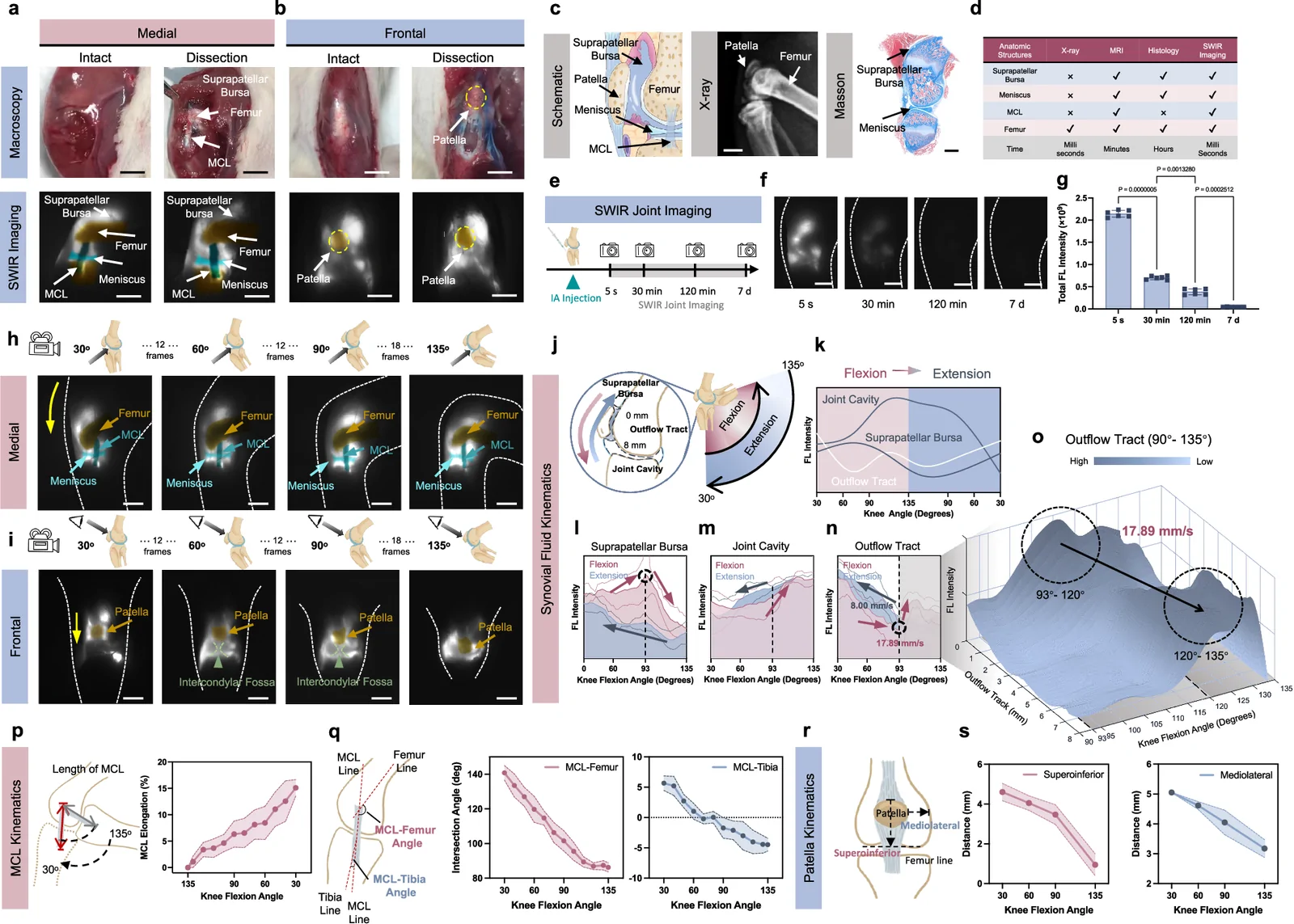
In vivo real-time visualization of deep anatomic structures and motion kinematics during the entire knee flexion. **a**, **b** Bright-field (Injection: methylene blue) and SWIR (Injection: SWIR fluorophores) images of the rat knee from the medial (**a**) and frontal (**b**) view, demonstrating deep anatomic structures of the knee joint without dissection based on SWIR imaging. Scale bar: 0.5 cm. **c** Schematic, X-ray, and Masson’s trichrome staining of the knee joint, demonstrating available anatomic structures. Scale bar: 0.5 cm (X-ray), 250 μm (Masson's trichrome staining). **d** Comparison table of X-ray, MRI, Masson’s trichrome staining, and SWIR imaging showing available anatomic structures and required time period. **e** A schematic of longitudinal SWIR joint imaging for clearance investigation of SWIR fluorophores. IA: Intra-articular. **f** Clearance of SWIR fluorophores at 5 s, 30 min, 120 min, 7 d post-injection. Scale bar: 0.5 cm. **g** Total FL intensity measured from (**f**) showing a gradual decrease in 7 d after injection. **h** Representative SWIR images of the knee joint during flexion from the medial view. Flexion angle of 30°, 60°, 90°, 135° were selected for demonstration. Dark yellow: Femur, Teal blue: MCL, Mint: Meniscus. Yellow arrow: Direction of synovial fluid movement. Scale bar, 0.5 cm. **i** Representative SWIR images of the knee joint during flexion from the frontal view. Flexion angle of 30°, 60°, 90°, 135° were selected for demonstration. Dark yellow: Patella, Green arrow: Intercondylar fossa. Yellow arrow: Direction of synovial fluid movement. Scale bar: 0.5 cm. **j** Schematic of suprapatellar bursa, outflow tract, and joint cavity in the knee joint, as well as knee flexion and extension process. Pink arrow: Direction of fluid flow in knee flexion. Blue arrow: Direction of fluid flow in extension. **k** Synovial fluid kinematics circulating between the suprapatellar bursa and joint cavity during knee flexion and extension. **l**–**n** Comparative analysis of synovial fluid kinematics in the suprapatellar bursa (**l**), joint cavity (**m**), and outflow tract (**n**) during knee flexion versus extension, revealing sharp flow (pink arrow) during flexion and mild flow (blue arrow) during extension. **o** A 3D graph showing synovial fluid kinematics in the outflow tract during knee flexion (90°–135°) based on the coordinates defined in schematic (**j**), revealing detailed high-velocity movement of synovial fluid flow. **p** Schematic and quantitative analysis of MCL elongation during knee extension. **q** Schematic and quantitative analysis of the intersection angle of MCL-Femur and MCL-Tibia during knee flexion, showing a larger range of motion in MCL-Femur than MCL-Tibia. **r** Schematics of patella kinematics demonstrating two directions of patellar displacements. **s** Superoinferior and mediolateral patellar displacement with knee flexion, demonstrating inferior and medial patella movement during knee flexion. Data were presented as the Mean ± SD of six independent rats (*n* = 6) and three independent rats for synovial fluid analysis (*n* = 3). Source data are provided as a Source Data file. F Femur, T Tibia, MCL Medial collateral ligament. Temporal resolution: 30 ms. P values were calculated by repeated measures ANOVA with Tukey’s test for multiple comparisons. Icons in **e**, **h**, **i** and **j** were created in BioRender. Li, H. (2026) https://BioRender.com/oye0g8y.

Identifying deep anatomic structures with high temporal resolution is an essential prerequisite for the functional assessment of the musculoskeletal system. Firstly, to identify the deep anatomic structures, macroscopy and SWIR joint imaging were conducted in rat models from medial and frontal views (Fig. 1a, b). Macroscopic examination (Fig. 1a, b) revealed that deep anatomic structures, including the suprapatellar bursa, femur, medial collateral ligament (MCL), and patella, were identified exclusively following dissection of surrounding tissues. In comparison, SWIR joint imaging of intact knee joints demonstrated these deep anatomic structures without dissection after intra-articular injection of SWIR fluorophores, indicating SWIR joint imaging possesses superiority in imaging depth. These deep anatomic structures were further verified with layer-by-layer dissection (Supplementary Fig. 1a–c), suggesting SWIR joint imaging enabled robust identification of the major anatomical structures within the knee with minimal invasion.

Subsequently, the temporal resolution and available anatomic structures were compared between SWIR joint imaging and traditional imaging modalities, including X-ray, MRI, and histological microscopy (Fig. 1c, d). Referring to the schematics, the X-ray provided a clear visualization of the bones in milliseconds (Fig. 1c, d). Meanwhile, the MRI and Masson’s trichrome staining required multiple slices and several minutes or hours for thorough observation of bone, meniscus, and ligaments of the knee joint (Fig. 1c, d and Supplementary Fig. 2a–c). In comparison, SWIR joint imaging enabled the visualization of key anatomic structures in Fig. 1d with a single snapshot in milliseconds. Therefore, it could provide a high temporal resolution and single-step approach for comprehensive observation of the anatomic structures of knee joints compared to traditional imaging modalities.

Moreover, given the critical importance of contrast agent clearance for effective biomedical translation of imaging modalities, the in vivo clearance profile of SWIR fluorophores for joint imaging was investigated (Fig. 1e–g). The SWIR fluorophores showed high fluorescence (FL) intensity (Supplementary Fig. 3a, b) and stability (Supplementary Fig. 3c–j) with ex vivo analyses. As illustrated in Fig. 1e, in vivo analyses (Fig. 1f) demonstrated that bright FL signals could be found immediately at 5 s post-injection, diminished quickly at 30 min post-injection, and finally disappeared completely at 7 d post-injection, which was confirmed by the corresponding quantitative analysis (Fig. 1g and Supplementary Fig. 4a–c). In addition, ex vivo observation of completely dissected structures demonstrated the absence of remaining signals (Supplementary Fig. 5a, b). These results suggested that SWIR joint imaging should be conducted within 30 min to ensure optimal visualization and that the SWIR fluorophores could be cleared out from the joint cavity within 7 d, indicating the possibility of short intervals for repetitive imaging.

In summary, in vivo SWIR joint imaging enabled comprehensive visualization of deep anatomic structures with high temporal resolution, followed by rapid clearance of the contrast agent, thereby laying the foundation for subsequent in-depth and motion-robust functional assessment of the musculoskeletal system.

### In vivo real-time visualization of motion kinematics of normal musculoskeletal system

As the musculoskeletal system performs the vital function of locomotion, the analysis of motion kinematics serves as a critical component for functional assessment. Normal motion kinematics were quantitatively analyzed across representative anatomic structures, including synovial fluid, ligaments, and bones (Fig. 1h–s). Firstly, the real-time kinematics of synovial fluid were monitored with SWIR joint imaging during knee flexion (Supplementary Movies 1 and 2). As an essential component of the knee joint^33,34^, the kinematics of synovial fluid have been widely studied by ex vivo computational simulation, but hardly during in vivo locomotion. By dispersing into the synovial fluid, SWIR fluorophores effectively illuminated the fluid, rendering the synovial fluid dynamics visible through SWIR joint imaging. As shown in Fig. 1h, i, synovial fluid flowed into the joint cavity with increasing knee flexion angle. Meanwhile, MCL, meniscus, and patella could be visualized continuously and showed the highest signal-to-background ratio (SBR) in certain flexion angles (Supplementary Fig. 6a–c) due to the synovial fluid flow.

Subsequently, the kinematics of synovial fluid were precisely quantified in Fig. 1j–o through subregion segmentation of image sequences recording knee flexion and extension. Referring to the schematic illustration demonstrating three subregions (Fig. 1j), Fig. 1k showed decreased FL intensity in the suprapatellar bursa and concurrent increment in joint cavity during flexion, with a phase-inverted pattern observed during extension. This reciprocating pattern revealed the flow of synovial fluid from the suprapatellar bursa into the joint cavity during knee flexion, followed by recirculation during extension^35^. Intriguingly, the synovial fluid kinematic differed between knee flexion and extension (Supplementary Movie 1), which was quantified in three subregions of suprapatellar bursa (Fig. 1l), joint cavity (Fig. 1m) and outflow tract (Fig. 1n). As shown in Fig. 1l, during knee flexion, synovial fluid in suprapatellar bursa accumulated between 60°– 93° and flowed out rapidly between 93°–135° (pink arrow), while it circulated back steadily (blue arrow) during knee extension. Similarly, a steeper increment during knee flexion than extension was observed in the joint cavity (Fig. 1m). Specifically, the velocity of synovial fluid could be calculated from the outflow tract connecting suprapatellar bursa and joint cavity, owing to the clear flow of synovial fluid (Supplementary Movie 1 and Fig. 1n). The synovial fluid exhibited phased acceleration at 93°–135° of flexion with peak velocity of 17.89 ± 1.42 mm/s, in contrast to sustained and uniform flow throughout extension with a velocity of 8.00 ± 1.37 mm/s (Fig. 1n). The observed synovial fluid high-velocity movement during knee flexion might arise from the abrupt decompression of outflow tract, which initiated when the intra-suprapatellar bursa pressure exactly surpassed quadriceps femoris-induced patellar compression force that maintain outflow tract closure at the early phase of knee flexion^35^. To further elucidate the high-velocity movement of synovial fluid, a pixel-wise analysis was conducted along the outflow tract (Fig. 1o) referring to the coordinates depicted in schematic Fig. 1j. This detailed analysis revealed that the synovial fluid traversed the outflow tract between 93° – 120° of knee flexion, flowed into joint cavity between 120° – 135° of flexion, suggesting the high-velocity egress of synovial fluid into the load-bearing joint cavity during terminal flexion phase (120°–135°of flexion).

Additionally, given that the MCL is the most commonly injured knee ligament^36^, length modulation and spatial displacement of the MCL during knee movements were investigated based on in vivo SWIR imaging. Analysis in Fig. 1p presented progressive MCL elongation during knee extension (from 135° to 30°), exhibiting a length increase of 15.11 ± 1.42% beyond its length at full flexion (135°), and pathological length deviation could indicate the impaired ligament function^14,37,38^. For spatial displacements, as shown in Fig. 1q, the MCL underwent > 45° angular deviation to the femur during flexion, whereas it maintained near-parallel alignment to the tibia throughout knee flexion (<15° angular deviation). Notably, MCL motion kinematics measured via SWIR imaging showed negligible variation when validated against macroscopic measurements (Supplementary Fig. 7a–d). These results suggested that the MCL had a smaller range of motion at the tibial than the femoral insertion, and such image-based measurements in preclinical models could provide insights into MCL injuries in humans.

Furthermore, as an indispensable fulcrum bone in knee biomechanics, the patella and its tracking have been extensively associated with joint stability and disorders such as patellofemoral syndrome^39,40^. The diagnostic approach relying on MRI at a set flexion angle^40^ fails to capture the complete patellar tracking during natural joint motion and potentially leads to diagnostic inaccuracies, highlighting the need for real-time imaging techniques. Leveraging its real-time imaging capability, SWIR joint imaging demonstrated whole patellar tracking of inferior and medial displacement during knee flexion (Fig. 1i, illustrated in Fig. 1r) in living subjects. Quantitative analysis (Fig. 1s) revealed greater superoinferior (3.66 ± 0.41 mm) displacement compared to mediolateral displacement (1.88 ± 0.25 mm), which aligned with a previous cadaveric study^39^. Consistent with MCL validation, SWIR-derived patellar displacement data showed strong agreement with direct macroscopic measurements (Supplementary Fig. 8a–d). Therefore, SWIR joint imaging enabled the in vivo visualization of complete patellar tracking, thus providing a precise and visually intuitive method for motion kinematic analysis.

Taken together, SWIR joint imaging integrates in vivo real-time visualization of anatomic structures with kinematical profiling of synovial fluid, ligament, and bone, thus enabling a holistic, motion-robust functional assessment of the musculoskeletal system during locomotion.

### Functional diagnosis of musculoskeletal diseases by incorporating anatomic structures and motion kinematics based on SWIR joint imaging

**Fig. 2:**
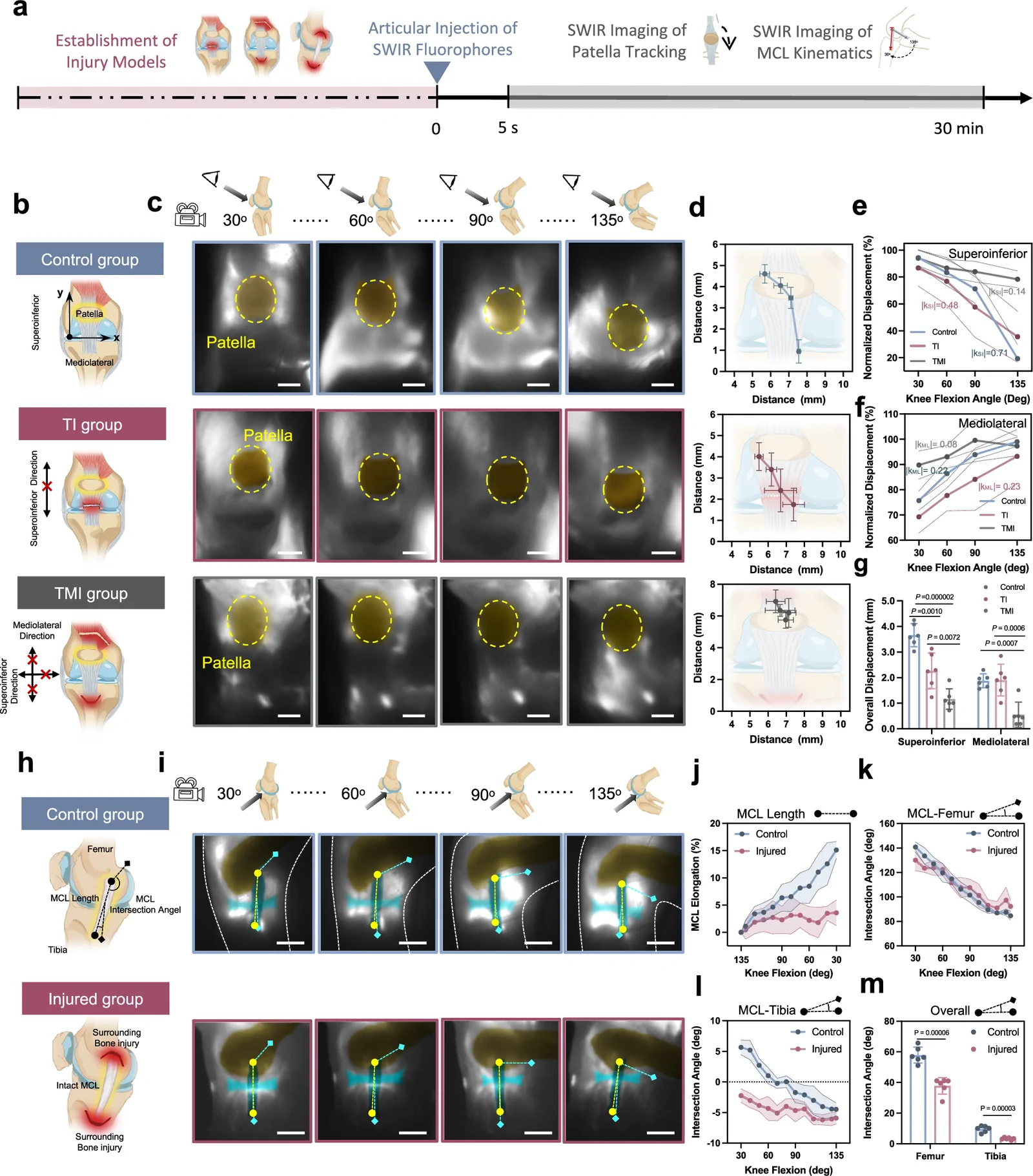
Functional diagnosis of musculoskeletal diseases via motion kinematics assessment. **a** A schematic of SWIR joint imaging of injury models. **b** Schematics of control, and two types of patellar tendon-related injury models. Tendon injury (TI): Injury in superoinferior direction. Combined tendon and muscle injuries (TMI): Injuries in both superoinferior and mediolateral direction. **c** Representative SWIR images of the control, TI, and TMI groups form the frontal view during knee flexion with the angles of 30°, 60°, 90°, and 135°, demonstrating varying patterns of patellar displacement. Scale bar: 0.25 cm. **d** Patellar trackings of control, TI, and TMI groups quantified from (**c**). **e** Comparison of superoinferior displacement of patella in control, TI, and TMI group, as well as corresponding slopes of the patellar tracking (|k_SI_|), displaying gradually decreasing slopes in control, TI, and TMI group. **f** Comparison of mediolateral displacement of patella and corresponding slopes (|k_ML_|), displaying a smaller slope in the TMI group than the control and TI group. **g** Comparison of overall patellar displacement in superoinferior and mediolateral direction, demonstrating impaired superoinferior displacement in the TI group, and impaired both directional displacements in the TMI group. **h** Schematics of control, bone injury model with intact MCL, and corresponding MCL kinematical assessment. **i** Representative SWIR images of the MCL in control and injured groups during knee flexion from the medial view. Flexion angle of 30°, 60°, 90°, 135° were selected for demonstration. Yellow line: MCL line. Cyan line: Extended lines of the femur and tibia. Scale bar: 0.25 cm. **j** Comparison of MCL length modulation during knee flexion between groups, showing impaired MCL elongation in the injured group. **k**, **l** Comparison of MCL-Femur (**k**) and MCL-Tibia (**l**) intersection angle during knee flexion, demonstrating impaired angle variations in the injured group. **m** Comparison of overall MCL spatial displacement during knee flexion, demonstrating impaired spatial displacement in the injured group. Data were presented as the Mean ± SD of six independent rats (*n* = 6). Source data are provided as a Source Data file. P values were calculated by one-way ANOVA with Tukey’s test for multiple comparisons. Two-tailed unpaired t-tests were used for two-group comparisons. Icons in **a**–**d**, **h** and **i** were created in BioRender. Li, H. (2026) https://BioRender.com/dqavw51.

In musculoskeletal diseases, alterations in anatomic structures do not necessarily represent functional impairment, and vice versa. Therefore, comprehensive assessments incorporating anatomic structures and kinematics were assessed in tendon injury (TI) and combined tendon and muscle injuries (TMI) models (Fig. 2). Firstly, referring to schematic illustrations Fig. 2a, b, the knee flexion process of TI and TMI models was monitored (Supplementary Movies 3 and 4), and the alterations in anatomic structures were identified. During the entire knee flexion, the injuries could be identified continuously with the length consistent with that from macroscopy in TI (Supplementary Fig. 9a–e) and TMI models (Supplementary Fig. 10a–e), enabling the simple identification of anatomic alterations in musculoskeletal injuries during movements.

Subsequently, to investigate whether SWIR joint imaging could achieve in vivo kinematical assessment, patellar tracking was investigated in TI and TMI models (Fig. 2c–g). Previous results in Fig. 1r, s have shown that the patella from the control group showed two directions of displacement, including superoinferior and mediolateral. As demonstrated in Fig. 2c, the TI group maintained inferior and medial patellar displacement, whereas this displacement pattern became undetectable in the TMI group. It was confirmed by quantitative measurements (Fig. 2d), indicating that SWIR joint imaging could discern kinematic alterations. To further specify the degrees and directions of patellar malfunction, the slope of the patellar tracking (|k|) in superoinferior (|k_SI_|) and mediolateral (|k_ML_|) directions was compared between groups (Fig. 2e, f). For superoinferior direction (Fig. 2e), |k_SI_| decreased gradually in the control (|k_SI_| = 0.71), TI (|k_SI_| = 0.48), and the TMI group (|k_SI_| = 0.14), indicating the declining |k_SI_| sensitively corresponded to the severity of injuries and malfunction of the patella in the superoinferior direction. Meanwhile, for mediolateral direction (Fig. 2f), the TMI group (|k_ML_| = 0.08) showed a smaller |k_ML_| than that of TI and the control group (|k_ML_| = 0.23, 0.22), suggesting a smaller |k_ML_| specifically indicated the injury and malfunction of the patella in the mediolateral direction. The overall superoinferior and mediolateral displacement (Fig. 2g) also confirmed the above results. Therefore, SWIR joint imaging was capable of discerning different kinds of knee musculoskeletal injuries and malfunctions sensitively and specifically by incorporating kinematic alterations of the patella in two directions, which were not discernible with CT and MRI images (Supplementary Fig. 11a–h).

Moreover, to investigate the diagnostic potential of SWIR joint imaging for functional evaluation prior to anatomical alterations, functional assessment of MCL kinematics, including length modulation and spatial displacement, was performed in a bone injury model, which preserved ligament integrity (Fig. 2h). As shown in Fig. 2i, the impaired length modulation and abnormal MCL-Tibia intersection angles could be identified in the injured group. The quantitative analysis demonstrated that the MCL in the injured group showed impaired elongation capability (Fig. 2j), less MCL-Femur (Fig. 2k, m) and MCL-Tibia (Fig. 2l, m) intersection angle variations, though with intact anatomic structures of MCL, confirming that SWIR joint imaging could sensitively conduct functional assessment prior to anatomical alterations. In short, SWIR joint imaging not only enabled continuous in vivo real-time identification of anatomic alterations during motion but also discerned kinematic alterations sensitively and specifically, thereby providing a comprehensive and motion-robust functional diagnostic approach for musculoskeletal injuries.

### Extending SWIR joint imaging to rabbit models and the human knee joint

**Fig. 3:**
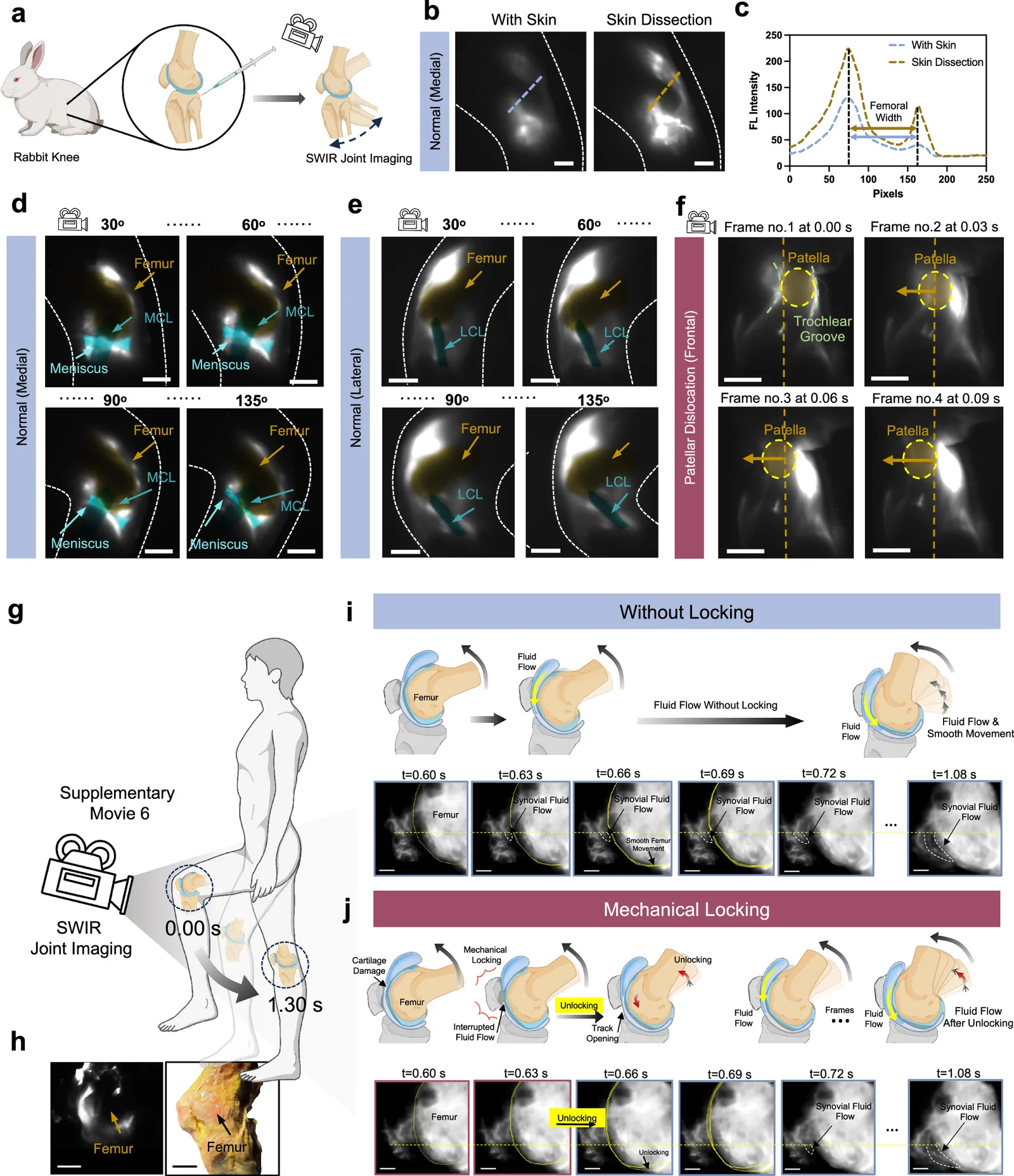
Anatomical and functional assessment of rabbit models and human knee joint based on SWIR joint imaging. **a** A schematic of the SWIR joint imaging process in the rabbit model. **b** SWIR images of the rabbit knee acquired from the medial view, comparing imaging with intact skin and post-dissection conditions. Scale bar: 1.0 cm. **c** Comparison of femoral width measurements between the SWIR images in panel (**b**), which confirms consistent measurement accuracy irrespective of skin dissection status. **d**, **e** Representative SWIR images of rabbit knee anatomic structures during flexion from the medial view (**d**) and the lateral view (**e**). Flexion angle of 30°, 60°, 90°, 135° were selected for demonstration. Scale bar: 1.0 cm. MCL, Medial collateral ligament; LCL, Lateral collateral ligament. **f** Patellar dislocation process of the rabbit knee visualized by SWIR joint imaging. Scale bar: 1.0 cm. **g** Schematic of SWIR joint imaging in human knee joint extension in the time period of 1.30 s. **h** SWIR and bright-field images of the human knee joint showing the anatomic structure of the femur. Scale bar: 2 cm. **i** Schematics and SWIR images of human knee movement and synovial fluid flow without locking. Scale bar: 0.5 cm. **j** Schematics and SWIR images of human knee movement with mechanical locking, subsequent unlocking and delayed synovial fluid flow. Scale bar: 0.5 cm. Six independent rabbits (*n* = 6) and one human cadaveric body (*n* = 1) were included in the imaging. Source data are provided as a Source Data file. Icons in **a**, **d**, **e**–**g**, **i** and **j** were created in BioRender. Li, H. (2026) https://BioRender.com/8oroeoa.

To demonstrate translational scalability, the SWIR joint imaging system was implemented in rabbit models and human knee joint specimen. In rabbit knees (Fig. 3a), the system non-invasively visualized the femur through the skin (Fig. 3b), achieving comparable width accuracy to that seen with skin dissection (Fig. 3c). Similar to findings in murine models, normal synovial fluid and anatomical structures were successfully visualized during knee flexion from both medial and lateral views (Fig. 3d, e and Supplementary Movie 5). Critically, the system could capture the abnormal kinematics of patellar dislocation in real-time (Fig. 3f and Supplementary Movie 5), from normal patellar location in the trochlear groove (frame no.1) to laterally dislocated patella out of the trochlear groove (frame no. 4) in the time period of 0.09 s.

To further scale up the system and explore its clinical translational potential, SWIR joint imaging was utilized to visualize human knee extension over a 1.30-second period (Fig. 3g) immediately after articular injection of probes and skin dissection, which identified anatomic structure of the femur (Fig. 3h). Interestingly, two distinct movement patterns were observed during knee extension: one characterized by smooth, uninterrupted motion (Fig. 3i) and the other involving mechanical locking and subsequent unlocking (Fig. 3j). As shown in Fig. 3i and Supplementary Movie 6, knee extension without locking displayed smooth femur movement, evidenced by uniform displacement between frames (SWIR images: yellow outline depicting the location of femur). Synovial fluid flow was detectable at 0.63 s (white dotted line) during this smooth movement. In contrast, knee extension with mechanical locking (Fig. 3j and Supplementary Movie 6) did not show the fluid flow at 0.63 s. Instead, a sudden unlocking event occurred between 0.63 s and 0.66 s, marked by a sharp increase in femur displacement between frames (SWIR images: yellow outline depicting the location of the femur). Subsequently, the delayed synovial fluid flow was observed after joint unlocking at 0.72 s (marked by a white dotted line). This mechanical locking may arise from femoral cartilage damage, which could impair lubricating film formation upon contact with the patella^41^, thereby disrupting smooth femoral movement during extension. These findings suggested the translational scalability of live SWIR imaging and further underscored the critical need for functional movement assessment within short timeframes, which is a capability challenging to achieve with existing technologies.

### Multi-componential SWIR vessel and bone imaging for longitudinal assessment of musculoskeletal injuries

**Fig. 4:**
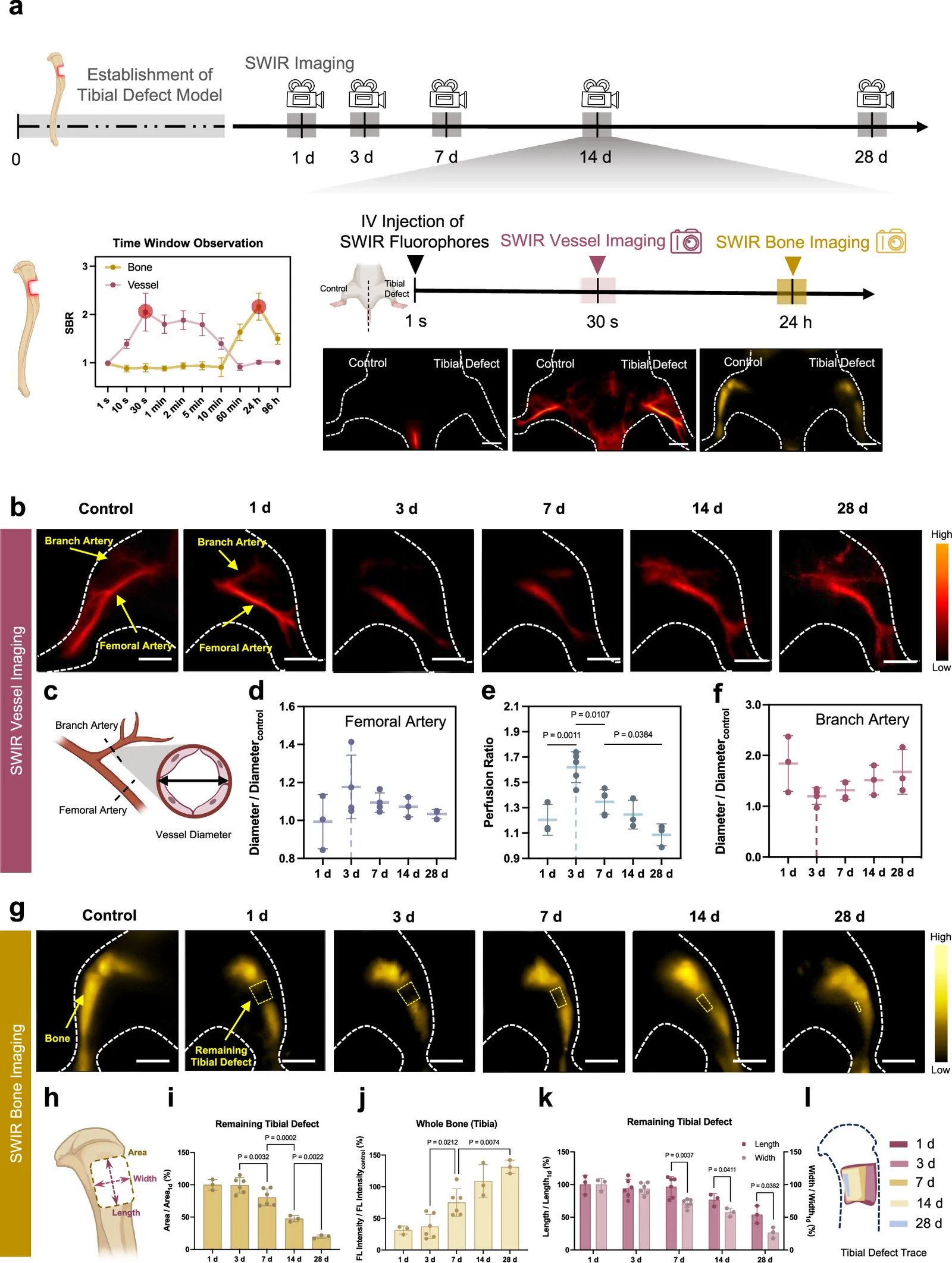
In vivo analysis of vasculature and bone in mouse tibial defect model based on SWIR vessel and bone imaging. **a** A schematic of the SWIR vessel and bone imaging of a mouse tibial defect model. The imaging time window was observed to be 30 s post-injection for the vessel and 24 h post-injection for the bone. **b** SWIR images of vessels, including femoral artery and branch artery at 1 d, 3 d, 7 d, 14 d, 28 d post-surgery with 30 s after tail vein injection of SWIR fluorophores. **c** Schematic of vessel diameter measurements. **d** Normalized diameter of femoral artery measured from (**b**), which showed a peak at 3 d post-surgery. **e** Prefusion ratio (injured/contralateral limb) in the tibial defect model measured from (**b**), demonstrating a peak at 3 d post-surgery. **f** Normalized diameter of branch artery measured from (**b**) showed a minimum value at 3 d post-surgery. **g** SWIR images of bone at 1 d, 3 d, 7 d, 14 d, 28 d post-surgery with tail vein injection of SWIR fluorophores at 24 h post-injection, with the shaded area (yellow box) indicating the tibial defect. **h** Schematic of measured length, width, and area of tibial defect. **i** A progressive reduction in the remaining area of the tibial defect measured from (**g**). **j** Increment in normalized FL intensity of the whole tibial defect area. **k** Normalized length and width of remaining tibial defect demonstrating more pronounced alterations in width than length from 7 d post-surgery. **l** A diagrammatic representation of the tibial defect regeneration process monitored by SWIR bone imaging, displaying progressive changes in width rather than length. Data were presented as the Mean ± SD of three to six independent mice for each time point. For 1 d, 14 d, 28 d post-surgery, three independent mice were included (*n* = 3). For 3 d, 7 d post-surgery, four, five and six independent mice were included for vessel diameter (*n* = 4), perfusion ratio (*n* = 5) and bone analysis (*n* = 6). Source data are provided as a Source Data file. Scale bar: 1 cm. P values were calculated by mixed-effects analysis for multiple comparisons and a two-tailed unpaired t-test for two-group comparisons. Icons in **a**, **c** and **h** were created in BioRender. Li, H. (2026) https://BioRender.com/l1bh6ee.

Recent studies highlighted the demand for a next-generation imaging approach for multi-componential monitoring of vasculature alongside the musculoskeletal system^3,42–45^. Therefore, we conducted multi-componential and spatiotemporal monitoring for vessels and bones (SWIR vessel imaging, SWIR bone imaging) in the tibial defect model based on live SWIR imaging. It could be achieved owing to the separate imaging time window of the vessel and the bone. As shown in Fig. 4a, the imaging time window of vessel and bone was 30 s–5 min and 60 min–96 h post-injection, respectively, enabling the sequential visualization of vessel and bone in a single mouse model. Specifically, the time points of 30 s and 24 h post-injection were selected for imaging based on their highest SBR (Fig. 4a and Supplementary Fig. 12a, b).

Based on SWIR vessel imaging, in vivo spatiotemporal variations in femoral and branch artery diameters were visualized from 1 d to 28 d post-surgery (Fig. 4b, c). The diameter of the femoral artery (Fig. 4d) and the corresponding perfusion ratio (Fig. 4e) peaked at 3 d post-surgery, suggesting the vascular dilatation for more blood supply^46^. In comparison, the diameter of the branch artery decreased 3 d post-surgery and showed a postponed rise (Fig. 4f), which could indicate vessel contraction for hemostasis before 3 d and subsequent dilation. The critical turning point was identified as 3 d post-surgery. Therefore, SWIR vessel imaging enabled the longitudinal monitoring of spatiotemporal changes in vessel diameter and the identification of the critical time point.

Along with the vessel, we also investigated the capability of SWIR bone imaging for precise monitoring of spatiotemporal patterns of bone healing. As shown in Fig. 4g, the shaded area (yellow box) exhibiting progressive reduction could be identified, which was confirmed with in situ analysis (Supplementary Fig. 13a–c). Moreover, the area, width, and length of the remaining tibial defect were quantified as illustrated in Fig. 4h. Quantitative validation revealed a reciprocal relationship between progressive reduction of remaining tibial defect area (Fig. 4i) and concomitant elevation of FL intensity in the whole bone (Fig. 4j) over the 28 days, indicating progressive bone regeneration, and the significance was firstly found between 3 d and 7 d post-surgery. Interestingly, as shown in Fig. 4k, the width of the remaining tibial defect demonstrated a more pronounced decrease than the length from 7 d post-surgery. This anisotropic healing pattern was schematically represented in Fig. 4l, revealing a spatiotemporal pattern of bone regeneration initiating from the bone surface toward the inner part, rather than longitudinal progression. It could be because the transcortical vessels, which are responsible for 50–70% of the total blood flow of the bone, firstly enter the bone surface and branch to inner regions, thus supplying more nutrients to the bone surface^47–49^. In summary, SWIR bone imaging enabled monitoring of precise spatiotemporal patterns in bone healing, with the 3–7 d postoperative period identified as a critical phase where statistically significant changes first emerged, paralleling the timeline of vascular changes.

**Fig. 5:**
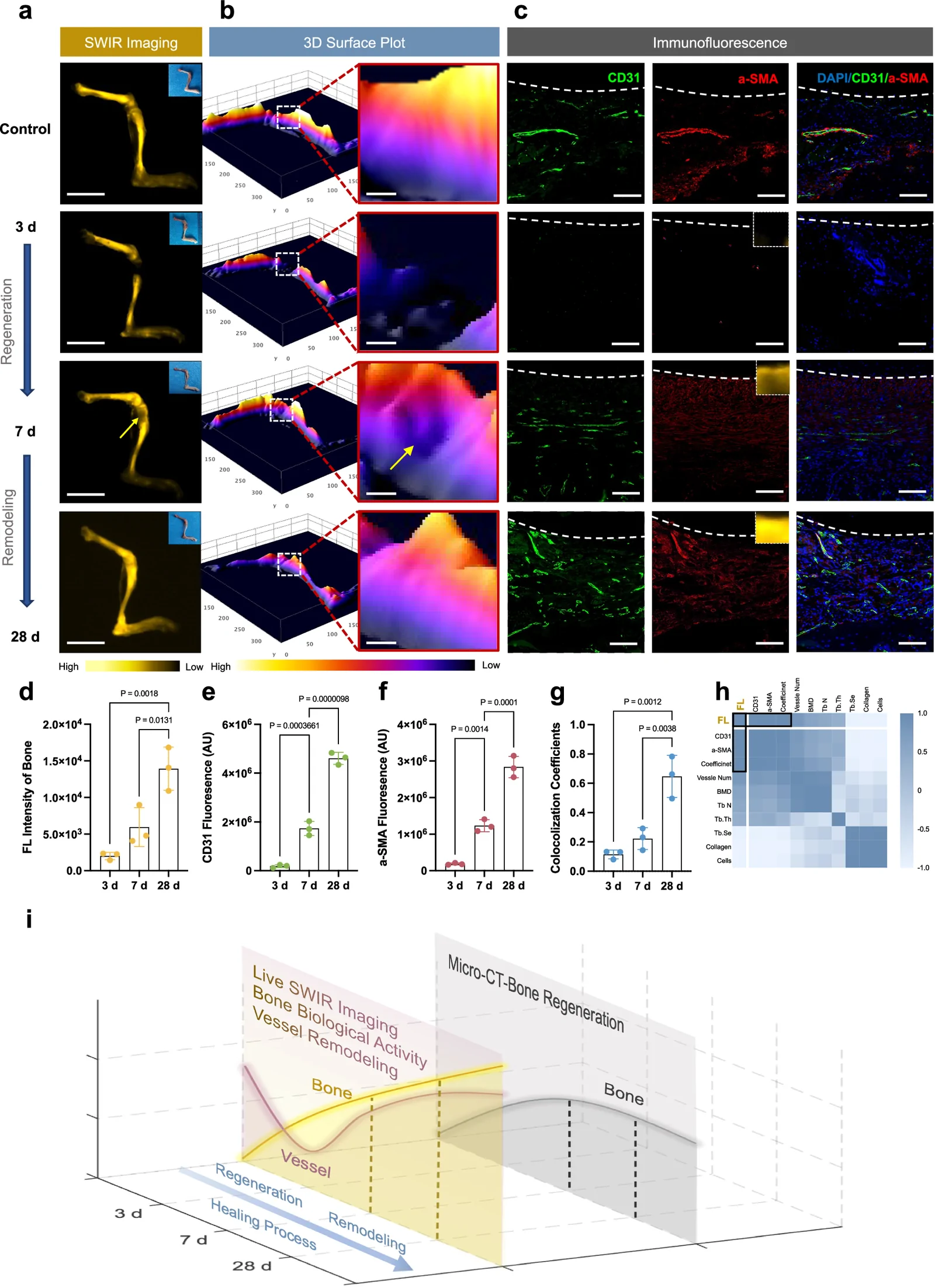
Ex vivo analysis of mouse tibial defect model. **a** Ex vivo SWIR images at 3 d, 7 d, 28 d post-surgery, showing progressive SWIR signal recovery in the tibial defect area. Yellow arrow: Inner part of the bone with low SWIR FL signals. Scale bar: 1 cm. **b** 3D surface plots of (**a**), showing details of the bone healing pattern of increasing SWIR signals in the bone surface rather than the inner part at 7 d. Yellow arrow: Inner part of the bone with low SWIR FL signals. Scale bar: 0.1 cm. **c** Immunofluorescence analyses of CD31 and α-SMA, as well as spatially correlated SWIR images (upper right panel of α-SMA). Scale bar: 100 um. **d** FL intensity of the whole tibia measured from (**a**), showing an increment over the time period. **e**–**g** CD31 fluorescence intensity (**e**), α-SMA fluorescence intensity (**f**), and colocalization coefficient of CD31 and α-SMA (**g**) of tibial defect, demonstrating an increasing pattern over the time period. **h** Correlation analysis among FL intensity from SWIR bone imaging and other parameters from traditional imaging modalities, including CD31, a-SMA, coefficient, vessel number, BMD, Tb. N, Tb. Th, Tb. Se, collagen, nucleated cells, displaying a strong correlation between bone FL intensity and CD31, a-SMA, and coefficient. **i** Graph summary of tibial defect progression monitored by live SWIR imaging as well as micro-CT, demonstrating multi-component and longitudinal monitoring ability of live SWIR imaging during the whole bone healing process. Data were presented as the Mean ± SD of three independent mice at each time point (*n* = 3). Source data are provided as a Source Data file. BMD Bone mineral density. Tb N Trabecular number, Tb Th Trabecular thickness, Tb Se Trabecular separation. P values were calculated by one- way ANOVA with Tukey’s test for multiple comparisons.

To verify the in vivo findings, further analyses were conducted at critical time points. Notably, at 7 d post-surgery, SWIR FL signals could be found at the bone surface (Fig. 5a), while substantially lower signals were found at the inner region (Fig. 5a, b, yellow arrow). However, the macroscopy (Supplementary Fig. 13a, b), micro-CT scans (Supplementary Fig. 14a–f), and histological analysis (Supplementary Fig. 15a–e) demonstrated regenerated bone trabecula and bone matrix in the inner regions, showing the inconsistency with SWIR FL signals of bone. This suggested that SWIR bone imaging not only indicated the regenerated bone tissue but also may provide further insights into the biological activity of bone during regeneration and subsequent remodeling.

To validate the correlation between SWIR bone imaging signals and biological activity of bone, the immunofluorescence analysis of CD31, α-SMA, as well as correlation analysis was conducted (Fig. 5c–h), since the vascularization acts as an indispensable biological activity during bone healing^6,43^.

Notably, at 7 d post-surgery (Fig. 5c), scattered CD31 and α-SMA signals were observed at the bone surface, which matched the spatial distribution of SWIR FL signals (SWIR images in the upper right panels), supporting the spatial correspondence between SWIR FL signals and the biological activity of vascularization. Moreover, the quantitative analysis (Fig. 5d–g) confirmed a gradual increase of CD31, α-SMA intensity, and colocalization coefficient between CD31 and α-SMA in pace with increasing SWIR FL intensity up to 28 d, indicating the temporal correspondence between SWIR FL signals and biological activity of vascularization. Finally, correlational validation revealed that SWIR FL intensity exhibited robust positive correlations with CD31(*r* = 1.000, *P* = 0.013), α-SMA intensity (*r* = 0.997, *P* = 0.046), while showing a non-significant association with other parameters (Fig. 5h). The results implied that the FL signals in bone may be related to the bone vessels^22,50^. Taken together, SWIR bone imaging revealed the spatiotemporal pattern of biological activity during the entire bone healing process, thereby underscoring its potential to assess the function of musculoskeletal diseases and guide the phase-based treatment in the future.

To summarize the advancement of SWIR imaging, Fig. 5i depicts the comparative trends of parameters measured from SWIR imaging and micro-CT. In comparison with micro-CT, demonstrating constant bone volume from 7 d to 28 d post-surgery, SWIR imaging demonstrated consistently increasing SWIR FL intensity of bone along with varying vessel diameter till 28 d post-surgery. Therefore, SWIR imaging offered a multi-componential approach for spatiotemporal monitoring of both vascular changes and biological activity of bone during the entire bone healing process.

### Holistic functional assessment of musculoskeletal disease based on sequential live SWIR imaging

**Fig. 6:**
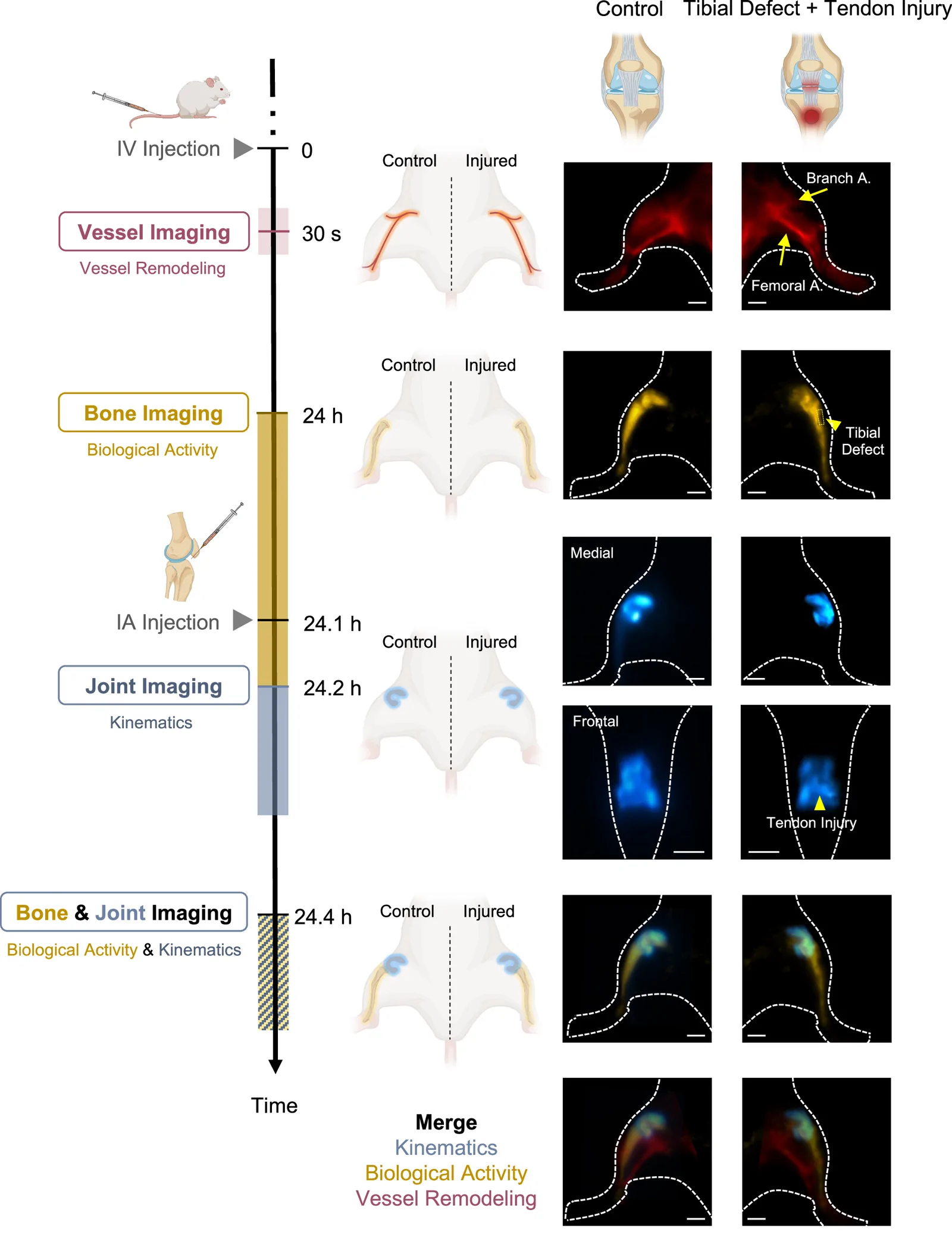
Holistic functional assessment of musculoskeletal disease based on sequential SWIR vessel, bone and joint imaging. Three independent mice were included in the experiment (*n* = 3). IV Intravenous; IA Intra-articular; Femoral A Femoral artery; Branch A Branch artery. Scale bar: 0.5 cm. Part of icons were created in BioRender. Li, H. (2026) https://BioRender.com/ge3acxb.

**Fig. 7:**
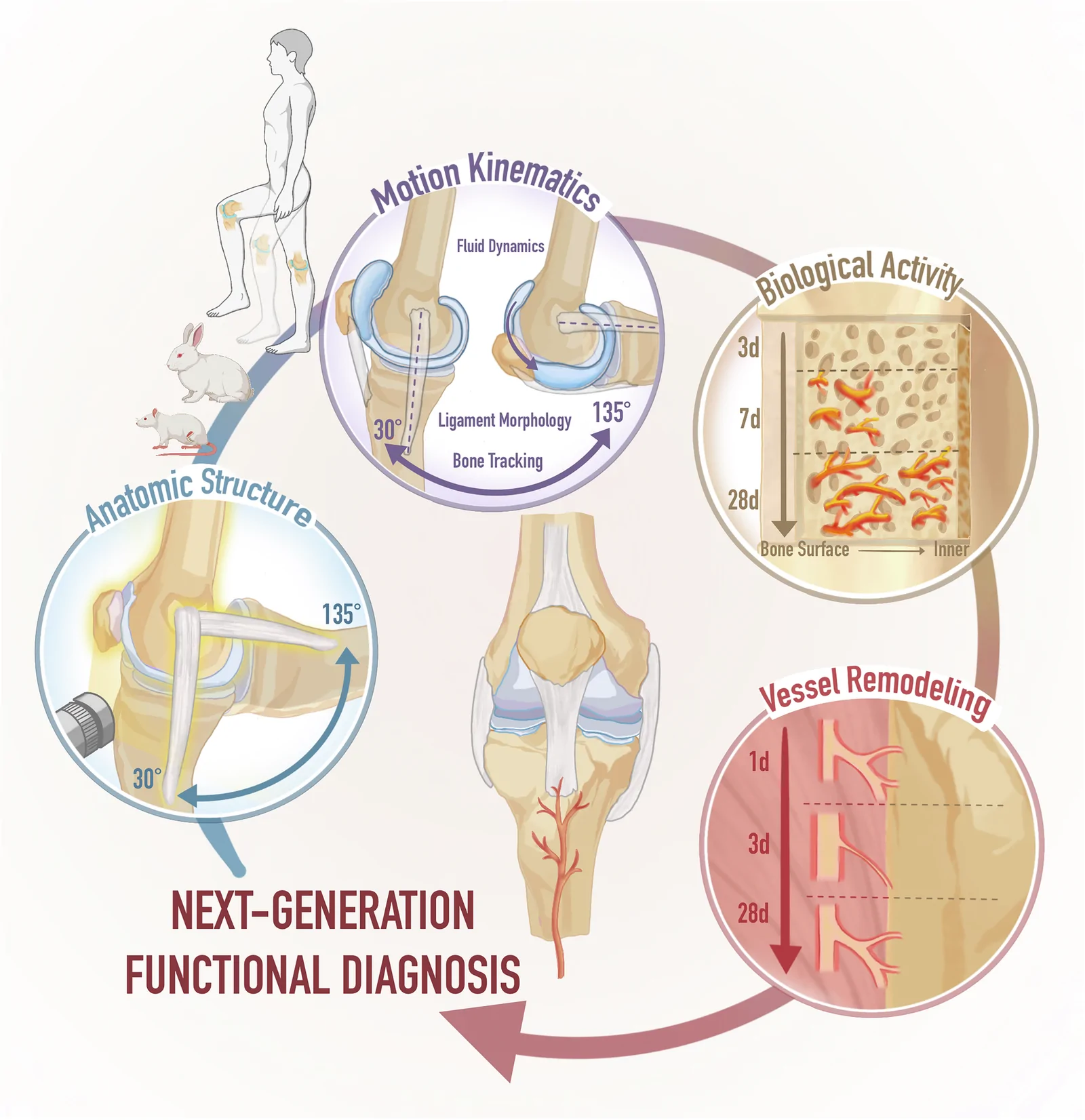
Holistic assessment of the musculoskeletal system, including anatomic structures, kinematics, biological activity, and vessel remodeling. Part of icons were created in BioRender. Li, H. (2026) https://BioRender.com/oj9lhd9.

Following the individual demonstration of SWIR imaging for key musculoskeletal components, including bone, ligament, synovial fluid, and vasculature during locomotion, we performed its ultimate test of synchronous, multi-component imaging. This was achieved by sequential imaging of all the above-mentioned assessments in the same individual mouse in a motion-robust and multi-componential way. Firstly, as shown in Fig. 6, after the intravenous injection of SWIR fluorophores, SWIR vessel imaging (30 s) and bone imaging (24 h) enabled the sequential monitoring of vessel remodeling and biological activity of bone. Next, the intra-articular injection was performed and allowed the visualization of tendon injury as well as kinematics at 24.2 h (Fig. 6 SWIR joint imaging, frontal view), and it could be evaluated concurrently with biological activity after 12 min (Fig. 6 SWIR bone & joint imaging). The real-time visualization during knee flexion and extension was also realized (Supplementary Movie 7), and in vivo (Supplementary Fig. 16a, b) as well as ex vivo (Supplementary Fig. 17a, b) images were captured for confirmation. Notably, the images could be merged for comprehensive evaluation of vessel remodeling, biological activity of bone and joint kinematics. Finally, biosafety of SWIR imaging was confirmed by the analyses of the major organs (Supplementary Fig. 18a–c), which showed normal micro-structures with few remaining SWIR signals. Therefore, as shown in Fig. 7, SWIR imaging was designed for the sequential, motion-robust and multi-componential assessment of musculoskeletal injuries in this study, serving as a perfect candidate for imaging approaches for next-generation functional diagnosis for musculoskeletal diseases.

## Discussion

Based on live SWIR imaging, a holistic assessment of the musculoskeletal system was achieved in this study, delineating anatomic structures, kinematics, and biological activity as well as the spatiotemporal interaction with vessel remodeling. In comparison with conventional imaging modalities such as X-ray, CT, MRI, and ultrasound, the SWIR imaging explored in this study demonstrated significant advantages as an optical imaging method^28,51–53^. This platform affords radio hazard-free, motion-robust imaging analysis with high-resolution and enables multi-component monitoring, thereby ushering in a suite of possibilities to reveal the uncharted secrets of musculoskeletal diseases.

Previous studies have focused on in vivo motion kinematic data acquisition of bone by surface-tracking approaches or fluoroscopy. These data are capable of delineating the biomechanics of bone as well as interpreting neural activity of brain^13,54^. As the surface-tracking proposes the challenge of disconnection of surface marker and internal bones, precise in vivo motion kinematic data acquisition still presents an impediment to studies of biomechanics and neuroscience. Our SWIR joint imaging visualized the deep bone and ligament in a motion-robust way without surface marking, thereby offering a new strategy for this branch of science. Notably, during the motion kinematic analysis, we demonstrated in vivo real-time visual dynamics and velocity data of synovial fluid. Synovial fluid is gaining extensive attention for influencing diseases such as osteoarthritis and rheumatoid arthritis, and its dynamics are usually studied with ex vivo or computational simulation^41,55–59^. As a non-Newtonian fluid, it shows variable viscosity and dynamics depending on the motion type, highlighting the superiority of in vivo data of synovial fluid dynamics^60,61^. Specifically, our results identified a certain knee joint motion with high synovial fluid velocity, which could hinder the repair or graft-bone healing of intra-articular ligaments by interfering with clot formation^62,63^. Further validation of synovial fluid kinematics through precision particle tracking, hydromechanical analysis and studies of other spontaneous disease models, such as degenerative osteoarthritis, would facilitate a more rigorous elucidation of its functional roles in knee joint biomechanics.

Besides the role as a dominator of locomotion, bone is a highly vascularized and dynamic tissue characterized by biological activity of intimate interactions between blood vessels and bones^64^. Studying and understanding these spatiotemporal interplays requires multi-componential imaging modalities. Recently, numerous studies delved into delineating the relationship between angiogenesis and osteogenesis by in vivo multi-photon imaging of small regions or ex vivo light-sheet imaging over large samples with fixation and immunostaining^6,17,65,66^. In vivo SWIR vessel and bone imaging conducted in this study provided a new insight into the in vivo spatiotemporal changes of a large volume of bone with a simple, marker-free and motion-robust way, providing a new strategy that can address vessel and bone biological activity monitoring simultaneously.

The clinical translation of this imaging platform requires consideration of several limitations. First, no FDA-approved SWIR probes are currently available for use in the knee joint, an environment characterized by low serum protein content. Although prior animal studies have demonstrated the safety of the probe used in this work^67^, its toxicity profile in humans remains unclear. Thus, future studies should prioritize FDA-approved probes for clinical application. Second, penetration depth remains a fundamental constraint in optical imaging, although the SWIR window extends depth to approximately 10 mm^68,69^. Notably, both prior human studies^26^ and our cadaveric experiments required skin removal, and deeper structures, including ligaments and meniscus in the human knee, remained unclear. Developing methods with greater penetration would significantly broaden applicability, enabling visualization of deeper anatomy.

Future work in next-generation functional musculoskeletal imaging should include improvement of biocompatibility and penetration depth for further clinical translation by utilizing high-quality probes and high-performance equipment. Moreover, it is expected to encompass organ-crosstalk^70–72^, which can not only monitor the anatomic structures, kinematical function and biological activity of the musculoskeletal system itself, but also shed light on the critical interplaying factors such as vessels, nerves, and lymphatic tissue simultaneously. The previous visualization of lymphatic vessels and nerves bodes well for the potential for combined assessment with live SWIR imaging. These insights enable the early-stage diagnosis of musculoskeletal disorders and facilitate subsequent precision-type intervention.

## Methods

### Preparation of SWIR fluorophores

Highly fluorescent protein-encapsulated lead sulfide QDs were synthesized according to the reported method^73,74^. Briefly, 10 mM Lead (II) acetate trihydrate [Pb(CH_3_CO_2_)_2_·3H_2_O, ≥ 99%] (J&K Scientific, China), 50 mg/mL Ribonuclease-A from bovine pancreas (Sigma-Aldrich, USA) were mixed with 1:1 and 1 M NaOH (Macklin Biochemical Co., Ltd) was added to adjust the pH to 9–10. Then 10 mM Na_2_S (J&K Scientific, China) was added. Deionized water was utilized as the working solution. The tube was put into the microwave reactor (Discover SP, CEM, USA) immediately at 70 °C for 30 s. All freshly prepared QDs were ultra-centrifuged twice with a 10 kDa ultra-centrifugal filter tube to remove excessive reagents. Then the transmission electron microscope (TEM), photoluminescence (PL) spectrum, ultraviolet (UV) spectrum, and dynamic light scattering (DLS) of QDs were measured.

### Experimental model of joint imaging

#### Rat and rabbit

##### Animals

Related animal studies were carried out in agreement with the guidelines approved by the Animal Care Committee of the institution of Fudan University (2025-HSYY-031). Specifically, ten-week-old male SD rats with a weight of 300 g (Shanghai Slac Laboratory Animal Co, Ltd) and sixteen-week-old male New Zealand White rabbits with a weight of 3.0 kg (Shanghai Jiao Tong University agricultural biological experiment practice field) were utilized for the experiments. Rats were maintained in specific pathogen-free conditions in microisolator cages, and all the rabbits were housed under conventional (CV) level conditions. Animals were euthanized with a standard process of anesthesia.

##### Establishment of knee injury animal model

Rats and rabbits were anesthetized with isoflurane (inhalation) and xylazine hydrochloride (injection), respectively.

1) Rat tendon injury (TI) model (superoinferior direction): 1.5 cm of incision was made to the skin after disinfection. Then, the synovial membrane on the patellar tendon was dissected and the whole patellar tendon was cut at the middle of the patellar tendon^75^.

2) Rat combined tendon and muscle injuries (TMI) model (superoinferior and mediolateral direction): According to the previous methods, 1.5 cm of incision was made to the skin after disinfection. Then, the incision was made to the proximal (quadriceps femoris) and distal (tibial tuberosity) endpoints of the patellar tendon. The incision included the vastus medialis, which contributes to patellar mediolateral tracking^76^.

3) Rat MCL inserted bone injury model: After the skin disinfection and incision, fractures at the femur and tibia around the MCL insertion were created with the intact MCL.

4) Rabbit patellar dislocation model: After the skin disinfection and incision, the patella was artificially dislocated by pushing the patella to the lateral (dislocation, in 0.09 s/4 frames) during joint imaging in the knee extension position.

Human: Human cadaveric specimens were obtained through the Body Donor Program of the School of Medicine, Shanghai Jiao Tong University. Written informed consent for the donation and subsequent use of the body for medical education and scientific research was obtained from the donor. The study protocol was approved by the Red Cross Society of China, Shanghai Branch. One fresh human cadaveric  knee joint was used for the SWIR joint imaging.

### Experimental model of vessel and bone imaging

#### Animals

8-week-old male ICR mice with a weight of 30 g were included in the experiment, and three of them were euthanatized for ex vivo analysis at 3 d, 7 d and 28 d post-surgery, respectively. Mice were maintained in specific pathogen-free conditions in microisolator cages. Animals were euthanized with a standard process of anesthesia.

#### Establishment of tibial defect animal model

The tibial defect model was modified from the previous study^77^. Briefly, 1 cm of incision was made to the skin after disinfection. Then, the muscles were detached to visualize the tibia, and drill head with the diameter of 1.5 mm was used to make the tibia defect hole with the diameter of 1.5 mm to the upper tibia. The tissue was washed with saline for several times, and the skin was sutured. The contralateral limb was analyzed as a control. At 3 d, 7 d and 28 d post-surgery, the bone tissue was dissected and SWIR images were taken for further confirmation.

### Experimental model of sequential imaging

Three independent animals of 8-week-old male C57BL/6 mouse with a weight of 25 g were included in the experiments. A combined model of tibial defect and patellar tendon injury (TI) was constructed according to the above-mentioned protocols.

### SWIR animal joint imaging

#### Imaging settings

The SWIR fluorescence images were obtained with the excitation of 808 nm laser with total power of 8.2 W, which was magnified to a circle with a diameter of 6 cm. A 1100 nm long-pass filter was applied, and the emitted fluorescence signal was caught by an InGaAs camera, Goldeye G-130 (Allied Vision, Germany).

#### In vivo

SWIR images and videos were obtained immediately after articular injection of 50 μL (murine-rat) and 100 μL (rabbit) of SWIR fluorophores from the medial, lateral and frontal views (Field of view: 6.3 × 5.0 cm, Spatial resolution:1280 × 1024 pixels, Temporal resolution: 30 ms). Joints were artificially conducted to flexion and extension (30°–135°) with uniform velocity within the time period of 1.38 s. Along with the SWIR imaging, the bright-field macroscopy imaging after the exposure of the patella and MCL was recorded for the confirmation of the accuracy of SWIR imaging.

#### Ex vivo

SWIR imaging of knee anatomic structures was performed with layer-by-layer dissection and also by completely dissecting the femur, tibia, quadriceps femoris, MCL, fat pad, synovial membrane, patellar tendon, LCL and meniscus, respectively, after 30 min of articular injection with the exposure time of 100 ms. The above-mentioned anatomic structures were rinsed in saline for 3 d to perform second imaging.

#### SWIR fluorophores clearance

After the intra-articular injection of SWIR fluorophores to the rat knee, SWIR joint imaging was performed at 5 s, 30 min, 90 min, 120 min, 180 min, 240 min, 3 d and 7 d for the same individual animal.

### SWIR human joint imaging

Total of 3 mL SWIR fluorophores was diluted in saline to 20 mL and injected into the cadaveric left knee joint. Ex vivo SWIR images and videos were obtained immediately after the intra-articular injection with skin removal and superficial adipose tissue from the medial view (Field of view: 6.3 × 5.0 cm, Spatial resolution: 1280 × 1024 pixels, Temporal resolution: 30 ms). The knee joint was artificially conducted to flexion and extension (0°–90°) with uniform velocity within the time period of 1.30 s.

### SWIR vessel and bone imaging

#### In vivo

SWIR images and videos were obtained immediately after intravenous injection of 300 μL SWIR fluorophores for vessel imaging, and bone images were obtained after 24 h of intravenous injection (Field of view: 12.7 × 10.1 cm, Spatial resolution: 1280 × 1024 pixels, Temporal resolution: 500 ms). The imaging process was repeatedly conducted at 1 d, 3 d, 7 d, 14 d, 28 d post-surgery, and animals were immobilized by taping the limbs at the same position of the imaging bed for each imaging.

#### Ex vivo

SWIR imaging was also performed after dissecting the bones (Field of view: 6.3 × 5.0 cm, Spatial resolution: 1280 × 1024 pixels, Temporal resolution: 500 ms).

### SWIR sequential imaging

#### In vivo

After the intravenous injection of 300 μL of SWIR fluorophores, vessel imaging and bone imaging were performed with the temporal resolution of 500 ms at the optimal time window of 30 s and 24 h, respectively (Field of view: 12.7 × 10.1 cm, Spatial resolution: 1280 × 1024 pixels). Subsequently, after the articular injection of 5 μL of SWIR fluorophores (murine, mouse), joint imaging was performed with the temporal resolution of 30 ms from both medial and frontal views. Then dual imaging images were obtained 12 min after the articular injection with the temporal resolution of 30 ms (joint) and 500 ms (bone) at the same position.

#### Ex vivo

After the in vivo sequential imaging, muscles were dissected, and ex vivo SWIR imaging of bone and joints was performed (Field of view: 6.3 × 5.0 cm, Spatial resolution: 1280 × 1024 pixels, Temporal resolution: 30 ms for joint imaging and 500 ms for bone imaging).

### MRI and Micro-CT

Rats were anesthetized for MRI (7.0 T, Novila, Shanghai Chenguang Medical Technologies Co., Ltd.) and micro-CT (SkyScan1276, Bruker) analysis. For MRI, the T2-weighted sequence was performed in sagittal, coronal and axial orientation (TE: 52.56 ms, TR: 3 s, Slice thickness: 600 μm, Matrix 320 × 320, FOV: 55 × 55 mm). Micro-CT scans were performed with Source voltage: 90 kV, Source current: 200 μA, and the data were reconstructed with Nrecon and visualized with CVox software. For the tibial defect mouse model, ex vivo micro-CT imaging and analysis were performed with harvested bones at 3 d, 7 d and 28 d post-surgery.

### Histological analysis

#### Vessel and bone imaging

Bones were harvested at 3 d, 7 d and 28 d post-surgery to perform histological analysis of hematoxylin-eosin (HE) staining, Masson’s trichrome staining and immunofluorescence staining. Immunofluorescence staining was performed with anti-CD31 antibody (Servicebio, GB113151, 1:3000), anti-α-SMA antibody (Servicebio, GB111364, 1:2000), Cy3 conjugated Goat Anti-Rabbit IgG (Servicebio, GB21303, 1:300) and Alexa Fluor® 488-conjugated Goat Anti-Rabbit IgG (Servicebio, GB25303, 1:300).

#### Biosafety analysis

For three kinds of imaging, the major organs, including the lung, heart, stomach, kidney, spleen, liver and intestine were harvested, and SWIR images were taken at 7 d (joint) and 14 d (bone) after the last injection. Then, the HE staining of major organs was performed for histological analysis.

### Data analysis

Data analysis and processing were performed by ImageJ (Version 2.3.0), MATLAB R2020b, Python (version 3.10) and GraphPad Prism 9.

#### Joint Imaging

The knee flexion angles were determined through frame analysis of motion videos by measuring the femoral-tibial angle and were verified independently by three experienced surgeons. The key anatomic structures were artificially marked with colors, and original images without pseudo colors were shown in Supplementary Fig. 19. Signal-to-background ratio (SBR) was calculated by dividing the FL intensity of target tissues by surrounding tissues after inverting the image. Data from six independent animals were included in the data analysis, and three independent animals for synovial fluid analysis.

##### Synovial fluid

1) Subregion analysis: The processing figure was shown in Supplementary Fig. 20, and segmentation results for a representative whole cycle of knee flexion and extension were demonstrated in Supplementary Movie 8. Briefly, each frame extracted from the video was preprocessed with noise reduction (gaussian_filter, SciPy library) and contrast enhancement (adjust_log, SciKit-Image library). Then, the knee was separated (threshold_otsu, SciKit-Image library). Next, four specific points are manually selected to form 2 lines, and the knee is automatically divided into the suprapatellar bursa (left, blue), outflow tract (middle, white), and joint cavity (right, orange) according to the lines. Finally, boundaries of subregions were visualized (mark_boundaries, SciKit-Image library) across the entire video, and the total intensity value of fluorescence was calculated for each section and frame. 2) Velocity: With the frames demonstrating knee flexion degree of 90°–100°, a sudden flow of synovial fluid (a cluster of fluorescence) could be visualized. The velocity of synovial fluid was calculated by dividing the displacement of synovial fluid by time. Additionally, for extracted frames demonstrating knee flexion degree of 90°–135°, SWIR FL intensity of each pixel of the outflow tract (total length of 8 mm), which was located between suprapatellar bursa (0 mm) and joint cavity (8 mm), was measured by ImageJ (Version 2.3.0) and a 3D graph was visualized by MATLAB R2020b.

##### MCL

The length of the medial collateral ligament (MCL), the transaction angle between MCL and femur, and MCL and tibia were measured from the SWIR images from the medial view after extracting frames from both SWIR imaging and bright-field macroscopy imaging. Images were selected for measurements at every 10° of knee flexion. For each image, MCL, femur, and tibia were identified, and the length of MCL was measured between the inserted femur and tibia, and the intersection angle between MCL-femur and MCL-tibia was calculated. The MCL elongation pattern and intersection angle change pattern were then compared between those measured using SWIR imaging and bright-field microscopy for confirmation.

##### Patella

Similarly, images were extracted from the video, and for each frame, the patella and femur were identified. The distance from the top of the patella to the bottom of the femur (superoinferior) as well as the distance from the medial edge of the patella to the extended line of the medial edge of the femur (mediolateral) were measured from the frontal view. Then the patella was compared between that measured using SWIR imaging and bright-field microscopy for confirmation.

##### Vessel and bone imaging

For each time point, all available quantitative data from three to six independent animals were included in the data analysis. 1) Vessel diameter: The FL intensity of a line perpendicular to a single vessel was measured from fluorescent images with ImageJ (Version 2.3.0), and the full width at half maximum (FWHM) of Gaussian fitting was calculated as the vessel diameter using Origin. 2) Perfusion ratio: Dividing the FL intensity of the injured limb by that of the control side. 3) Bone: The FL intensity of the whole tibial defect area, length, width and area of remained tibial defect area were measured using ImageJ (Version 2.3.0). For each micro-CT scanning of the tibial defect, parameters including bone mineral density (BMD), trabecular number (Tb. N), Trabecular thickness (Tb. Th), and trabecular separation (Tb. Se) were calculated.

### Statistical analysis

The statistical analysis and visualization were performed with GraphPad Prism 9. The significance between the two groups was analyzed with a student *t* test, and the data from multiple groups were analyzed using a one-way ANOVA test. Repeated measurements of the same group of samples at multiple time points were analyzed with repeated measures ANOVA or mixed-effects analysis. P value < 0.05 was considered statistically significant. Simple linear regression was utilized for patellar tracking quantification. Correlation analysis was performed with Pearson’s correlation matrix.

### Reporting summary

Further information on research design is available in the Nature Portfolio Reporting Summary linked to this article.

## Supplementary information

Supplementary Information

Description of Additional Supplementary Files

Supplementary Movie 1

Supplementary Movie 2

Supplementary Movie 3

Supplementary Movie 4

Supplementary Movie 5

Supplementary Movie 6

Supplementary Movie 7

Supplementary Movie 8

Reporting Summary

Transparent Peer Review file

## Source data

Source Data

## Data Availability

All data supporting the findings of this study are available within the article and its supplementary files. Any additional requests for information can be directed to and will be fulfilled by the corresponding authors. Figshare https://doi.org/10.6084/m9.figshare.29857028. Source data are provided with this paper.
