# Supplementary material for: Next-generation functional diagnostic paradigm for musculoskeletal diseases based on live SWIR imaging
Source: Nat Commun. 2026 Sep 14;17:11269. doi: 10.1038/s41467-026-77137-4 (PMC13720147; doi:10.1038/s41467-026-77137-4)
Supplement: Supplementary file 1 [file 41467_2026_77137_MOESM11_ESM.pdf]

nature portfolio

Corresponding author(s): Yueming Wang, Fuchun Chen, Yunxia Li, Jun Chen

Last updated by author(s): May 7, 2026

Reporting Summary

Nature Portfolio wishes to improve the reproducibility of the work that we publish. This form provides structure for consistency and transparency in reporting. For further information on Nature Portfolio policies, see our Editorial Policies and the Editorial Policy Checklist.

Statistics

For all statistical analyses, confirm that the following items are present in the figure legend, table legend, main text, or Methods section.

n/a	Confirmed	
☐	☒	The exact sample size (*n*) for each experimental group/condition, given as a discrete number and unit of measurement
☐	☒	A statement on whether measurements were taken from distinct samples or whether the same sample was measured repeatedly
☐	☒	The statistical test(s) used AND whether they are one- or two-sided<br>*Only common tests should be described solely by name; describe more complex techniques in the Methods section.*
☒	☐	A description of all covariates tested
☒	☐	A description of any assumptions or corrections, such as tests of normality and adjustment for multiple comparisons
☐	☒	A full description of the statistical parameters including central tendency (e.g. means) or other basic estimates (e.g. regression coefficient) AND variation (e.g. standard deviation) or associated estimates of uncertainty (e.g. confidence intervals)
☐	☒	For null hypothesis testing, the test statistic (e.g. *F*, *t*, *r*) with confidence intervals, effect sizes, degrees of freedom and *P* value noted<br>*Give P values as exact values whenever suitable.*
☒	☐	For Bayesian analysis, information on the choice of priors and Markov chain Monte Carlo settings
☒	☐	For hierarchical and complex designs, identification of the appropriate level for tests and full reporting of outcomes
☐	☒	Estimates of effect sizes (e.g. Cohen's *d*, Pearson's *r*), indicating how they were calculated

Our web collection on statistics for biologists contains articles on many of the points above.

Software and code

Policy information about availability of computer code

Data collection	In vivo and ex vivo SWIR imaging data were acquired with Acquired Control V6.0.0. In vivo micro-CT and MRI imaging data were acquired using commercial software associated with the imaging hardware employed, including Skysan 1276 for micro-CT and Prim for MRI.
Data analysis	Data analysis was performed using FIJI 2.3.0 (Image J and preinstalled plugins), GraphPad Prism 9, MATLAB R 2020b, Phython 3.10,

For manuscripts utilizing custom algorithms or software that are central to the research but not yet described in published literature, software must be made available to editors and reviewers. We strongly encourage code deposition in a community repository (e.g. GitHub). See the Nature Portfolio guidelines for submitting code & software for further information.

Data

Policy information about availability of data

All manuscripts must include a data availability statement. This statement should provide the following information, where applicable:

- Accession codes, unique identifiers, or web links for publicly available datasets
- A description of any restrictions on data availability
- For clinical datasets or third party data, please ensure that the statement adheres to our policy

All data supporting the findings of this study are available within the article and its supplementary files. Source data are provided with this paper. Any additional requests for information can be directed to, and will be fulfilled by, the corresponding authors. Figshare DOI: https://doi.org/10.6084/m9.figshare.29857028

Research involving human participants, their data, or biological material

Policy information about studies with human participants or human data. See also policy information about sex, gender (identity/presentation), and sexual orientation and race, ethnicity and racism.

Reporting on sex and gender	The study utilized one male cadaveric specimen.
Reporting on race, ethnicity, or other socially relevant groupings	The study utilized one cadaveric specimen from an individual of Asian.
Population characteristics	The study utilized one male cadaveric specimen with the age of 90 and multiple cartilage leisions in knee joint.
Recruitment	The cadaveric body is donation to the School of Medicine, Shanghai Jiao Tong University.
Ethics oversight	The study protocal was approved by the Red Cross Society of China, Shanghai Branch.

Note that full information on the approval of the study protocol must also be provided in the manuscript.

Field-specific reporting

Please select the one below that is the best fit for your research. If you are not sure, read the appropriate sections before making your selection.

☒ Life sciences ☐ Behavioural & social sciences ☐ Ecological, evolutionary & environmental sciences

For a reference copy of the document with all sections, see nature.com/documents/nr-reporting-summary-flat.pdf

Life sciences study design

All studies must disclose on these points even when the disclosure is negative.

Sample size	No predetermination of sample sizes was done.
Data exclusions	Unmeasurable vessel diameter data were excluded in the data analysis in the vessel imaging section.
Replication	All data were biologically replicated at least 3-6 times. See method section in the main text.
Randomization	Animals were allocated into control or experimental groups randomly.
Blinding	To conduct the experiment, the authors were not blinded.

Reporting for specific materials, systems and methods

We require information from authors about some types of materials, experimental systems and methods used in many studies. Here, indicate whether each material, system or method listed is relevant to your study. If you are not sure if a list item applies to your research, read the appropriate section before selecting a response.

Materials & experimental systems

n/a	Involved in the study	
☐	☒	Antibodies
☒	☐	Eukaryotic cell lines
☒	☐	Palaeontology and archaeology
☐	☒	Animals and other organisms
☒	☐	Clinical data
☒	☐	Dual use research of concern
☒	☐	Plants

Methods

n/a	Involved in the study	
☒	☐	ChIP-seq
☒	☐	Flow cytometry
☒	☐	MRI-based neuroimaging

Antibodies

Antibodies used	anti-CD31 antibody (Servicebio, GB113151, 1:3000), anti-α-SMA antibody (Servicebio, GB111364, 1:2000), Cy3 conjugated Goat Anti-Rabbit IgG (Servicebio, GB21303, 1:300) and Alexa Fluor® 488-conjugated Goat Anti-Rabbit IgG (Servicebio, GB25303, 1:300)
Validation	All antibodies were commercially available.

Animals and other research organisms

Policy information about studies involving animals; ARRIVE guidelines recommended for reporting animal research, and Sex and Gender in Research

Laboratory animals	ten-week-old male SD rats with the weight of 300 g, sixteen-week-old male New Zealand White rabbits with the weight of 3.0 kg, 8-week-old male ICR mice with the weight of 30 g, 8-week-old male C57BL/6 mouse with the weight of 25 g
Wild animals	No wild animals were used in the study.
Reporting on sex	All animals included in this study were male, in order to exclude the potential hormonal effects associated with female animals.
Field-collected samples	No field collected samples were used in the study.
Ethics oversight	Related animal studies were carried out in agreement with the guidelines approved by the Animal Care Committee of Fudan University (2025-HSYY-031).

Note that full information on the approval of the study protocol must also be provided in the manuscript.

Plants

Seed stocks	*Report on the source of all seed stocks or other plant material used. If applicable, state the seed stock centre and catalogue number. If plant specimens were collected from the field, describe the collection location, date and sampling procedures.*
Novel plant genotypes	*Describe the methods by which all novel plant genotypes were produced. This includes those generated by transgenic approaches, gene editing, chemical/radiation-based mutagenesis and hybridization. For transgenic lines, describe the transformation method, the number of independent lines analyzed and the generation upon which experiments were performed. For gene-edited lines, describe the editor used, the endogenous sequence targeted for editing, the targeting guide RNA sequence (if applicable) and how the editor was applied.*
Authentication	*Describe any authentication procedures for each seed stock used or novel genotype generated. Describe any experiments used to assess the effect of a mutation and, where applicable, how potential secondary effects (e.g. second site T-DNA insertions, mosiacism, off-target gene editing) were examined.*
