# Supplementary material for: Next-generation functional diagnostic paradigm for musculoskeletal diseases based on live SWIR imaging
Source: Nat Commun. 2026 Sep 14;17:11269. doi: 10.1038/s41467-026-77137-4 (PMC13720147; doi:10.1038/s41467-026-77137-4)
Supplement: Supplementary file 2 [file 41467_2026_77137_MOESM12_ESM.pdf]

nature portfolio

Peer Review File

Next-generation functional diagnostic paradigm for musculoskeletal diseases based on live SWIR imaging

Corresponding Author: Professor JUN CHEN

This file contains all reviewer reports in order by version, followed by all author rebuttals in order by version.

Version 0:

Reviewer comments:

Reviewer #1

(Remarks to the Author)
I read the manuscript “Next-generation functional diagnostic paradigm for musculoskeletal diseases based on live SWIR imaging” with great interest. The novel imaging approach proposed by the authors for kinematic visualization of the skeletomuscular system introduces a promising avenue for assessing musculoskeletal abnormalities and injuries. Specifically, it enables real-time, in vivo assessment of dynamic tissue behavior. This is extremely important, as the function of structures such as menisci, ligaments, tendons, and synovial fluid can reveal pathologies that remain undetectable under static conditions. For example, back pain - one of the most common musculoskeletal complaints, experienced by nearly one-third of the global population - often lacks clear findings on static imaging. Thus, the presented work provides a solid scientific foundation for developing more robust and accurate diagnostic approaches to musculoskeletal pathologies.

The manuscript is well written and methodologically sound. The data are presented clearly and thoroughly, and several complementary methods are employed to substantiate the authors’ conclusions.

Overall, I find this manuscript to be of high scientific quality and potential impact. However, several issues should be addressed before it reaches publication quality.

Major comments
1. Lines 15–16: “This enabled the monitoring of angiogenesis, and revealed a decoupling from bone regeneration.”
This statement is an overinterpretation. While SWIR imaging shows vascular dynamics that do not coincide temporally with bone healing, the method detects only large arteries, whereas osteoangiogenic coupling occurs primarily at the microvascular level (e.g., H-type vessels; PMID: 24646994). Therefore, this conclusion cannot be drawn. Moreover, when angiogenesis was assessed using the pan-endothelial marker CD31, the strongest correlation with bone healing was observed.

2. Figure 5 and related text (lines 422–437):
The authors attempt to explain the apparent discrepancy between the healing pattern (from inside-out) and the SWIR signal (from outside-in). They correlate this with vascularization, citing CD31 and αSMA staining, and conclude that “the SWIR FL signals point to the biological activity of vascularization.” However, the SWIR signal at this time point is unlikely to originate from vessels directly, and the αSMA signal shown does not exhibit vascular morphology. The authors should seriously consider that the signal may instead arise from αSMA-positive periosteal cells, which are known to contribute substantially to fracture repair (PMID: 24190076). It is plausible that SWIR detects these cells during their early proliferative or migratory expansion. Imaging of proliferating or migrating periosteal cells would help clarify this point.

3. Discussion – limitations:
A discussion of the limitations of the proposed imaging approach is missing and should be included. In particular:
a) Potential toxicity of the SWIR fluorophores must be discussed in relation to possible human applications. Figure S18 provides some relevant data, but it does not constitute a toxicological study. Have these fluorophores been tested or approved for human use?
b) What is the penetration depth limit of far-red/near-infrared light in human tissues? Figure 3g,h, and Video 7 are impressive but do not visualize ligaments, suggesting that deeper knee structures may not be detectable in humans due to the physical limits of light penetration. Please address these limitations explicitly in the Discussion.

Minor comments
• Line 153:
"As an essential factor influencing the progression of osteoarthritis and rheumatoid arthritis …"
Please provide a reference supporting this statement.

• Figure 1c:
The arrows appear misaligned and should be corrected.

• Flexion/extension angle labeling:
The terminology and labeling are confusing. Although the explanation is reasonable, the graphical representation should be clearer. It would be helpful to annotate one of the relevant figures (e.g., Fig. 1j, p, or q) or to expand Fig. S6 with a schematic. As I understand it, flexion = 135 % corresponds to the most bent position, and flexion = 30 % to the most extended knee. If so, please verify that Figure 1p correctly shows that the MCL is longest when the knee is straight. This appears inconsistent with the text and with Figure S7C. Please clarify throughout.

• Lines 313–315:
The explanation for the abnormal kinematics observed during patellar dislocation is unclear. Please elaborate.
The corresponding methods section also requires clarification—specifically, why time intervals of 0.03, 0.06, and 0.09 seconds were chosen, and whether the knee was flexed during these recordings.

• Human knee experiment:
This part is particularly impressive. Please clarify in the main text whether the skin was removed prior to imaging and how long after fluorophore injection imaging was performed.

• Figure 4b legend:
The statement "with tail-vein injection of SWIR fluorophores at 30 s post-injection" is incorrect. Please revise to indicate that imaging was performed 30 seconds after tail-vein injection, not that the injection occurred 30 seconds post-injection.

• Lines 373–374:
"Therefore, SWIR vessel imaging enabled the longitudinal monitoring of spatiotemporal vessel remodeling and the identification of the critical time point."
Measuring diameters of two large arteries does not support a claim of vessel remodeling. Please rephrase more conservatively.

• Lines 390–392:
"3 d – 7 d post-surgery was identified as a critical phase, coordinating with the temporal sequence of vessel remodeling."
The data supporting this statement are unclear. Please clarify the basis for identifying this specific time window as a "critical phase."

• Lines 553–554:
"Human: The cadaveric body is the donation to the institution, and the study protocol was approved by the committee."
Please specify the full name of the approving ethics or institutional review committee.

Reviewer #2

(Remarks to the Author)
This manuscript presents a SWIR imaging method for assessment of joint motion and functional diagnosis of musculoskeletal diseases. As one non-invasive, deep penetration and in vivo modality, the advances of SWIR imaging have been witnessed in the past decade, this topic is definitely important and could have impact on living tissue imaging, especially for skeletons deep inside the body. The highlight of this work is using SWIR imaging for the motion kinematics study, however, to my understanding, a state of free movement is necessary instead of anesthesia, as the muscle, tendon or bones do not exert force under anesthesia, this study ignored the joint motion during the free movement. Meanwhile, I am a bit confused on the knee joint images under different flexion angles, such a lot of results give no obvious differences.
The study goes on to report the in vivo imaging on the healing of tibial defect for 28 days, which proves the advantages of the SWIR imaging for long-term application. In general, the manuscript is interesting, but lacks convincing results and a high degree of novelty, the experimental results on musculoskeletal system should be published on a more specialized journal.
I have the following comments/questions:
1. Fig 1f shows the SWIR fluorophores could be excreted out in 7 days after the intra-articular injection, and the SWIR emission vanished in 2 hours. What is the metabolic pathway of the SWIR fluorophores? How is the excretability of fluorophores achieved?
2. The manuscript provides few information on the SWIR fluorophores, as the cited reference synthesized SWIR fluorophores for detecting bacteria, is there any difference between them?
3. After the intra articular injection, how do the fluorophores label the tissues?
4. Do the SWIR fluorophores have any targeting ability to the bones or other tissues?
If not, how to distinguish different tissues in the joint? Like the ligament, meniscus, and patella.
5. In Fig 2i, how to manipulate the angle of joint flexion at 30°, 60°, 90°, and 135°? Is the approach precise enough to assess the overall MCL spatial displacement? As these assessments depend on the flexion angles very much.

6. SWIR skeletal imaging is a rapidly emerging field, some representative papers in this field should be properly cited, such as Nature Communications 2025, 16, 4911 (SWIR knee joint imaging); Nature Communications 2023, 14, 6287 (bone defect and disease imaging); Nano Lett. 2021, 21, 1, 798–805 (NIR-II skeleton imaging by polymer nanoparticles); Nano Lett. 2019, 19, 5, 2985–2992.

Reviewer #3

(Remarks to the Author)
This manuscript presents a technically ambitious and conceptually novel imaging platform using live short-wave infrared (SWIR) fluorescence imaging to achieve real-time, motion-robust, multi-component functional assessment of the musculoskeletal system. The central premise is that conventional imaging modalities, including X-ray, CT, MRI, and ultrasound, are limited by static acquisition, poor temporal resolution, or the inability to capture multiple interacting functional components during locomotion. The authors propose live SWIR imaging as a next-generation diagnostic paradigm capable of simultaneously visualizing deep anatomic structures, motion kinematics, synovial fluid dynamics, vascular remodeling, and bone biological activity in vivo.

The work is structured as a progressive demonstration of capability. The authors first establish that SWIR joint imaging, following intra-articular injection of SWIR fluorophores, can non-invasively visualize deep knee joint structures in rats without the need for dissection, achieving millisecond-scale temporal resolution. They then exploit this capability to quantify normal motion kinematics during knee flexion, including synovial fluid circulation, ligament elongation and angular displacement, and patellar tracking. These measurements are validated against macroscopic observations and shown to capture dynamic behaviors that are inaccessible to static MRI or CT.

The platform is then applied to functional diagnosis in injury models, including isolated patellar tendon injury and combined tendon–muscle injury. Using patellar tracking slopes and directional displacement metrics, the authors demonstrate that SWIR imaging can discriminate not only between the presence of injury but also between injury types and severities based on kinematic signatures rather than anatomy alone. Importantly, they further show that ligament functional impairment can be detected in a bone injury model even when the ligament remains anatomically intact, underscoring the sensitivity of the approach to early functional pathology.

The manuscript then expands its translation to rabbit knees and a human cadaveric knee, demonstrating that SWIR imaging can visualize joint structures through intact skin and capture abnormal kinematics, such as patellar dislocation and mechanical locking during extension. Finally, the authors extend the platform beyond joints to longitudinal monitoring of vascular remodeling and bone healing in a mouse tibial defect model. By exploiting distinct post-injection imaging windows, they sequentially image vessels and bone, revealing a temporal decoupling between angiogenesis and bone regeneration. This demonstrates that the SWIR bone signal correlates more strongly with vascular biological activity than with static bone volume measured by micro-CT. The study culminates in a holistic, sequential imaging workflow integrating vessel remodeling, bone biological activity, and joint kinematics in the same animal.

The strengths of this work are substantial. Conceptually, it reframes musculoskeletal imaging around function rather than static structure, convincingly arguing that many clinically relevant pathologies manifest primarily during motion. Technically, the imaging platform achieves an unusual combination of penetration depth, temporal resolution, and multi-component sensitivity that is not available with existing modalities. The breadth of experimental validation is impressive, encompassing multiple species, various injury models, and diverse functional domains. The quantitative analyses of synovial fluid dynamics, ligament elongation, and patellar tracking are particularly compelling, as is the demonstration that SWIR bone signal reflects biological activity rather than simply mineralized tissue. The figures are generally clear, information-dense, and well aligned with the narrative, and the study is ambitious in scope while remaining internally coherent.

Despite these strengths, the manuscript has several significant limitations that need to be addressed.
The most significant concern is the small sample size in most experiments, with many key conclusions based on n = 3 animals per group. While understandable, given the technical demands, this limits statistical power and raises concerns about robustness, particularly for claims regarding diagnostic sensitivity, injury discrimination, and temporal “critical phases” of healing. Variability is not always fully reported, and some statistical comparisons appear underpowered relative to the strength of the conclusions drawn.

A second major limitation is the reliance on exogenous SWIR fluorophores, including lead sulfide quantum dots, which raises substantial translational and regulatory concerns. While clearance and short-term biosafety are addressed, the manuscript does not sufficiently discuss long-term toxicity, repeat-dose feasibility, or clinical alternatives. This omission weakens claims of near-term clinical applicability, particularly when the human data are limited to a single cadaveric specimen.
Third, while the authors repeatedly emphasize “diagnostic” capability, the study stops short of rigorous diagnostic validation. There is no comparison against gold-standard clinical diagnostic criteria, no receiver-operating characteristic analysis, and no demonstration of sensitivity or specificity across heterogeneous disease states. The injury models are well-controlled but relatively artificial, and it remains unclear how the proposed metrics would generalize to spontaneous or degenerative human diseases.

The manuscript also suffers from overextension in places. The scope is extremely broad, covering joints, ligaments, synovial fluid, vasculature, and bone healing. While each component is individually interesting, the cumulative effect is a very long

and dense manuscript that risks obscuring its core contributions. The discussion occasionally overstates clinical implications without fully acknowledging the technological, biological, and regulatory gaps that remain. Additionally, the methods section is lengthy and technically complex and would benefit from clearer organization and schematic summarization to improve reproducibility.

To improve the manuscript, the authors should temper their claims regarding diagnostic readiness and emphasize the work as a potentially powerful preclinical and translational platform rather than a near-clinical diagnostic tool.

Expanding sample sizes for key experiments, or at the very least, providing stronger justification and power analysis, would substantially enhance confidence in the findings.

A more explicit discussion of fluorophore safety, alternative contrast strategies, and regulatory pathways is essential. The diagnostic claims would be more convincing if supported by formal performance metrics or blinded classification tasks. Finally, tightening the narrative to focus more clearly on the unifying concept of motion-robust, multi-component functional imaging—rather than repeatedly re-demonstrating capability—would improve clarity and impact.

Version 1:

Reviewer comments:

Reviewer #1

(Remarks to the Author)
I would like to thank the authors for addressing most of my concerns. While the manuscript has improved following revision, I still believe it is important to clearly outline the limitations of the approach within the manuscript itself, rather than only in the response to my previous comment (Major Comment 3 in the earlier review).

Explicit discussion of these limitations would help readers appropriately interpret the findings and place the results in their proper context. I therefore strongly encourage the authors to include a dedicated paragraph in the Discussion that explicitly addresses the potential limitations of the study.

Reviewer #2

(Remarks to the Author)
In the revised manuscript, the authors have addressed all previous suggestions, and the overall quality has significantly improved. I recommend publication after the following minor concerns are addressed:

1. The methodology repeatedly refers to the use of “all available quantitative data,” yet the number of biological replicates varies considerably across different time points. The authors should clarify the reasons for the changes in sample size (n-value) over time and specify whether any sample exclusion criteria were applied. (Lines 713–724)

2. While the study demonstrates intra-articular clearance and includes HE analysis of major organs in the methodology, the use of quantum dot PbS raises additional safety considerations. More comprehensive evidence regarding the biosafety of the materials used is recommended.

3. The relevance of the disease models should be strengthened. Figure 2 employs an acute injury model (tendon transection and fracture), which does not accurately reflect chronic degenerative diseases such as osteoarthritis or rheumatoid arthritis. Nevertheless, the manuscript repeatedly refers to the diagnostic relevance of such conditions, indicating a weak alignment between the model and the intended clinical context. Similarly, the tibial defect model in Figures 4–5 represents a bone regeneration paradigm rather than a bone disease model. Its suitability for demonstrating functional diagnosis of bone diseases should be justified more clearly.

Reviewer #3

(Remarks to the Author)
The authors have provided appropriate responses to all queries raised during the initial review. Inasmuch, the manuscript is substantially improved.

Version 2:

Reviewer comments:

Reviewer #2

(Remarks to the Author)
no more comments from this reviewer

Open Access This Peer Review File is licensed under a Creative Commons Attribution 4.0 International License, which permits use, sharing, adaptation, distribution and reproduction in any medium or format, as long as you give appropriate credit to the original author(s) and the source, provide a link to the Creative Commons license, and indicate if changes were made.
In cases where reviewers are anonymous, credit should be given to 'Anonymous Referee' and the source.
The images or other third party material in this Peer Review File are included in the article's Creative Commons license, unless indicated otherwise in a credit line to the material. If material is not included in the article's Creative Commons license and your intended use is not permitted by statutory regulation or exceeds the permitted use, you will need to obtain permission directly from the copyright holder.
To view a copy of this license, visit https://creativecommons.org/licenses/by/4.0/

Dear editors and reviewers,

Thank you for your insightful assessments and constructive feedback on our manuscript entitled "Next-generation functional diagnostic paradigm for musculoskeletal diseases based on live SWIR imaging." (NCOMMS-25-72766-T). We have carefully addressed all the comments and suggestions, and have made corresponding revisions to the manuscript, with all changes highlighted in yellow. We hope that our responses and modifications adequately resolve the reviewers' concerns.

Thank you again for your time and consideration.

Point-to-point responses:

Reviewer #1 (Remarks to the Author):
General Comment:
I read the manuscript "Next-generation functional diagnostic paradigm for musculoskeletal diseases based on live SWIR imaging" with great interest. The novel imaging approach proposed by the authors for kinematic visualization of the skeletomuscular system introduces a promising avenue for assessing musculoskeletal abnormalities and injuries. Specifically, it enables real-time, in vivo assessment of dynamic tissue behavior. This is extremely important, as the function of structures such as menisci, ligaments, tendons, and synovial fluid can reveal pathologies that remain undetectable under static conditions. For example, back pain - one of the most common musculoskeletal complaints, experienced by nearly one-third of the global population - often lacks clear findings on static imaging. Thus, the presented work provides a solid scientific foundation for developing more robust and accurate diagnostic approaches to musculoskeletal pathologies.

The manuscript is well written and methodologically sound. The data are presented clearly and thoroughly, and several complementary methods are employed to substantiate the authors' conclusions.

Overall, I find this manuscript to be of high scientific quality and potential impact. However, several issues should be addressed before it reaches publication quality.

Response: We would like to thank the reviewer for the constructive feedback as well as the encouraging remarks on our work. Below are our point-to-point responses.

Major comments:
1. Lines 15–16: "This enabled the monitoring of angiogenesis, and revealed a decoupling from bone regeneration."
This statement is an overinterpretation. While SWIR imaging shows vascular dynamics that do not coincide temporally with bone healing, the method detects only large arteries, whereas osteoangiogenic coupling occurs primarily at the microvascular level (e.g., H-type vessels; PMID: 24646994). Therefore, this conclusion cannot be drawn. Moreover, when angiogenesis was assessed using the pan-endothelial marker CD31, the strongest correlation with bone healing was observed.
Response:

Thank you for raising these points. In this study, there exists bone regeneration in the area without fluorescence, and fluorescence area was related to CD31 and α-SMA positive areas. To avoid misunderstanding, the text has been revised to "This enabled the monitoring of large arteries in bone, and revealed an initial divergence from the bone regeneration".

Besides, as the reviewer recommended, CD31 and endomucin were stained after the tibia injury in our on-going study (Figure R1). It demonstrated coupling of $CD31^{hi}$/$Endomucin^{hi}$ signals and osterix positive cells, suggesting the coupling of angiogenesis of type H vessels and osteogenesis.

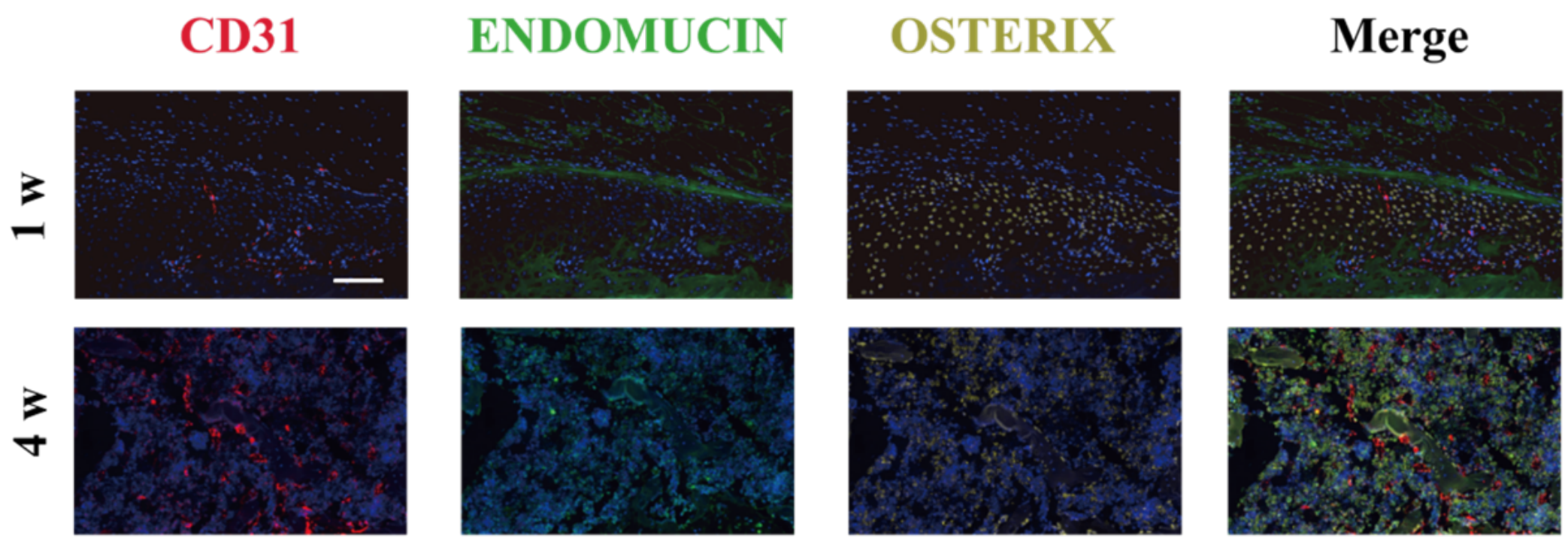

Figure R1. CD31/Endomucin/Osterix immunostaining 1 w and 4 w after the tibial injury. Scale bar: 100 μm.

Revision (Line 15): This enabled the monitoring of large arteries in bone, and revealed an initial divergence from the bone regeneration.

2. Figure 5 and related text (lines 422–437):

The authors attempt to explain the apparent discrepancy between the healing pattern (from inside-out) and the SWIR signal (from outside-in). They correlate this with vascularization, citing CD31 and αSMA staining, and conclude that "the SWIR FL signals point to the biological activity of vascularization." However, the SWIR signal at this time point is unlikely to originate from vessels directly, and the αSMA signal shown does not exhibit vascular morphology. The authors should seriously consider that the signal may instead arise from αSMA-positive periosteal cells, which are known to contribute substantially to fracture repair (PMID: 24190076). It is plausible that SWIR detects these cells during their early proliferative or migratory expansion. Imaging of proliferating or migrating periosteal cells would help clarify this point.

Response: Thank you for your valuable comments regarding the possible origin of the SWIR signal. Numerous publications including our previous study have documented the phenomenon of SWIR bone imaging[1-7]. However, the mechanism is not yet fully elucidated. Current literatures (Table R1) suggest the signal origins from multiple cells, including osteoblasts, perivascular macrophages and endothelial cells in bone marrow sinusoids [1,4-6], indicating the signals may be related to vessels.

Table R1. Possible origin of the SWIR signal in bone.

Study	Results	Conclusion

Li, et al.[5] https://doi.org/ 10.1021/acs.nanolett.1c04531	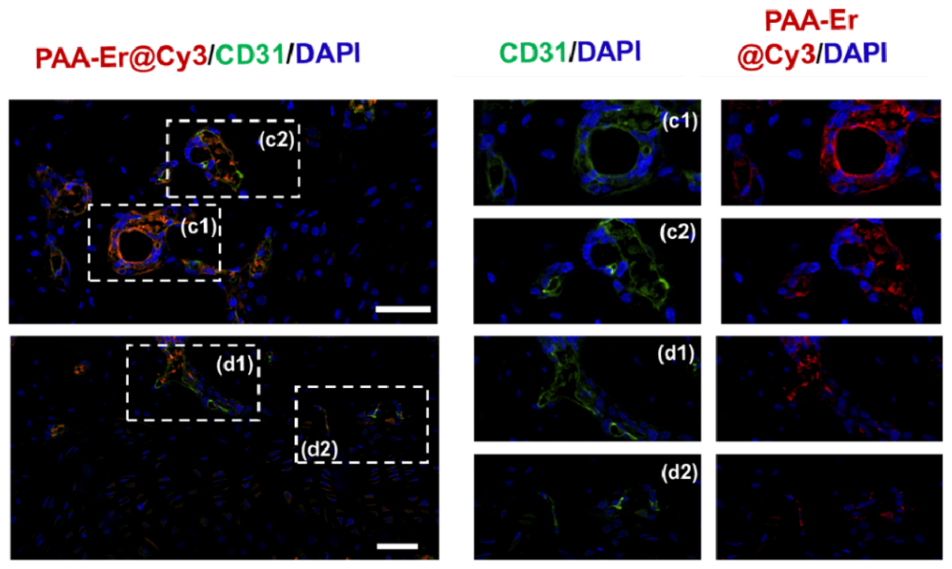	Endothelial cells of sinusoidal vessels
Chen, et al.[1] https://dx.doi.org/ 10.1021/acs.nanolett.0c04543	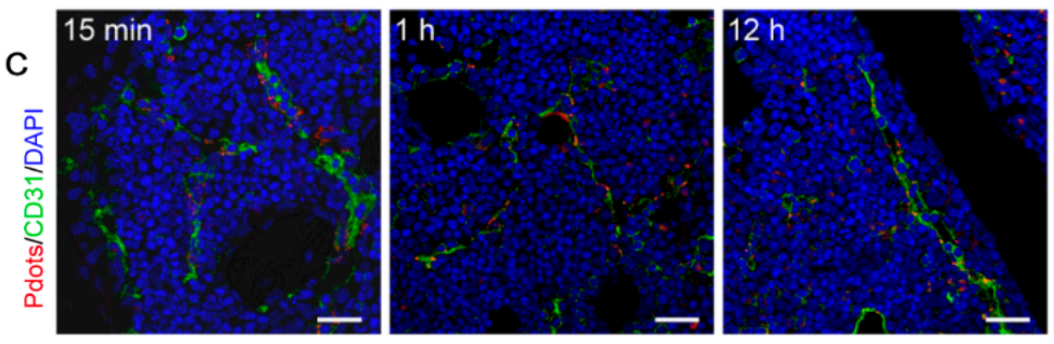	Endothelial cells of sinusoidal vessels
Mi, et al.[4] https://doi.org/ 10.1038/s41467-023-42001-2	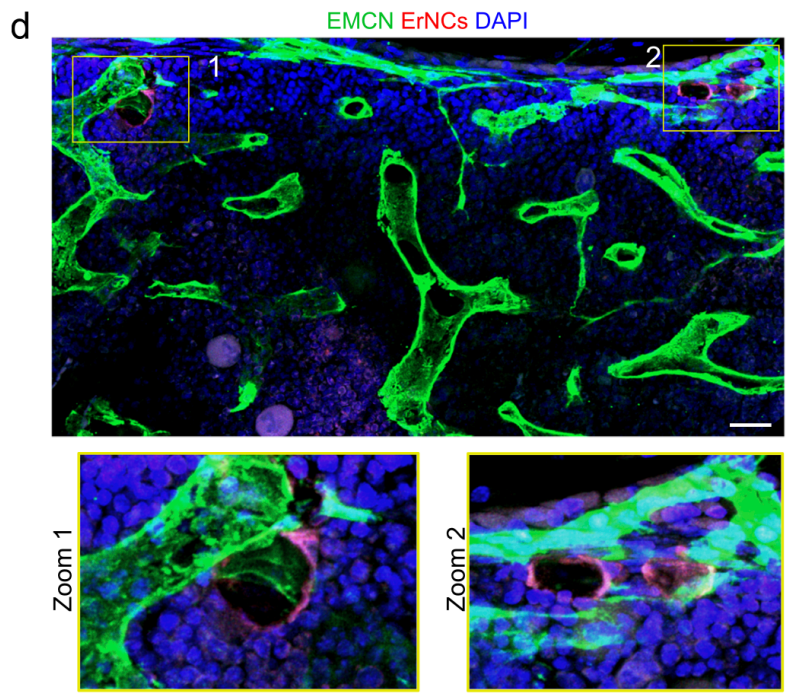	Perivascular macrophage in the bone marrow
Che, et al.[6] https://doi.org/ 10.1039/d0nr06261h	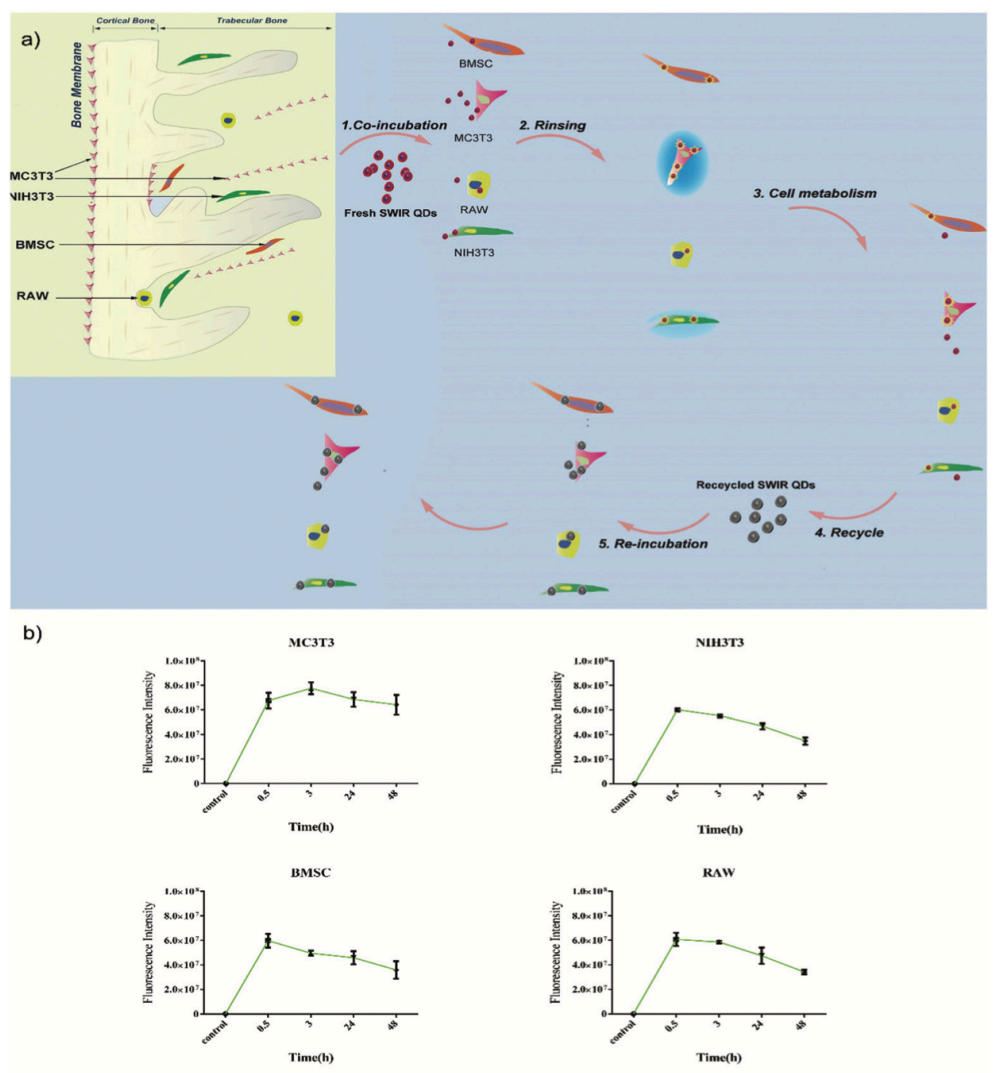	Osteoblasts

Similarly, in this study, SWIR signals was relevant to CD31-positive and α-SMA-positive area, which suggest the signals could be related to bone vessels. To avoid the misunderstanding, the text has been revised as "The results implied that the FL signals may be related to the bone vessels". Moreover, we appreciate the reviewer's constructive input and will incorporate investigation of αSMA-positive periosteal cells in future studies to further clarify the origin of the SWIR signal during bone healing.

Revision (Line 440): The results implied that the FL signals in bone may be related to the bone vessels[1,5].

3. Discussion – limitations:

A discussion of the limitations of the proposed imaging approach is missing and should be included. In particular:

a) Potential toxicity of the SWIR fluorophores must be discussed in relation to possible human applications. Figure S18 provides some relevant data, but it does not constitute a toxicological study. Have these fluorophores been tested or approved for human use?

b) What is the penetration depth limit of far-red/near-infrared light in human tissues? Figure 3g,h, and Video 7 are impressive but do not visualize ligaments, suggesting that deeper knee structures may not be detectable in humans due to the physical limits of light penetration. Please address these limitations explicitly in the Discussion.

Response: Thank you for these critical comments regarding the limitations of our study. Below, point-to-point response were provided.

Q3-1: Potential toxicity of the SWIR fluorophores

Response: First, the discussion of fluorophores has been added in the main text. In fact, to reduce the potential adverse reaction of the fluorophores, indocyanine green (ICG), an FDA-approved SWIR fluorophore, was also evaluated for joint imaging in our preliminary experiment. As a result, it showed insufficient and unstable fluorescence brightness within the joint cavity. The reason is that imaging performance of ICG heavily relies on binding to plasma proteins to form a stable complex, which prevents fluorescence quenching and prolongs circulation[8,9]. In contrast, synovial fluid has relatively low protein content[10] and leads to suboptimal imaging. Therefore, the previously validated probes with high biocompatibility and brightness in synovial fluid have been utilized in this study.

Second, the biosafety of this SWIR probes have been thoroughly investigated in prior studies for animal models[11] (Figure R2-4, Table R2), but they have not been tested or approved for human use. Therefore, future work should prioritize employing FDA-approved contrast agents. We would adopt FDA-approved, SWIR probes suitable for the joint environment as they become available.

The details of SWIR fluorophores biosafety were listed as below:

1) Long-term in vitro cell biosafety

Since the cell cycle influences cell fate transitions beyond proliferation, a cell cycle assay was conducted to evaluate the influence of fluorophores on MSCs (Mesenchymal Stem Cells)[11]. No significant changes in the cell cycle were observed in MSCs with different concentrations of PbS QDs (Figure R2A). Additionally, a colony-forming assay, regarded as a reliable indicator of pluripotent stem cell activity, demonstrated homogeneous colony size and morphology with no significant differences in colony numbers (Figure R2B), suggesting that the fluorophores were safe for cell self-renewal potential. To further assess the influence on differentiation capacity, adipogenic and osteogenic differentiation assays were performed, revealing successful differentiation into osteoblast and adipocyte lineages at PbS QDs concentrations up to 30 μg/mL. Similar intracellular lipid-rich vacuoles, alkaline phosphatase (ALP) activity, and calcium deposits were observed across

different concentrations (Figure R2C). These results indicate that PbS QDs did not impair MSC multipotentiality and demonstrated high long-term biosafety.

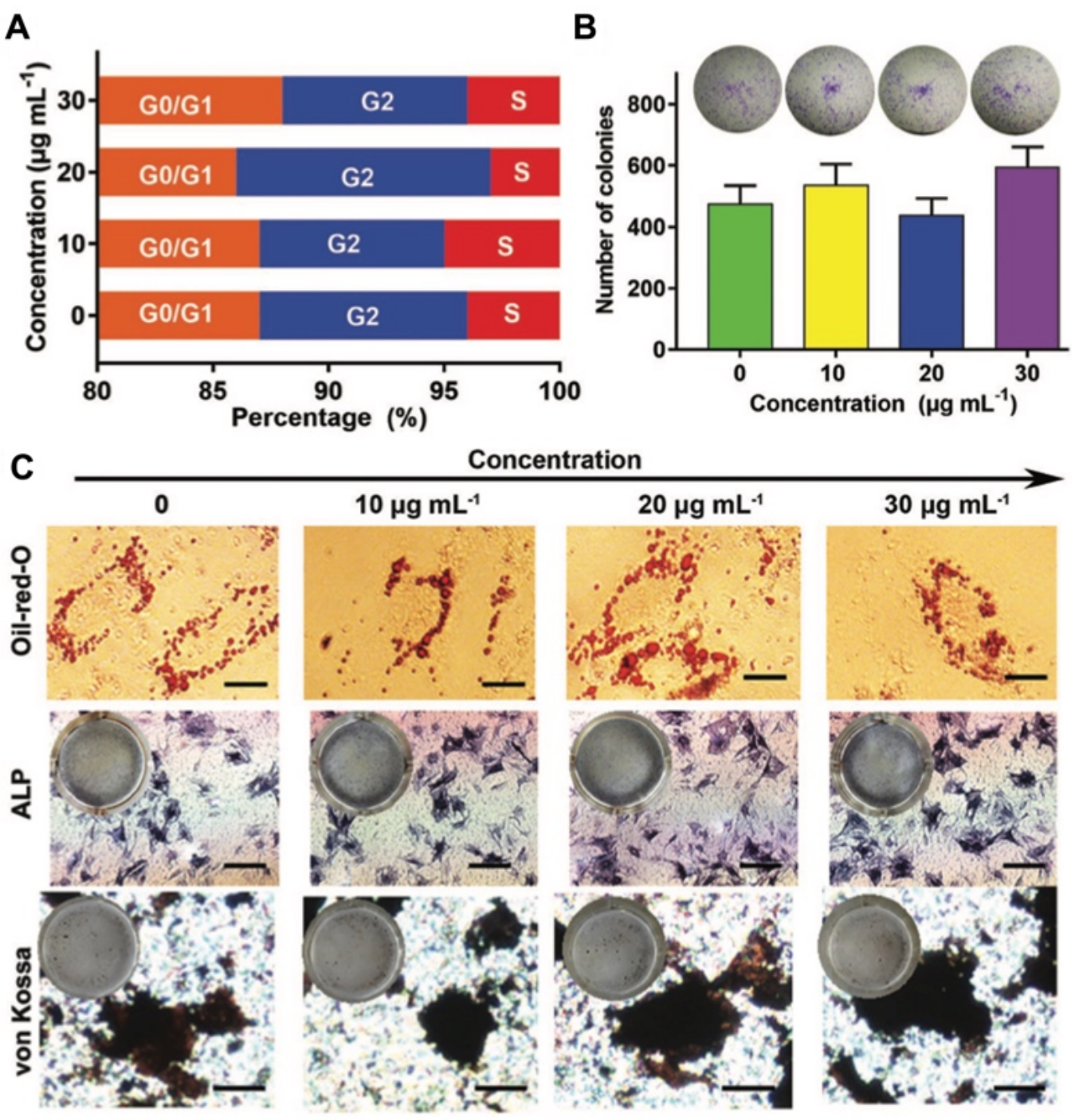

Figure R2. Interactions of different concentrations of Ribonuclease-A-encapsulated PbS QDs with MSCs. (A) Cell cycles detected by flow cytometry. (B) Comparisons of colony-formation efficiency in each group. (C) Adipogenic and osteogenic differentiation assays for labeled MSCs, scale bars represent 20 μm (oil-red O staining), 200 μm (ALP staining) and 50 μm (von Kossa staining).(Figure from [11])

Moreover, to assess MSC proliferation following incubation with PbS QDs, both live/dead staining and a CCK-8 assay were performed. Representative images confirmed that nearly all cells remained viable in each group (Figure R3A). No significant differences were observed in MSC morphology or viability (Figure R3B). Similarly, no statistical differences were detected between the 10 μg mL^{-1} group and other groups at the same time point ($p > 0.05$) (Figure R3C). These findings suggested that PbS QDs exhibit negligible cytotoxicity toward MSCs at concentrations ranging from 10 to 30 μg mL^{-1}.

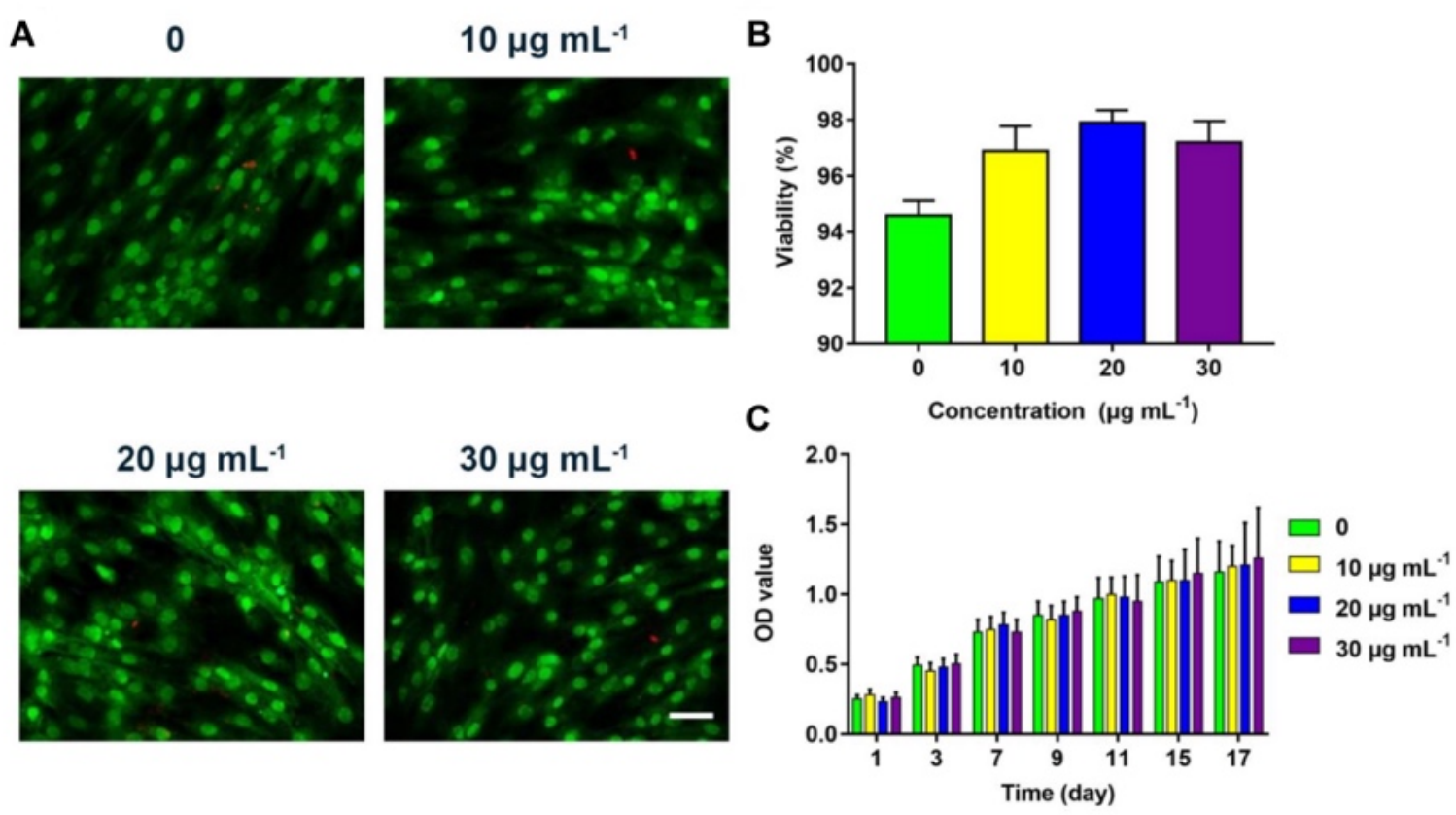

Figure R3. The effects of different concentrations of PbS QDs on cell proliferation and cell viability. (A). Live/dead cell staining for labeled MSCs (green fluorescence-live cells, red fluorescence-dead cells). Scale bars represent 50 μm. (B). Quantification analysis of cell viability of labeled MSCs. (C) Cell proliferation of labeled MSCs determined by CCK-8.(Figure from [11])

2) Long-term In vivo biosafety

In addition, long-term Pb ion level analysis of blood, feces, urine and major organs have been conducted in previous study[11] to evaluate the biosafety and clearance of PbS QDs fluorophores. Low concentration of Pb ion level was observed in blood, feces and urine in long-term analysis and the fluorophores did not deposit in major organs, suggesting high biosafety of our PbS QDs (Figure R4).

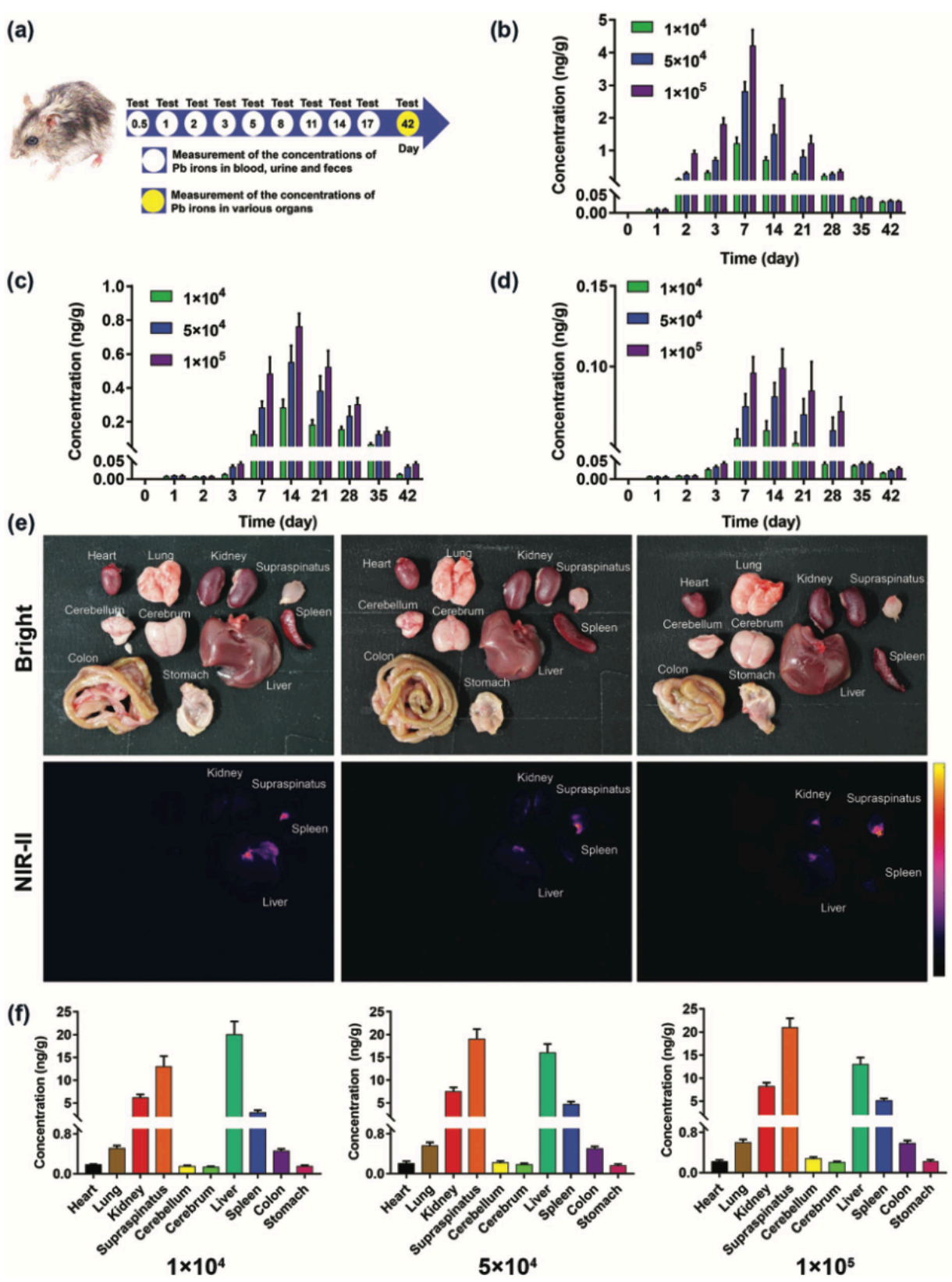

Figure R4. *In vivo* quantitative analysis of the distribution and clearance processes. A) Schematic illustration of the study protocol of investigating the clearance of Pb ions. B) Determination of the concentration of Pb ions in whole blood. C) Determination of the concentration of Pb ions s in feces. D) Determination of the concentration of Pb ions in urine. E) Bright field and NIR-II fluorescence images of various organs collected from the mice at 42 days after post-injection. F) Determination of the concentration of Pb ions in major organs. (Figure from [11])

3) Organ function biosafety

Moreover, the indicators of organ function after repeated dose administration (same as SWIR bone imaging in the study) were also measured and demonstrated in the Table R2, which showed all parameters were in range of normal value, indicating the low biotoxicity of QDs in current study.

Table R2 Indicators for liver and kidney function

Indicators	Mean ± SD	Normal value	In normal region
Liver Function			
ALT	60.42 ± 22.97 U/L	10.06-96.47 U/L	Yes
AST	189.17 ± 50.22 U/L	36.31-235.48 U/L	Yes
GGT	2.41 ± 0.98 U/L	0-7.78 U/L	Yes
ALP	197.56 ± 46.28 U/L	22.52-474.35 U/L	Yes
D-BIL	5.10 ± 0.49 μmol/L	0.45-33.89 μmol/L	Yes
T-BIL	12.73 ± 1.71 μmol/L	6.09-53.06 μmol/L	Yes
TBA	1.60 ± 0.45 μmol/L	0-8.51 μmol/L	Yes
ALB	31.09 ± 0.53 g/L	21.22-39.15 g/L	Yes
Kidney Function			
BUN	11.92 ± 1.40 mg/dl	10.81-34.74 mg/dl	Yes
CR	22.32 ± 4.77 μmol/L	10.91-85.09 μmol/L	Yes

Q3-2: Penetration depth

The specific penetration depth limit of far-red/near-infrared light in human tissues has not been fully investigated. Table R3 summarizes the penetration depths of far-red and near-infrared light reported in the literature. In the air, longer wavelength achieve lower scattering and greater penetration depth according to the Mie scattering[12] and Rayleigh scattering[13]. In biological tissue, the penetration depth depends on both tissue scattering and absorption[14]. On this basis, near-infrared (longer wavelength) result in deeper penetration depth in human tissue than far-red according to the literature[15].

Table R3. Comparison between far-red and near infrared properties

	Wavelength	Penetration Depth
Far-red[15,16]	600-700 nm	0.28-3.14 mm (blood-mammary carcinoma)
Near infra-red I[15]	700-900 nm	0.51-4.23 mm (blood-mammary carcinoma)
Near infra-red II (SWIR)[17,18]	1000-1700 nm	5.0-10.0 mm (skin)

Furthermore, researchers have been actively working to enhance the imaging depth of the SWIR window (1000–1700 nm) through the development of advanced probes and imaging systems. On the one hand, probes were improved by increasing emission wavelength and brightness to allow the deeper tissue imaging[19,20]. On the other hand, imaging equipment was optimized to visualize the deeper tissue with high resolution[21-23]. Therefore, future improvements in probe design (e.g. probes with longer emission wavelengths) and imaging equipment (e.g. higher-power excitation, more sensitive detectors) would enable further enhancement of deep-tissue imaging performance in human body. Thank you again for these insightful and constructive comments, and they were incorporated in the discussion section in the revised manuscript.

Revision (Line 519): Future work in next-generation functional musculoskeletal imaging should include improvement of biocompatibility and penetration depth for further clinical application, by employing advanced probes and cutting-edge equipment.

Minor comments

1.• Line 153:

"As an essential factor influencing the progression of osteoarthritis and rheumatoid arthritis …"

Please provide a reference supporting this statement.

Response: Thank you for noting the missing citation. We have inserted a citation to the relevant literature in the sentence.

Revision (Line 153): As an essential factor influencing the progression of osteoarthritis and rheumatoid arthritis[24,25].

2.• Figure 1c:

The arrows appear misaligned and should be corrected.

Response: Thank you for pointing out the misalignment. The arrows have been corrected to point precisely to the intended structures.

Revision (Line 72): Following is the revised Figure 1c.

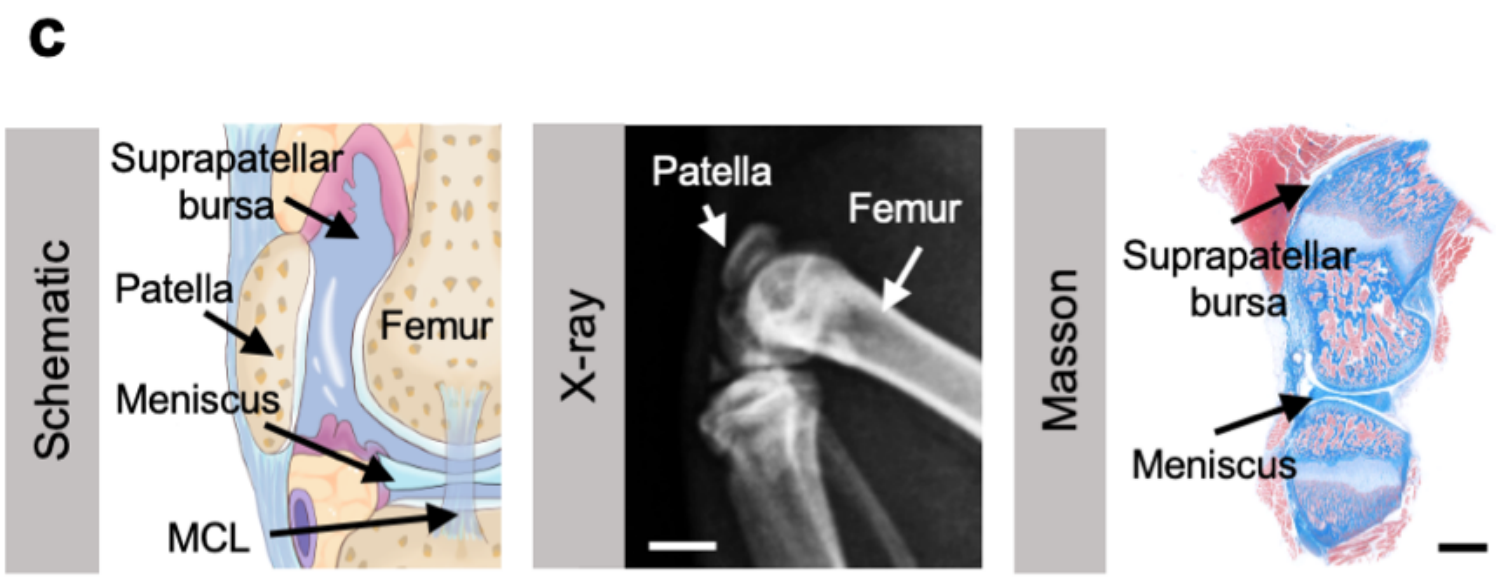

3.• Flexion/extension angle labeling:

The terminology and labeling are confusing. Although the explanation is reasonable, the graphical representation should be clearer. It would be helpful to annotate one of the relevant figures (e.g., Fig. 1j, p, or q) or to expand Fig. S6 with a schematic. As I understand it, flexion = 135 % corresponds to the most bent position, and flexion = 30 % to the most extended knee. If so, please verify that Figure 1p correctly shows that the MCL is longest when the knee is straight. This appears inconsistent with the text and with Figure S7C. Please clarify throughout.

Response: Thank you for highlighting the need for clarity in the graphical representation. The textual description and data in Fig. 1p are consistent with the reviewer's correct understanding. The apparent inconsistency may stem from the original figure's limited visual contrast when depicting the modest (~15%) length difference. To eliminate any ambiguity, we have revised the schematic in Fig. 1p to highlight the increased MCL length in the extended knee position, and also annotated the knee position in Figure 1j. The main text is also revised for readability.

Revision (Line 192): Analysis in Fig. 1p presented progressive MCL elongation during knee extension (from 135° to 30°), exhibiting a length increase of 15.11 ± 1.42 % beyond its length at full flexion (135°)

Line 72: Following is the revised Figure 1p and Figure 1j.

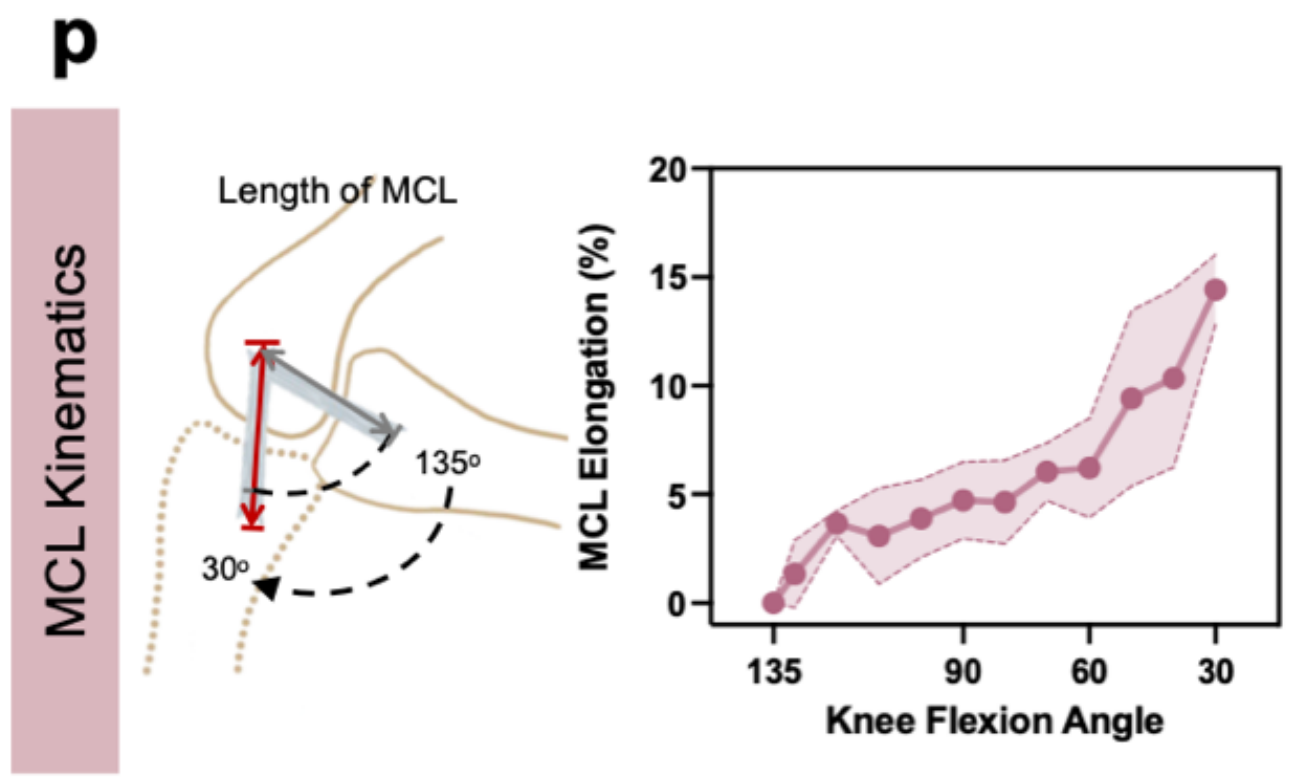

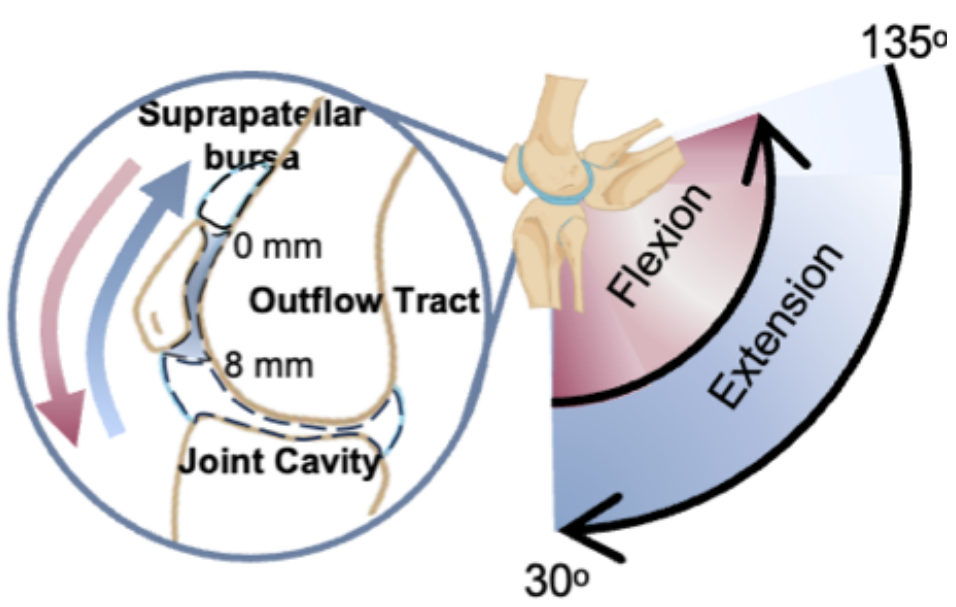

4.• Lines 313–315:

The explanation for the abnormal kinematics observed during patellar dislocation is unclear. Please elaborate.

The corresponding methods section also requires clarification—specifically, why time intervals of 0.03, 0.06, and 0.09 seconds were chosen, and whether the knee was flexed during these recordings.

Response: We thank the reviewer for highlighting these points for clarification. Below we provide detailed responses to each concern:

Q4-1. Explanation of abnormal kinematics during patellar dislocation:

We have expanded the explanation of the patellar dislocation in the main text to more clearly describe the kinematic abnormalities.

Q4-2. Justification for selected time intervals:

The time intervals of 0.03, 0.06, and 0.09 seconds correspond to the temporal resolution of our imaging system (0.03 seconds), where consecutive 4 frames were captured every 0.03 seconds. These 4 consecutive frames include the entire patellar dislocation process.

Q4-3. Knee position during recordings:

Recordings were performed with the knee in extended position.

We have incorporated these details into the main text and methods section and have revised the caption of Figure 3f to improve clarity.

Revision (Line 315): Critically, the system could capture the abnormal kinematics of patellar dislocation in real-time (Fig. 3f, Supplementary Video 5), from normal patellar location in the trochlear groove (frame no.1) to laterally dislocated patella out of trochlear groove (frame no. 4) in the time period of 0.09 s.

Line 577: 4) Rabbit patellar dislocation model: After the skin disinfection and incision, the patella was artificially dislocated by pushing patella to the lateral (dislocation, in 0.09s/4 frames) during joint imaging in the knee extension position.

Line 292: Following is the revised Figure 3f.

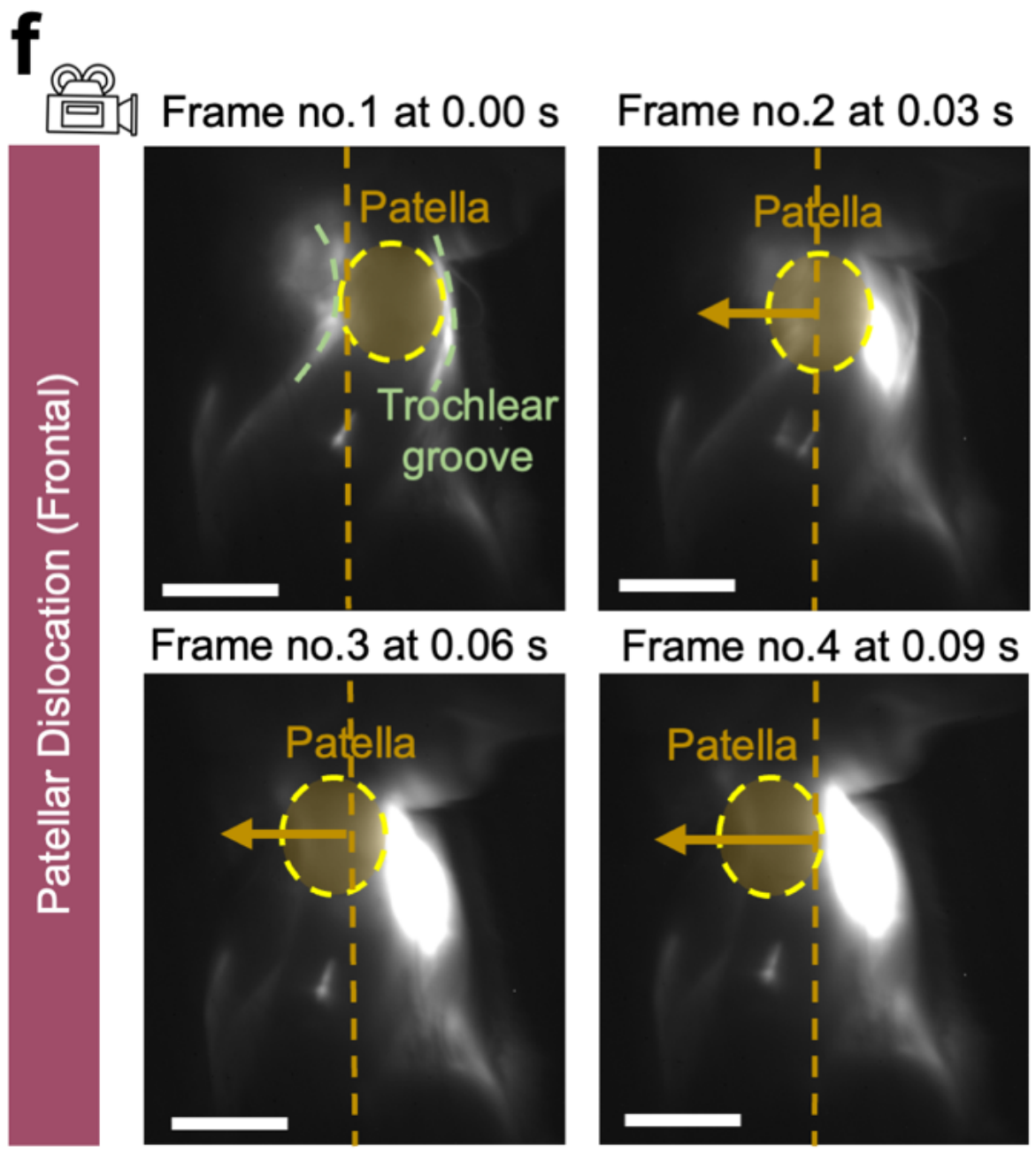

5• Human knee experiment:

This part is particularly impressive. Please clarify in the main text whether the skin was removed prior to imaging and how long after fluorophore injection imaging was performed.

Response: Thank you for your positive feedback on the human knee experiment and for raising these important methodological points. We have clarified both issues in the revised manuscript.

Revision (Line 320): SWIR joint imaging was utilized to visualize human knee extension over a 1.30-second period (Fig. 3g) immediately after articular injection of probes and skin dissection, which identified anatomic structure of the femur (Fig. 3h).

6• Figure 4b legend:

The statement “with tail-vein injection of SWIR fluorophores at 30 s post-injection” is incorrect. Please revise to indicate that imaging was performed 30 seconds after tail-vein injection, not that the injection occurred 30 seconds post-injection.

Response: Thank you for pointing out the confusion. We have corrected the sentence to clarify that imaging was performed 30 seconds after the tail-vein injection of SWIR fluorophores.

Revision (Line 346): **b,** SWIR images of vessels including femoral artery and branch artery at 1, 3, 7, 14, 28 d post-surgery with 30 s after tail vein injection of SWIR fluorophores.

7• Lines 373–374:

“Therefore, SWIR vessel imaging enabled the longitudinal monitoring of spatiotemporal vessel remodeling and the identification of the critical time point.”

Measuring diameters of two large arteries does not support a claim of vessel remodeling. Please rephrase more conservatively.

Response: We appreciate the reviewer’s important point. In response, we have revised the statement to remove the claim of general "vessel remodeling" and now focus more conservatively on the observed changes in diameter that our measurements directly support.

Revision (Line 378): Therefore, SWIR vessel imaging enabled the longitudinal monitoring of spatiotemporal changes in vessel diameter and the identification of the critical time point.

8• Lines 390–392:

“3 d – 7 d post-surgery was identified as a critical phase, coordinating with the temporal sequence of vessel remodeling.”

The data supporting this statement are unclear. Please clarify the basis for identifying this specific time window as a “critical phase.”

Response: Thank you for pointing out the need for clarification regarding the basis for identifying the critical phase. The conclusion on Lines 390-392 has been revised to reference supporting data.

Revision (Line 395): In summary, SWIR bone imaging enables monitoring of precise spatiotemporal patterns in bone healing, with the 3-7 d postoperative period identified as a critical phase where statistically significant changes first emerge, paralleling the timeline of vascular changes.

9• Lines 553–554:

“Human: The cadaveric body is the donation to the institution, and the study protocol was approved by the committee.”

Please specify the full name of the approving ethics or institutional review committee.

Response: We acknowledge the need for this detail. To comply with the journal’s double-blind review process, the information has been temporarily anonymized in the current manuscript. We will provide the complete name of the approving institutional review committee in the final version upon acceptance and publication according to the policy of Nature Portfolio.

Reviewer #2 (Remarks to the Author): 6
General Comments: This manuscript presents a SWIR imaging method for assessment of joint motion and functional diagnosis of musculoskeletal diseases. As one non-invasive, deep penetration and in vivo modality, the advances of SWIR imaging have been witnessed in the past decade, this topic is definitely important and could have impact on living tissue imaging, especially for skeletons deep inside the body. The highlight of this work is using SWIR imaging for the motion kinematics study, however, to my understanding, a state of free movement is necessary instead of anesthesia, as the muscle, tendon or bones do not exert force under anesthesia, this study ignored the joint motion during the free movement. Meanwhile, I am a bit confused on the knee joint images under different flexion angles, such a lot of results give no obvious differences.
The study goes on to report the in vivo imaging on the healing of tibial defect for 28 days, which proves the advantages of the SWIR imaging for long-term application. In general, the manuscript is interesting, but lacks convincing results and a high degree of novelty, the experimental results on musculoskeletal system should be published on a more specialized journal.
Response: We appreciate the reviewer's thoughtful critique, which has allowed us to better articulate the significance and applicability of our findings. Below, point-to-point response were provided.

Q0-1. a state of free movement is necessary instead of anesthesia, as the muscle, tendon or bones do not exert force under anesthesia, this study ignored the joint motion during the free movement
Response: Thank you for raising this important point. Briefly, the imaging protocol was designed in accordance with established clinical procedures. In clinical diagnostic process of sports medicine and orthopedic, passive movement without muscle guarding was required during physical examination[26]. For example, assessment of joint range of motion and specific tests (e.g. Lachman test for anterior cruciate ligament injury, Valgus stress test for medial collateral ligament injury, McMurray test for meniscus injury) were recommended to be performed during passive movement.

Moreover, examination under anesthesia (EUA) is regarded as the gold standard for assessing joint stability (e.g. pivot shift test, patellar dislocation test under anesthesia) [27,28]. It could exclude muscle guarding caused by pain. Therefore, our approach under anesthesia simulates this clinical examination to achieve the accurate joint functional evaluation.

Moving forward, the combination value of analysis under anesthesia and free movement was highly recognized[29,30]. Therefore, in the future study, we are planning to expand the imaging field of view and integrate motion-tracking technologies to explore joint dynamics during voluntary activity.

Q0-2. I am a bit confused on the knee joint images under different flexion angles, such a lot of results give no obvious differences
Response: Thank you for your comment. Briefly, this approach was designed in accordance with standard clinical procedures, and imaging under different flexion angles enables both visual observations and quantitative measurements.

First, examination in different angles was usually performed in physical examination and also in existing literature[26,31]. It was because the musculoskeletal injury could only be detected during motion, which is a continuous dynamic process. While consecutive frames may appear visually similar, differences become increasingly pronounced over longer time intervals.

Second, in this study, kinematics of key structures such as synovial fluid and patellar tracking exhibit distinct variations across different flexion angles (Figures 1h, 2c). These observed differences in multiple images offer direct visual evidence of structural and positional changes during motion, thereby being the essential prerequisite of the functional analysis.

Moreover, even when these differences are not visually perceptible, subsequent quantitative analysis is able to detect subtle variations in kinematic parameters. The quantitative analysis is a standard imaging analysis procedure in other imaging modalities[32]. This analytical approach enables the identification of minor yet functionally significant changes, thereby validating the precision of the imaging method for functional assessment. For example, patellar maltracking could be detected through quantitative analysis both in literature[33] and this study. In summary, the presentation of different images across flexion angles serves not only to illustrate visually apparent changes but also to provide the quantitative data for rigorous functional analysis.

Q0-3. lacks convincing results and a high degree of novelty, the experimental results on musculoskeletal system should be published on a more specialized journal

Thank you for your valuable feedback regarding the novelty and journal fit of our work. The innovative contributions and broader relevance of our study were clarified as follows:

1) **Novelty:** The previous work published in Nature Communications (2023,14:6287)[4] and others[1-3,5-7,34] has demonstrated impressive NIR-II skeletal and joint imaging. Based on these previous studies, our work presents the first dynamic functional assessment of musculoskeletal kinematics. This approach moves beyond structural to functional evaluation by providing real-time, dynamic, and quantitative kinematic data, being a significant advance with high innovative value.
2) **Scope:** As mentioned above, study regarding SWIR bone imaging has been published in Nature Communication (2023,14:6287)[4], demonstrating that the bone imaging falls within the scope of this journal. Our work, which focuses on a related area, similarly warrants consideration. Besides, according to the Aims & Scope of Nature Communication, it is a "multidisciplinary journal dedicated to publishing high-quality research in all areas of the biological, health, physical, chemical, Earth, social, mathematical, applied, and engineering sciences.". This study demonstrates for the viability and importance of optical imaging for diagnosis of musculoskeletal disease, thereby appealing to a multidisciplinary audience in biomedical engineering, health and biological fields. Therefore, this work is in the scope of journal and well-suited for a broad-audience, high-impact journal such as *Nature Communications*, where it can engage researchers and clinicians across multidiscipline.

I have the following comments/questions:

Q1. Fig 1f shows the SWIR fluorophores could be excreted out in 7 days after the intra-articular

injection, and the SWIR emission vanished in 2 hours. What is the metabolic pathway of the SWIR fluorophores? How is the excretability of fluorophores achieved?

Response: Thank you for your questions regarding the metabolic pathway and excretability of the SWIR fluorophores following intra-articular injection. According to the references and previous study, the SWIR fluorophores metabolize through circulatory system including lymphatic drainage and blood.

According to the reference, the synovial fluid within the joint cavity is part of the body's interstitial fluid[35]. Its primary clearance pathway is through lymphatic drainage into the venous circulation, after which it enters the systemic blood[36]. Intra-articularly injected drugs including viscosupplements were also reported to be metabolized through blood[37].

Similarly, in previous study on intra-articularly injected imaging probes[11], the probes can be detected in the blood and urine by component analysis (Figure R5). This confirms that the probe does enter the systemic circulation and is subsequently excreted via the urine.

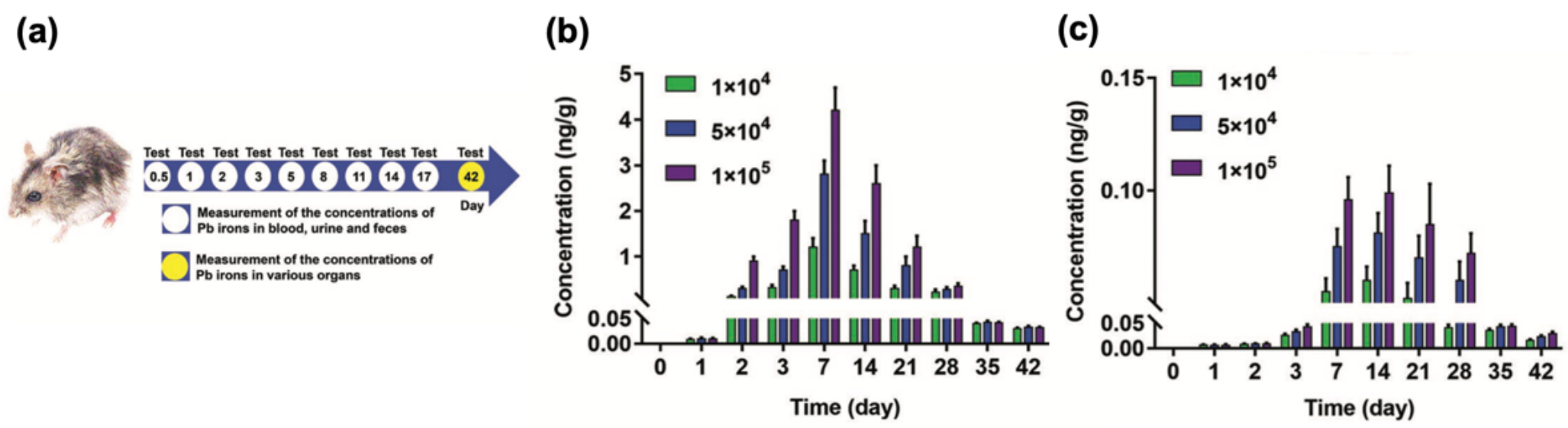

Figure R5. Quantitative analysis of the distribution and clearance in vivo. a) Schematic illustration of the study protocol of investigating the clearance of Pb ions. b) Determination of the concentration of Pb ions in whole blood. c) Determination of the concentration of Pb ions in urine. (Figures from [11])

Hope this clarifies the proposed metabolic and excretory route of the SWIR fluorophores used in this study. Thank you for raising this point.

Q2. The manuscript provides few information on the SWIR fluorophores, as the cited reference synthesized SWIR fluorophores for detecting bacteria, is there any difference between them?

Response: Thank you for your comment regarding the information on the SWIR fluorophores used in this study. The focus of our work was on developing and applying an imaging method for functional kinematic assessment In fact, the previous publication[38] has provided detailed synthesis and characterization data for this material (Figure R6-R8, Tabel R4). Moreover, the SWIR fluorophores were prepared following the protocol described in the Control group of the cited reference and not for bacteria labeling. To avoid any potential confusion, we have now explicitly cited the original methodology reference in the main text. We hope this clarification adequately addresses your question.

The details of SWIR fluorophores characterization were as below:

① *"size and morphology"*

In previous study[38], transmission electron microscopy (TEM) has been done to provide measurements of the size and morphology of RNase-A@PbS QDs (Figure R6a). High-angle annular dark-field scanning transmission electron microscopy (HAADF-STEM) image shows RNase-A@PbS QDs as bright spots (Figure R6b). The inset shows the size histogram of hundred particles, exhibiting an average size of 4.35 ± 0.07 nm (Figure R3b). Figure R6c presents an AFM topographic image of the well-dispersed spherical RNase-A@PbS QDs with height profiles ranging from 2 to 8 nm (with the height < 2 nm removed to cut-off the background noise). The height histogram of RNase-A@PbS QDs shows at ~5.0 ± 0.6 nm (Figure R6d). Moreover, gel electrophoresis analysis disproves the coformation of dimeric and/or multimertic RNase-A as well as their possible contribution to the higher height profiles observed in the AFM image.

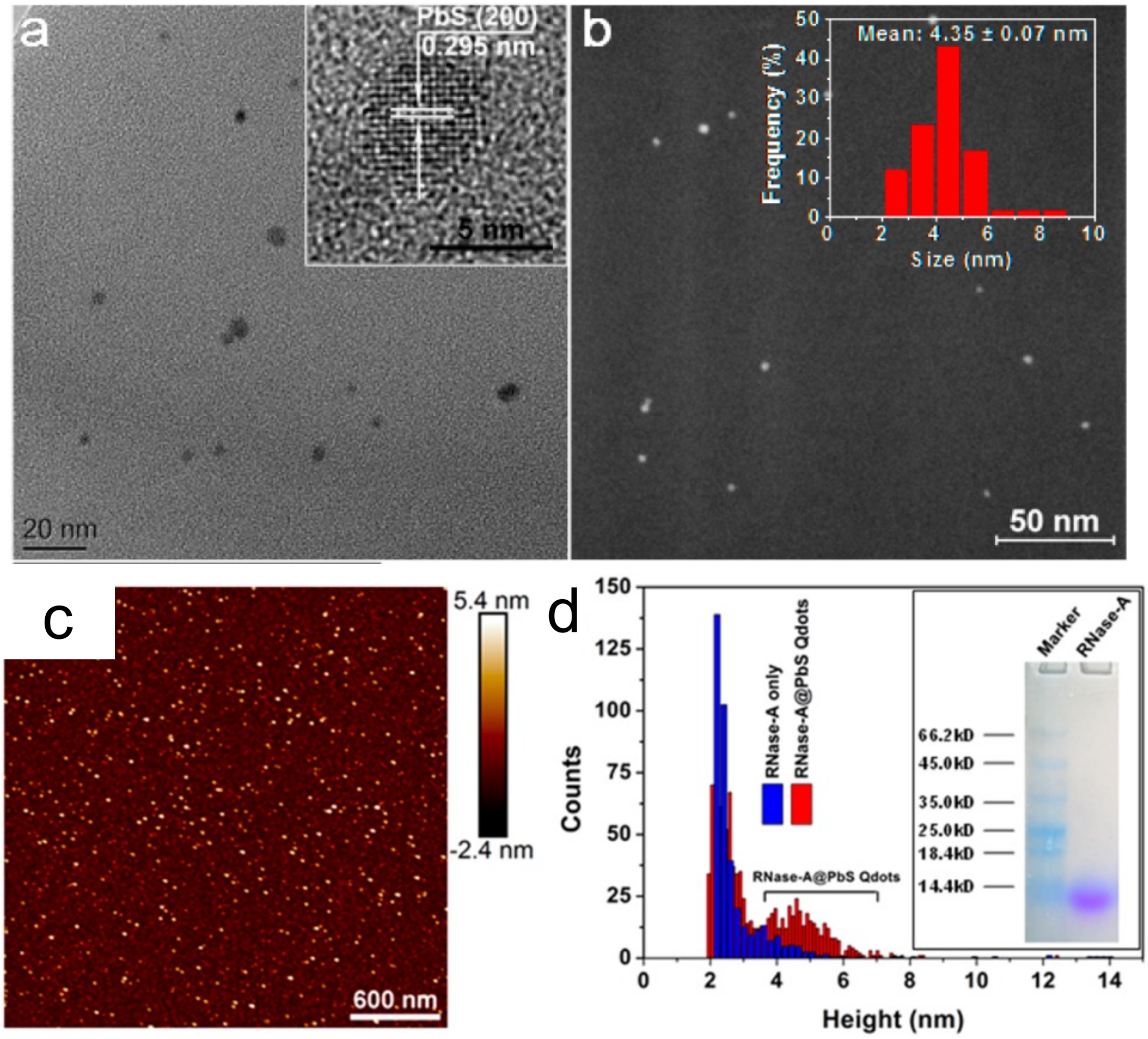

Figure R6. (a) TEM image of the as-synthesized RNase-A@PbS QDs. (inset is the HR-TEM image of singe RNase-A@PbS QDs) (b) High-angle annular dark-field scanning transmission electron microscopy (HAADF-STEM) image showing the QDs as bright spots. (c) Representative atomic force microscopy (AFM) topographic image of RNase-A@PbS QDs deposited on a flat mica with a dilution of 1:1000. (d) Corresponding height distribution histogram of RNase-A@PbS QDs and RNase-A only. Inset shows gel electrophoresis analysis of molecular weight marker (left lane) and pure RNase-A (right lane) which has undergone the identical process for the Qdot synthesis. (Figures from [38])

② *"zeta potential measurement"*

As shown in previous study[38], the RNase-A@PbS QD is negatively charged with zeta potential (ZP) of -2.5, -7.0 and -0.24 mV when placed in PBS, FBS and DMEM, respectively (Table R4).

Table R4. Zeta potentials (ZPs) of RNase-A@PbS QD in three different media over a two-week storage. (Table from [38])

Storage \ Medium	PBS (pH 7.2)	FBS (pH 7.1)	DMEM (pH 7.6)
0 day	-2.5 ± 27	-7.0 ± 13.5	-0.24 ± 11
14 days	-5.4 ± 10.4	-2.4 ± 14.1	-5.0 ± 9.4

③ *"Photoluminescence (PL) spectra"*

Figure R7 shows fluorescence spectra of the as-prepared RNase-A@PbS QDs. The QDs prepared display a Gaussian-shaped emission peaking at 1308 nm (Figure R4).

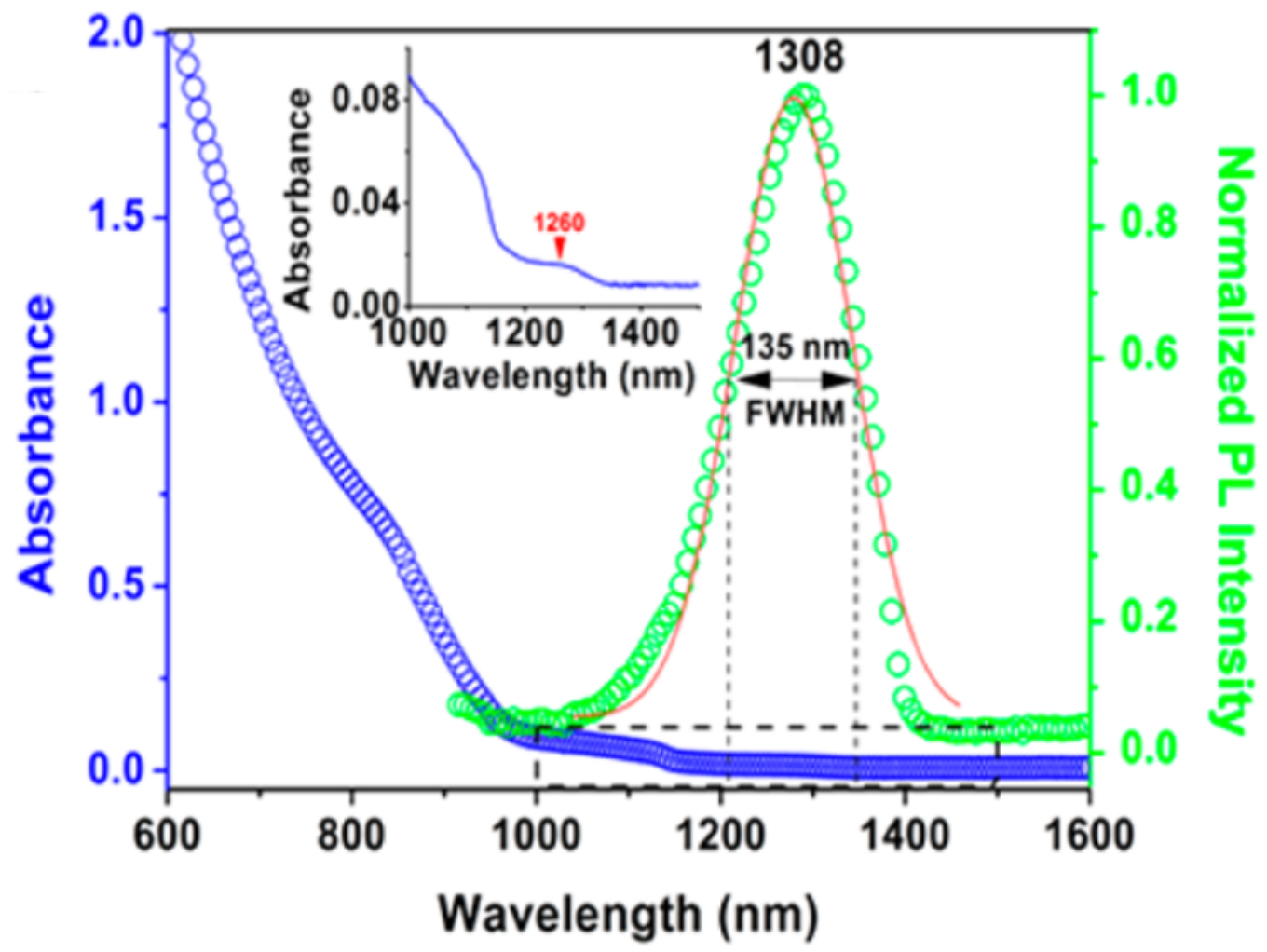

Figure R7. UV/vis/NIR absorption and PL spectra of the QD with an emission maximum peaking at 1308 nm. The PL spectrum is fitted to a Gaussian function. (Figures from [38])

④ *"tissue penetration"*

The penetration of RNase-A@PbS QDs in tissue has been confirmed. The measurement of fluorescent intensity indicates that the maximum muscle penetration for RNase-A@PbS QDs is about 1.5 cm (Figure R8), which suggests prepared RNase-A@PbS QDs were suitable for *in vivo* imaging.

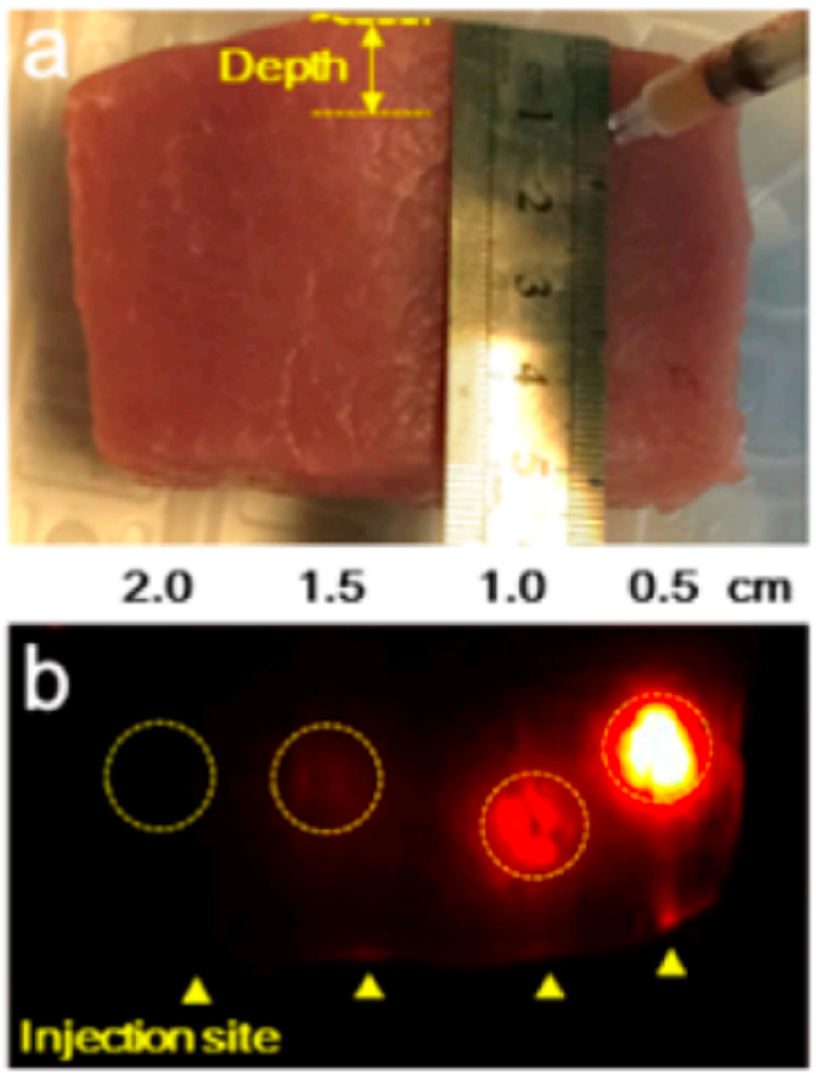

Figure R8. (a) Front-view optical image of a pork muscle tissue with 50 μL-130 nM RNase-A@PbS Qdots being injected at different depths of 0.5, 1, 1.5, and 2 cm. (b) Top-view fluorescence image of the tissue. (Figures from [38])

Revision (Line 547):

Highly fluorescent protein-encapsulated lead sulfide QDs were synthesized according to reported method[38,39]

Q3. After the intra articular injection, how do the fluorophores label the tissues?

Response: Thank you for your question. The SWIR fluorophores do not directly label the tissues. The imaging strategy of SWIR was similar to that of magnetic resonance imaging (MRI). MRI assesses proton density, with radiologists identifying anatomical structures by characterizing tissue morphology and localization based on proton density. Besides, in MRI arthrography[40], it also identifies the tissues with anatomical morphology and localization after intra-articular injection of probes.

Similarly, in our SWIR imaging, the structures are identified mainly based on anatomical morphology and localization. Briefly, SWIR fluorophores diffuse and mix uniformly with the synovial fluid after intra-articular injection. Dense, light-impermeable tissues such as the femur, medial collateral ligament (MCL), and meniscus[26] appear as signal voids against the fluorescent background. Tissue identification is then achieved by characterization of anatomical morphology and localization.

To further validate the observed signal voids correspond to the intended anatomical structures, layer-by-layer anatomical dissection was performed in parallel with imaging, as shown in Supplementary Fig. 1. This direct visual comparison (gold standard[41]) confirms that the structures resolved in the SWIR images accurately represent the physical anatomy of the knee joint. Hope this explanation clarifies the contrast mechanism and the approach used for tissue identification in our study.

Q4. Do the SWIR fluorophores have any targeting ability to the bones or other tissues? If not, how to distinguish different tissues in the joint? Like the ligament, meniscus, and patella.

Response: Thank you for raising these important questions regarding the targeting ability of the SWIR fluorophores for distinguishing different joint tissues. The point-to-point responses were shown below:

Q4-1: Targeting ability to bone or other tissues

Response: Upon intravenous injection, SWIR bone imaging could be achieved[1-7]. However, the mechanism and whether it has targeting ability have not been fully understood. Briefly, the probe is potentially taken up by bone marrow sinusoidal endothelial cells, macrophages, or osteoblasts to achieve SWIR bone imaging. Please see details at previous response to Reviewer 1, Question 2.

Q4-2: Distinguishing ligaments, meniscus, and patella

Response: Briefly, similar to MRI, different structures within the joint are distinguished primarily based on their anatomical morphology and spatial location, which is supported by macroscopy results. Please find detailed responses in our reply to Reviewer 2, Question 3.

Q5. In Fig 2i, how to manipulate the angle of joint flexion at 30°, 60°, 90°, and 135°? Is the approach precise enough to assess the overall MCL spatial displacement? As these assessments depend on the flexion angles very much.

Response: Thank you for your question regarding the measurement of joint flexion angles and its reliability. In the study, the angles were measured according to the standard clinical procedure of knee flexion angle assessment, which measures the angle between the longitudinal axes of the femur and the tibia[26]. Then the data were independently verified by three experienced surgeons in our hospital to ensure accuracy and reproducibility. After that, based on literature[31,42], knee flexion angle of 30°, 60°, 90°, and 135° were selected for further analysis, since they are typical knee flexion angles generally utilized in clinical examination (e.g. Lachman test performed specifically at 30° of flexion[28]). Thank you again for this constructive suggestion. To improve the above methodological transparency of the study, a detailed description of this measurement has been added to the Methods section of the manuscript.

Revision (Line 677):

The knee flexion angles were determined through frame analysis of motion videos by measuring the femoral-tibial angle and verified independently by three experienced surgeons.

6. SWIR skeletal imaging is a rapidly emerging field, some representative papers in this field should be properly cited, such as Nature Communications 2025, 16, 4911 (SWIR knee joint imaging); Nature Communications 2023, 14, 6287 (bone defect and disease imaging); Nano Lett. 2021, 21, 1, 798–805 (NIR-II skeleton imaging by polymer nanoparticles); Nano Lett. 2019, 19, 5, 2985–2992.

Response: Thank you for your valuable suggestions on adding representative references in the field of SWIR skeletal imaging. Recommended papers have now been cited in the main text accordingly.

Revision (Line 54): Besides, it has shown unparalleled contrast and resolution for *in vivo* visualization of deep-seated anatomic structures including joints and bones[1-4,43]

Line 442: The results implied that the FL signals in bone may be related to the bone vessels[1,5].

Reviewer #3 (Remarks to the Author): 11

This manuscript presents a technically ambitious and conceptually novel imaging platform using live short-wave infrared (SWIR) fluorescence imaging to achieve real-time, motion-robust, multi-component functional assessment of the musculoskeletal system. The central premise is that conventional imaging modalities, including X-ray, CT, MRI, and ultrasound, are limited by static acquisition, poor temporal resolution, or the inability to capture multiple interacting functional components during locomotion. The authors propose live SWIR imaging as a next-generation diagnostic paradigm capable of simultaneously visualizing deep anatomic structures, motion kinematics, synovial fluid dynamics, vascular remodeling, and bone biological activity in vivo.

The work is structured as a progressive demonstration of capability. The authors first establish that SWIR joint imaging, following intra-articular injection of SWIR fluorophores, can non-invasively visualize deep knee joint structures in rats without the need for dissection, achieving millisecond-scale temporal resolution. They then exploit this capability to quantify normal motion kinematics during knee flexion, including synovial fluid circulation, ligament elongation and angular displacement, and patellar tracking. These measurements are validated against macroscopic observations and shown to capture dynamic behaviors that are inaccessible to static MRI or CT.

The platform is then applied to functional diagnosis in injury models, including isolated patellar tendon injury and combined tendon–muscle injury. Using patellar tracking slopes and directional displacement metrics, the authors demonstrate that SWIR imaging can discriminate not only between the presence of injury but also between injury types and severities based on kinematic signatures rather than anatomy alone. Importantly, they further show that ligament functional impairment can be detected in a bone injury model even when the ligament remains anatomically intact, underscoring the sensitivity of the approach to early functional pathology.

The manuscript then expands its translation to rabbit knees and a human cadaveric knee, demonstrating that SWIR imaging can visualize joint structures through intact skin and capture abnormal kinematics, such as patellar dislocation and mechanical locking during extension. Finally, the authors extend the platform beyond joints to longitudinal monitoring of vascular remodeling and bone healing in a mouse tibial defect model. By exploiting distinct post-injection imaging windows, they sequentially image vessels and bone, revealing a temporal decoupling between angiogenesis and bone regeneration. This demonstrates that the SWIR bone signal correlates more strongly with vascular biological activity than with static bone volume measured by micro-CT. The study culminates in a holistic, sequential imaging workflow integrating vessel remodeling, bone biological activity, and joint kinematics in the same animal.

The strengths of this work are substantial. Conceptually, it reframes musculoskeletal imaging around function rather than static structure, convincingly arguing that many clinically relevant pathologies manifest primarily during motion. Technically, the imaging platform achieves an unusual combination of penetration depth, temporal resolution, and multi-component sensitivity that is not available with existing modalities. The breadth of experimental validation is impressive, encompassing multiple species, various injury models, and diverse functional domains. The quantitative analyses of synovial fluid dynamics, ligament elongation, and patellar tracking are

particularly compelling, as is the demonstration that SWIR bone signal reflects biological activity rather than simply mineralized tissue. The figures are generally clear, information-dense, and well aligned with the narrative, and the study is ambitious in scope while remaining internally coherent.

Response: Thank you for your thoughtful comments and positive feedback on our work. Below are our point-by-point responses.

Despite these strengths, the manuscript has several significant limitations that need to be addressed.

1.The most significant concern is the small sample size in most experiments, with many key conclusions based on n = 3 animals per group. While understandable, given the technical demands, this limits statistical power and raises concerns about robustness, particularly for claims regarding diagnostic sensitivity, injury discrimination, and temporal "critical phases" of healing. Variability is not always fully reported, and some statistical comparisons appear underpowered relative to the strength of the conclusions drawn.

Response: We appreciate the reviewer's attention to the sample size used in our study. A sample size of n = 3 per group has been widely adopted in many published studies of preclinical imaging and biomechanics research, particularly for longitudinal in vivo experiments[29,44-47]. Therefore, we included three independent animals for per group based on literature.

To further enhance the reliability of our findings, the sample size for key experiments was increased from n = 3 to n = 6 per group. The experiments were repeated, and the figures were revised accordingly. Notably, the results from these expanded experiments consistently align with the original trends, thereby confirming the robustness of initial conclusions.

Revision:

Line 102: Data were presented as the Mean ± SD of six independent rats (n=6)

Line 245: Data were presented as the Mean ± SD of six independent rats (n=6).

Line 307: Six independent rabbits (n=6)

Line 562: six independent animals were included

Line 681: Data from six independent animals were included in the data analysis.

Figures were revised correspondingly.

2.A second major limitation is the reliance on exogenous SWIR fluorophores, including lead sulfide quantum dots, which raises substantial translational and regulatory concerns. While clearance and short-term biosafety are addressed, the manuscript does not sufficiently discuss long-term toxicity, repeat-dose feasibility, or clinical alternatives. This omission weakens claims of near-term clinical applicability, particularly when the human data are limited to a single cadaveric specimen.

We sincerely thank the reviewer for raising these critical points. Below, we address the three main points raised from comments:

Q2-1: Regarding the long-term toxicity and repeat dose feasibility of quantum dots

Response: Actually, the long-term toxicity and repeat dose feasibility of this probe have been thoroughly investigated in previous studies. Please find detailed information in Reviewer 1 Question 3-1.

Q2-2: Clinical Alternatives

Response: Briefly, FDA-approved agents with SWIR emissions which is suitable for joint imaging could serve as direct substitutes. Please find detailed information in Reviewer 1 Question 3-1. To clarify, the Discussion section was expanded to explicitly outline this possibility.

Revision (Line 519): Future work in next-generation functional musculoskeletal imaging should include improvement of biocompatibility and penetration depth for further clinical translation, by utilizing high-quality probes and high-performance equipment.

Q2-3: Single Cadaveric Specimen

Response: In this study, single cadaveric specimen was utilized because the available unfixed cadaveric specimen is limited. The formalin-fixed specimens become rigid and cannot replicate the necessary motion in this study[48,49]. In comparison, unfixed cadaveric specimens suitable for dynamic motion are exceptionally rare and precious resources. We are actively seeking opportunities to access additional suitable specimens and would perform further validation in the future.

Q3: Third, while the authors repeatedly emphasize "diagnostic" capability, the study stops short of rigorous diagnostic validation. There is no comparison against gold-standard clinical diagnostic criteria, no receiver-operating characteristic analysis, and no demonstration of sensitivity or specificity across heterogeneous disease states. The injury models are well-controlled but relatively artificial, and it remains unclear how the proposed metrics would generalize to spontaneous or degenerative human diseases.

Q3-1: Regarding the Lack of Comparison Against a Gold Standard

Response: Thank you to point out the absence of a direct comparison against a clinical diagnostic "gold standard". Currently, there is no "gold standard" for the dynamic, functional assessment of joints. The current clinical diagnosis relies on static anatomical imaging (CT/MRI, Supplementary Fig. 11) combined with functional physical examination[35]. The imaging focus on static analysis, and the physical examination is highly subjective and dependent on clinician experience. As a consequence, none of them could work as "gold standard"[50]. This is exactly the specific diagnostic gap that we aim to address with proposed imaging approach. Instead of comparing to "gold standard", SWIR-derived functional metrics were validated by comparing to the results obtained from macroscopic analysis (Supplementary Figs. 7-8)[51], providing direct and reliable validation of the results.

Q3-2: Regarding Generalization to Spontaneous or Degenerative Diseases

Response: The proposed metrics could be generally utilized in spontaneous or degenerative diseases in both literatures[34,52] and our ongoing study. For example, the proposed imaging methodology is already applicable to the detection of cartilage defect and osteoarthritis, a spontaneous, degenerative disease[34]. In our preliminary data regarding aged model, it already revealed significant differences in synovial fluid function, characterized by reduced fluid flow in aged vs. young joints (Figure R9). Therefore, the proposed metrics could be utilized in spontaneous

and degenerative model. Accordingly, the Discussion part was amended the to explain the need for functional imaging in degenerative joint diseases.

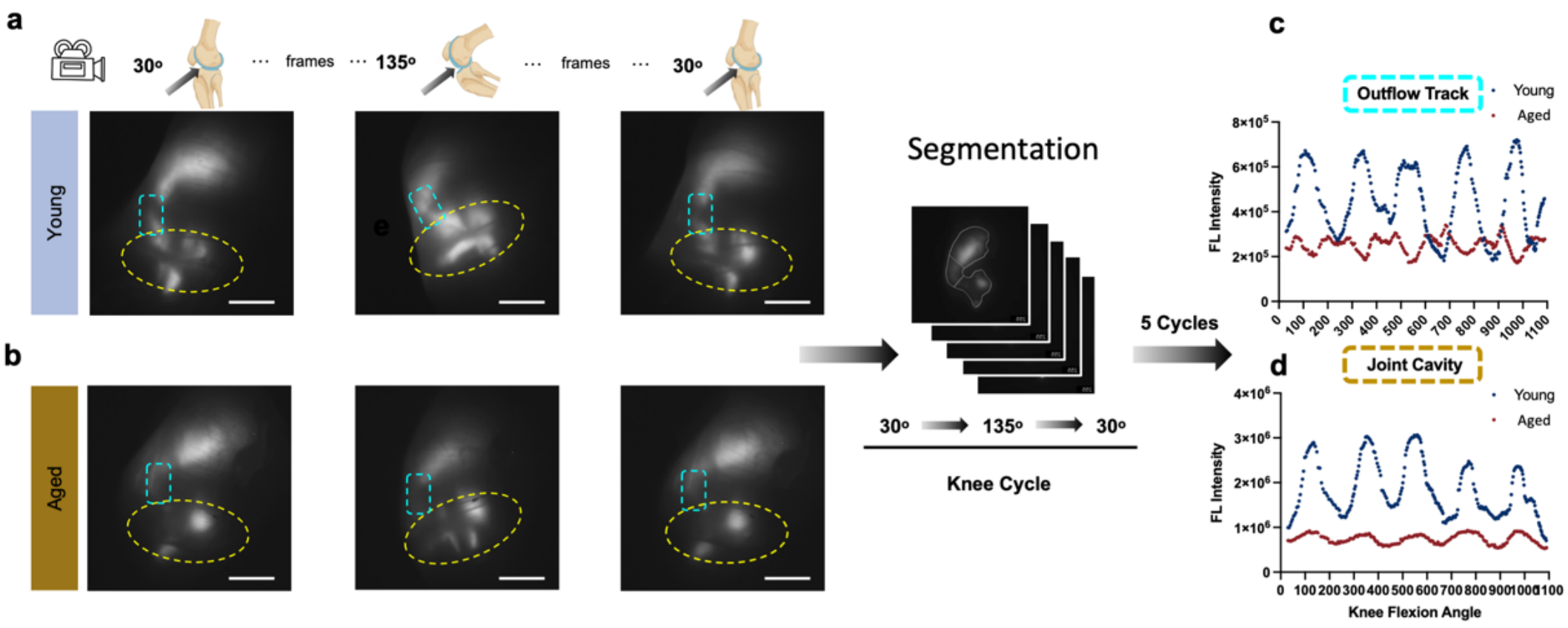

Figure R9. Differences in synovial fluid flow between young and aged rats. (a-b) SWIR images of synovial fluid flow in young (a) and aged (b) rats. (c-d) Quantitative analysis of synovial fluid flow pattern in outflow track (c) and joint cavity (d). Scale bar: 0.5 cm.

Revision (Line 504):

Further validation of synovial fluid kinematics through precision particle tracking and hydromechanical analysis and studies of other spontaneous disease models such as degenerative osteoarthritis would facilitate a more rigorous elucidation of its functional roles in knee joint biomechanics.

4.The manuscript also suffers from overextension in places. The scope is extremely broad, covering joints, ligaments, synovial fluid, vasculature, and bone healing. While each component is individually interesting, the cumulative effect is a very long and dense manuscript that risks obscuring its core contributions.

Response: Thank you for raising this important point. The broad scope of our study was not an overextension, rather was necessitated by the multi-component nature of the disease[53-55]. Accordingly, in the musculoskeletal disease progression, joints, ligaments, synovial fluid, vasculature, and bones are all included[35]. Omitting any one of them could result in an incomplete assessment of the system[56]. Importantly, these components were ultimately integrated into a single, unified imaging protocol at the end of the manuscript. It was done to demonstrate that holistic assessment is feasible and can offer a panoramic understanding of musculoskeletal diseases. To improve readability and clarify the logical flow from individual analysis to integrated imaging, the text regarding the sequential imaging section has been revised, ensuring the narrative better connects each component to the overarching aim of the study.

Revision (Line 461): Following the individual demonstration of SWIR imaging for key musculoskeletal components including bone, ligament, synovial fluid, and vasculature during locomotion, we performed its ultimate test of synchronous, multi-component imaging. This was achieved by sequential imaging of all above-mentioned assessments in the same individual mouse in motion-robust and multi-componential way.

5.The discussion occasionally overstates clinical implications without fully acknowledging the technological, biological, and regulatory gaps that remain.

Response: Thank you for this valuable feedback. The main text regarding clinical implications has been simplified to provide a more rigorous interpretation.

6.Additionally, the methods section is lengthy and technically complex and would benefit from clearer organization and schematic summarization to improve reproducibility.

Response: Thank you for your constructive feedback regarding the length and complexity of the Methods section. In response, we have thoroughly restructured the Methods section as follows:

1) Consolidation of Similar Methods: Descriptions of similar imaging procedures have been merged into coherent subsections to avoid redundancy and improve flow.
2) Addition of Clear Subheadings: The section now includes descriptive subheadings that logically align the related methodological steps, making it easier to follow the experimental workflow.

After these revisions, the Methods section now totals 2,256 words, which is within the journal's recommended limit of 3,000 words for this section. We appreciate your suggestion, which has helped us improve the overall readability and structure of the manuscript.

7.To improve the manuscript, the authors should temper their claims regarding diagnostic readiness and emphasize the work as a potentially powerful preclinical and translational platform rather than a near-clinical diagnostic tool.

Response: Thank you for this important suggestion. To temper the claims regarding diagnostic readiness, the text was carefully revised throughout to demonstrate preclinical and translational platform. The claims related to direct clinical application was revised across the Introduction, Results and Discussion ensuring that language reflects the current preclinical stage of the technology.

8.Expanding sample sizes for key experiments, or at the very least, providing stronger justification and power analysis, would substantially enhance confidence in the findings.

Response: We appreciate the reviewer's attention to the sample size used in the study. Briefly, the sample size of key experiments was expanded and the results were updated accordingly. Please see the detailed response and revision in Reviewer 3 Question 1.

9.A more explicit discussion of fluorophore safety, alternative contrast strategies, and regulatory pathways is essential.

Response: Thank you for the comments. For fluorophore safety and alternative contrast strategies, please find detailed responses in responses to Reviewer 3 Question 2-1 (fluorophore safety) and Reviewer 3 Question 2-2 (alternative contrast strategies). Briefly, previously published data have done the thorough biosafety analysis, and new FDA-approved SWIR contrasts would serve as alternatives. Regarding regulatory pathways, the novel contrast agents that was not previously approved require a full New Drug Application (NDA). It necessitates comprehensive preclinical toxicology studies, phase I-III clinical trials establishing safety and efficacy, and chemistry, manufacturing, and controls (CMC) documentation[57,58]. Correspondingly, they were added to the discussion parts in the revised manuscript.

Revision (Line 519): Future work in next-generation functional musculoskeletal imaging should include improvement of biocompatibility and penetration depth for further clinical translation, by utilizing high-quality probes and high-performance equipment.

10.The diagnostic claims would be more convincing if supported by formal performance metrics or blinded classification tasks.

Response: Please see the detailed response in Reviewer 3 Question 3-1. Briefly, absence of "gold standard" for functional assessment restricts to conduct the blinded classification tasks or demonstrate formal performance metrics.

11.Finally, tightening the narrative to focus more clearly on the unifying concept of motion-robust, multi-component functional imaging—rather than repeatedly re-demonstrating capability—would improve clarity and impact.

Response: Thank you for this insightful suggestion. In response, the text was thoroughly revised to streamline descriptions of technical feasibility and emphasizing the motion-robust, multi-component functional imaging framework.

References for point-to-point response letter

1 D. Chen *et al.* NIR-II Fluorescence Imaging Reveals Bone Marrow Retention of Small Polymer Nanoparticles. *Nano Lett* **21**, 798-805, doi:10.1021/acs.nanolett.0c04543 (2021).

2 S. He *et al.* High Affinity to Skeleton Rare Earth Doped Nanoparticles for Near-Infrared II Imaging. *Nano Lett* **19**, 2985-2992, doi:10.1021/acs.nanolett.9b00140 (2019).

3 D. Liu *et al.* Near-infrared II cyanine fluorophores with large stokes shift engineered by regulating respective absorption and emission. *Nat Commun* **16**, 4911, doi:10.1038/s41467-025-60241-2 (2025).

4 C. Mi *et al.* Bone disease imaging through the near-infrared-II window. *Nat Commun* **14**, 6287, doi:10.1038/s41467-023-42001-2 (2023).

5 Youbin Li *et al.* In Vivo High-Resolution Bioimaging of Bone Marrow and Fracture Diagnosis Using Lanthanide Nanoprobes with 1525 nm Emission. *Nano Letters* **22**, 2691-2701, doi:10.1021/acs.nanolett.1c04531 (2022).

6 Y. Che *et al.* In vivo live imaging of bone using shortwave infrared fluorescent quantum dots. *Nanoscale* **12**, 22022-22029, doi:10.1039/d0nr06261h (2020).

7 D. Li *et al.* Gold Nanoclusters for NIR-II Fluorescence Imaging of Bones. *Small* **16**, e2003851, doi:10.1002/smll.202003851 (2020).

8 S. Yoneya *et al.* Binding properties of indocyanine green in human blood. *Invest Ophthalmol Vis Sci* **39**, 1286-1290 (1998).

9 M. Hutteman *et al.* Optimization of near-infrared fluorescent sentinel lymph node mapping for vulvar cancer. *American Journal of Obstetrics and Gynecology* **206**, doi:10.1016/j.ajog.2011.07.039 (2012).

10 R. H. Shmerling *et al.* Synovial fluid tests. What should be ordered? *Jama* **264**, 1009-1014 (1990).

11 Y. Yang *et al.* Visualizing the Fate of Intra-Articular Injected Mesenchymal Stem Cells In Vivo in the Second Near-Infrared Window for the Effective Treatment of Supraspinatus Tendon Tears. *Adv Sci (Weinh)* **6**, 1901018, doi:10.1002/advs.201901018 (2019).

12 A. Dorodnyy *et al.* Mie Scattering for Photonic Devices. *Laser & Photonics Reviews* **17**, 2300055, doi:10.1002/lpor.202300055 (2023).

13 M. C. Oliveros *et al.* ON THE ENTROPY-FLUCTUATION CONTRIBUTION TO RAYLEIGH-SCATTERING OF LIGHT. *Physics Letters A* **108**, 362-364, doi:10.1016/0375-9601(85)90117-3 (1985).

14 Guosong Hong *et al.* Near-infrared fluorophores for biomedical imaging. *Nature Biomedical Engineering* **1**, 0010, doi:10.1038/s41551-016-0010 (2017).

15 S. Stolik *et al.* Measurement of the penetration depths of red and near infrared light in human "ex vivo" tissues. *J Photochem Photobiol B* **57**, 90-93, doi:10.1016/s1011-1344(00)00082-8 (2000).

16 K. E. Luker *et al.* Comparative study reveals better far-red fluorescent protein for whole body imaging. *Sci Rep* **5**, 10332, doi:10.1038/srep10332 (2015).

17 Y. L. Yang *et al.* Molecular fluorophores for in vivo bioimaging in the second near-infrared window. *European Journal of Nuclear Medicine and Molecular Imaging* **49**, 3226-3246, doi:10.1007/s00259-022-05688-x (2022).

18 S. Olsztyńska-Janus *et al.* ATR-IR study of skin components: Lipids, proteins and water. Part II: Near infrared radiation effect. *Spectrochimica Acta Part A: Molecular and Biomolecular Spectroscopy* **202**, 93-101, doi:https://doi.org/10.1016/j.saa.2018.05.036 (2018).

19 Jun Qian *et al.* Aggregation-induced emission fluorophores towards the second near-infrared optical windows with suppressed imaging background. *Coordination Chemistry Reviews* **472**, doi:10.1016/j.ccr.2022.214792 (2022).

20 Shuo Diao *et al.* Fluorescence Imaging In Vivo at Wavelengths beyond 1500 nm. *Angewandte Chemie-International Edition* **54**, 14758-14762, doi:10.1002/anie.201507473 (2015).

21 Feifei Wang *et al.* In vivo non-invasive confocal fluorescence imaging beyond 1,700 nm using superconducting nanowire single-photon detectors. *Nature Nanotechnology* **17**, 653-+, doi:10.1038/s41565-022-01130-3 (2022).

22 Shiyi Peng *et al.* Deep-Tissue, Large-FOV 3D NIR-II Fluorescence Confocal Microscopy With Hundred-Nanosecond Equivalent Pixel Dwell Time. *Laser & Photonics Reviews* **19**, doi:10.1002/lpor.202402307 (2025).

23 Shiyi Peng *et al.* Deep learning-enhanced NIR-II fluorescence volumetric microscopy for dynamic 3D vascular imaging. *Journal of Innovative Optical Health Sciences* **18**, doi:10.1142/s1793545825500130 (2025).

24 T. G. Baboolal *et al.* Synovial fluid hyaluronan mediates MSC attachment to cartilage, a potential novel mechanism contributing to cartilage repair in osteoarthritis using knee joint distraction. *Ann Rheum Dis* **75**, 908-915, doi:10.1136/annrheumdis-2014-206847 (2016).

25 X. Xia *et al.* Single cell immunoprofile of synovial fluid in rheumatoid arthritis with TNF/JAK inhibitor treatment. *Nat Commun* **16**, 2152, doi:10.1038/s41467-025-57361-0 (2025).

26 Donald A. Neumann. in *Kinesiology of the Musculoskeletal System: Foundations for Rehabilitation* (ed Donald A. Neumann) Ch. 13, 541-557 (Elsevier, 2017).

27 N. Lopomo *et al.* Analysis of the influence of anaesthesia on the clinical and quantitative assessment of the pivot shift: a multicenter international study. *Knee Surg Sports*

Traumatol Arthrosc **25**, 3004-3011, doi:10.1007/s00167-016-4130-1 (2017).

28 C. S. Ahmad *et al.* The moving patellar apprehension test for lateral patellar instability. *Am J Sports Med* **37**, 791-796, doi:10.1177/0363546508328113 (2009).

29 Emine Zeynep Ulutas *et al.* High-resolution in vivo kinematic tracking with customized injectable fluorescent nanoparticles. *Science Advances* **11**, eadu9136, doi:doi:10.1126/sciadv.adu9136 (2025).

30 A. Monsees *et al.* Estimation of skeletal kinematics in freely moving rodents. *Nat Methods* **19**, 1500-1509, doi:10.1038/s41592-022-01634-9 (2022).

31 D. Zou *et al.* Medial stability and lateral flexibility of the collateral ligaments during mid-range flexion in medial-pivot total knee arthroplasty patients demonstrates favorable postoperative outcomes. *Knee Surg Sports Traumatol Arthrosc* **31**, 3734-3744, doi:10.1007/s00167-023-07307-0 (2023).

32 J. Frings *et al.* Objective assessment of patellar maltracking with 3 T dynamic magnetic resonance imaging: feasibility of a robust and reliable measuring technique. *Sci Rep* **10**, 16770, doi:10.1038/s41598-020-72332-9 (2020).

33 J. Frings *et al.* Dynamic Mediolateral Patellar Translation Is a Sex- and Size-Independent Parameter of Adult Proximal Patellar Tracking Using Dynamic 3 Tesla Magnetic Resonance Imaging. *Arthroscopy* **38**, 1571-1580, doi:10.1016/j.arthro.2021.10.014 (2022).

34 W. Yi *et al.* A NIR-II fluorescent probe for articular cartilage degeneration imaging and osteoarthritis detection. *Biomater Sci* **7**, 1043-1051, doi:10.1039/c8bm01440j (2019).

35 Robert H. Miller. in *Campbell's Operatvie Orthopaedics Thirteen Edition* (ed Frederick M. Azar) Ch. 45, 2123-2283 (Elsevier, 2017).

36 S. Zhou *et al.* Lymphatic vessels: roles and potential therapeutic intervention in rheumatoid arthritis and osteoarthritis. *Theranostics* **14**, 265-282, doi:10.7150/thno.90940 (2024).

37 J. Pradal *et al.* Effect of particle size on the biodistribution of nano- and microparticles following intra-articular injection in mice. *Int J Pharm* **498**, 119-129, doi:10.1016/j.ijpharm.2015.12.015 (2016).

38 Y. Kong *et al.* Highly Fluorescent Ribonuclease-A-Encapsulated Lead Sulfide Quantum Dots for Ultrasensitive Fluorescence in Vivo Imaging in the Second Near-Infrared Window. *Chem Mater* **28**, 3041-3050, doi:10.1021/acs.chemmater.6b00208 (2016).

39 J. Chen *et al.* In Vivo Dynamic Monitoring of Bacterial Infection by NIR-II Fluorescence Imaging. *Small* **16**, e2002054, doi:10.1002/smll.202002054 (2020).

40 S. Waldt *et al.* Diagnostic performance of MR arthrography in the assessment of superior labral anteroposterior lesions of the shoulder. *AJR Am J Roentgenol* **182**, 1271-1278, doi:10.2214/ajr.182.5.1821271 (2004).

41 T. Brindle *et al.* The meniscus: review of basic principles with application to surgery and rehabilitation. *J Athl Train* **36**, 160-169 (2001).

42 T. Higuchi *et al.* An analysis of the medial patellofemoral ligament length change pattern using open-MRI. *Knee Surg Sports Traumatol Arthrosc* **18**, 1470-1475, doi:10.1007/s00167-010-1043-2 (2010).

43 Z. Hu *et al.* First-in-human liver-tumour surgery guided by multispectral fluorescence imaging in the visible and near-infrared-I/II windows. *Nat Biomed Eng* **4**, 259-271, doi:10.1038/s41551-019-0494-0 (2020).

44 Mo Chen *et al.* Tracking the in vivo spatio-temporal patterns of neovascularization via NIR-II fluorescence imaging. *Nano Research* **13**, 3123-3129, doi:10.1007/s12274-020-2982-7 (2020).

45 Y. Chen *et al.* Photoacoustic Tomography with Temporal Encoding Reconstruction (PATTERN) for cross-modal individual analysis of the whole brain. *Nat Commun* **15**, 4228, doi:10.1038/s41467-024-48393-z (2024).

46 X. Meng *et al.* In Vivo Precision Evaluation of Lymphatic Function by SWIR Luminescence Imaging with PbS Quantum Dots. *Adv Sci (Weinh)*, e2206579, doi:10.1002/advs.202206579 (2023).

47 Yuzhou Chen *et al.* In vivo real-time monitoring delayed administration of M2 macrophages to enhance healing of tendon by NIR-II fluorescence imaging. *Nano Research* **17**, 4379-4390, doi:10.1007/s12274-023-6363-x (2024).

48 Orhan Beger *et al.* Effects of different cadaver preservation methods on muscles and tendons: a morphometric, biomechanical and histological study. *Anatomical Science International* **95**, 174-189, doi:10.1007/s12565-019-00508-z (2020).

49 Yuting Ling *et al.* Effects of fixation and preservation on tissue elastic properties measured by quantitative optical coherence elastography (OCE). *Journal of Biomechanics* **49**, 1009-1015, doi:https://doi.org/10.1016/j.jbiomech.2016.02.013 (2016).

50 Philip. H. Cohen. in *BMJ Best Practice* https://bestpractice.bmj.com/topics/en-gb/589 (2025).

51 M. J. Tanaka *et al.* In Vivo Length Changes Between the Attachments of the Medial Patellofemoral Complex Fibers in Knees With Anatomic Risk Factors for Patellar Instability. *Am J Sports Med* **51**, 1765-1776, doi:10.1177/03635465231165296 (2023).

52 Yingying Jiang *et al.* In vivo imaging of the immune system. *Nature Reviews Bioengineering*, doi:10.1038/s44222-026-00407-9 (2026).

53 G. P. Bhat *et al.* Structured wound angiogenesis instructs mesenchymal barrier compartments in the regenerating nerve. *Neuron* **112**, 209-229., doi:10.1016/j.neuron.2023.10.025 (2024).

54 R. Hussain *et al.* Potentiating glymphatic drainage minimizes post-traumatic cerebral oedema. *Nature* **623**, 992-1000, doi:10.1038/s41586-023-06737-7 (2023).

55 A. P. Ligocki *et al.* Cerebrospinal fluid flow extends to peripheral nerves further unifying the nervous system. *Sci Adv* **10**, eadn3259, doi:10.1126/sciadv.adn3259 (2024).

56 A. Grüneboom *et al.* Next-generation imaging of the skeletal system and its blood supply. *Nat Rev Rheumatol* **15**, 533-549, doi:10.1038/s41584-019-0274-y (2019).

57 U.S. Food & Drug Administration. in *New Drug Application (NDA)* https://www.fda.gov/drugs/types-applications/new-drug-application-nda (2022).

58 B. W. Pogue *et al.* Review of successful pathways for regulatory approvals in open-field fluorescence-guided surgery. *J Biomed Opt* **26**, doi:10.1117/1.Jbo.26.3.030901 (2021).

Dear reviewers,

Thank you for your valuable assessments and constructive feedback on our manuscript entitled "Next-generation functional diagnostic paradigm for musculoskeletal diseases based on live SWIR imaging." (NCOMMS-25-72766A). We have carefully considered all the comments and suggestions, and have incorporated the corresponding revisions into the manuscript, with the changes highlighted in yellow. We hope that our responses and revisions adequately address the reviewers' concerns.

Thank you again for your time and consideration.

Point-to-point responses:
Reviewer #1 (Remarks to the Author):

I would like to thank the authors for addressing most of my concerns. While the manuscript has improved following revision, I still believe it is important to clearly outline the limitations of the approach within the manuscript itself, rather than only in the response to my previous comment (Major Comment 3 in the earlier review).

Explicit discussion of these limitations would help readers appropriately interpret the findings and place the results in their proper context. I therefore strongly encourage the authors to include a dedicated paragraph in the Discussion that explicitly addresses the potential limitations of the study.

Response: Thank you for your valuable comments regarding the necessity of limitation parts. The limitations were now explicitly included in the discussion part of the manuscript.
Revision (Line 518): The clinical translation of this imaging platform requires consideration of several limitations. First, no FDA-approved SWIR probes are currently available for use in the knee joint, an environment characterized by low serum protein content. Although prior animal studies have demonstrated the safety of the probes used in this work[1], its toxicity profile in humans remains unclear. Thus, future studies should prioritize FDA-approved probes for clinical application. Second, penetration depth remains a fundamental constraint in optical imaging although the SWIR window extends depth to approximately 10 mm[2, 3]. Notably, both prior human studies[4] and our cadaver experiments required skin removal, and deeper structures including ligaments and meniscus in the human knee remained unclear. Developing methods with greater penetration would significantly broaden applicability, enabling visualization of deeper anatomy.

Reviewer #2 (Remarks to the Author):

In the revised manuscript, the authors have addressed all previous suggestions, and the overall quality has significantly improved. I recommend publication after the following minor concerns are addressed:

1. The methodology repeatedly refers to the use of "all available quantitative data," yet the number of biological replicates varies considerably across different time points. The authors should clarify

the reasons for the changes in sample size (n-value) over time and specify whether any sample exclusion criteria were applied. (Lines 713–724)

Response: Thank you for raising the point regarding the sample size. To ensure the reliability, the sample size was in accordance with the realistic experimental conditions. Specifically, in this study, the animals underwent longitudinal periods of experiment (28 days), which could inevitably lead to unexpected circumstances including death. In addition, a portion of animals were removed for other experiments including further mechanistic investigations. Therefore, the sample size varies across different time points. Notably, the sample size variation could also be found in other publicated works[5, 6]. And importantly, despite this variation, the sample size at each time point was ensured to comply with the statistical standards. To clarify, we have revised the Methods section according to reviewer's suggestions.

2. While the study demonstrates intra-articular clearance and includes HE analysis of major organs in the methodology, the use of quantum dot PbS raises additional safety considerations. More comprehensive evidence regarding the biosafety of the materials used is recommended.

Response: Thank you for your valuable comments. The biosafety of this SWIR probes have been thoroughly investigated in prior studies[1] (Figure R1-3, Table R1).

1) Long-term in vitro cell biosafety

Since the cell cycle influences cell fate transitions beyond proliferation, a cell cycle assay was conducted to evaluate the influence of fluorophores on MSCs (Mesenchymal Stem Cells)[1]. No significant changes in the cell cycle were observed in MSCs with different concentrations of PbS QDs (Figure R1A). Additionally, a colony-forming assay, regarded as a reliable indicator of pluripotent stem cell activity, demonstrated homogeneous colony size and morphology with no significant differences in colony numbers (Figure R1B), suggesting that the fluorophores were safe for cell self-renewal potential. To further assess the influence on differentiation capacity, adipogenic and osteogenic differentiation assays were performed, revealing successful differentiation into osteoblast and adipocyte lineages at PbS QDs concentrations up to 30 μg/mL. Similar intracellular lipid-rich vacuoles, alkaline phosphatase (ALP) activity, and calcium deposits were observed across different concentrations (Figure R1C). These results indicate that PbS QDs did not impair MSC multipotentiality and demonstrated high long-term biosafety.

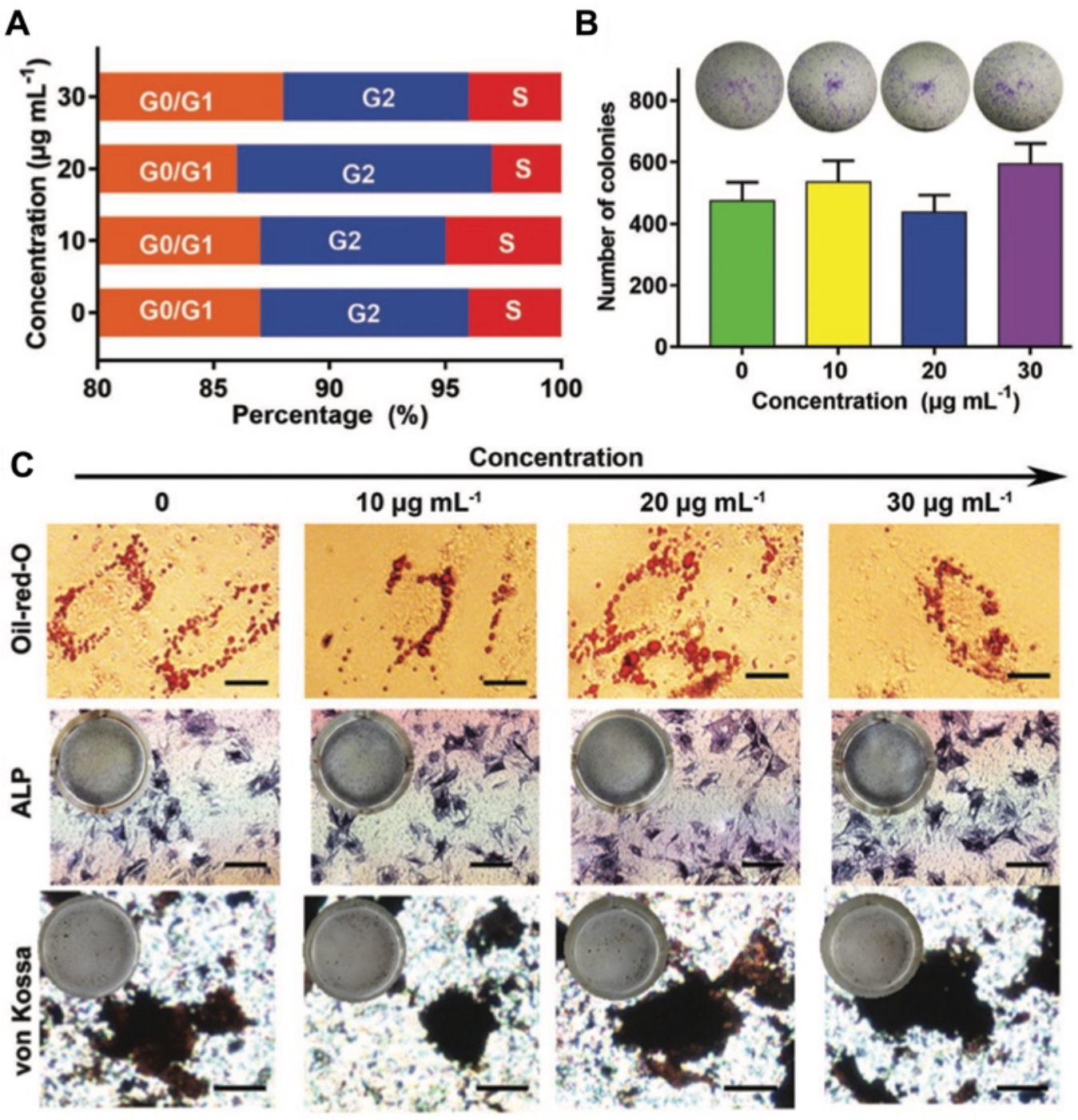

Figure R1. Interactions of different concentrations of Ribonuclease-A-encapsulated PbS QDs with MSCs. (A) Cell cycles detected by flow cytometry. (B) Comparisons of colony-formation efficiency in each group. (C) Adipogenic and osteogenic differentiation assays for labeled MSCs, scale bars represent 20 μm (oil-red O staining), 200 μm (ALP staining) and 50 μm (von Kossa staining).(Figure from [1])

Moreover, to assess MSC proliferation following incubation with PbS QDs, both live/dead staining and a CCK-8 assay were performed. Representative images confirmed that nearly all cells remained viable in each group (Figure R2A). No significant differences were observed in MSC morphology or viability (Figure R2B). Similarly, no statistical differences were detected between the 10 μg mL^{-1} group and other groups at the same time point ($p > 0.05$) (Figure R2C). These findings suggested that PbS QDs exhibit negligible cytotoxicity toward MSCs at concentrations ranging from 10 to 30 μg mL^{-1}.

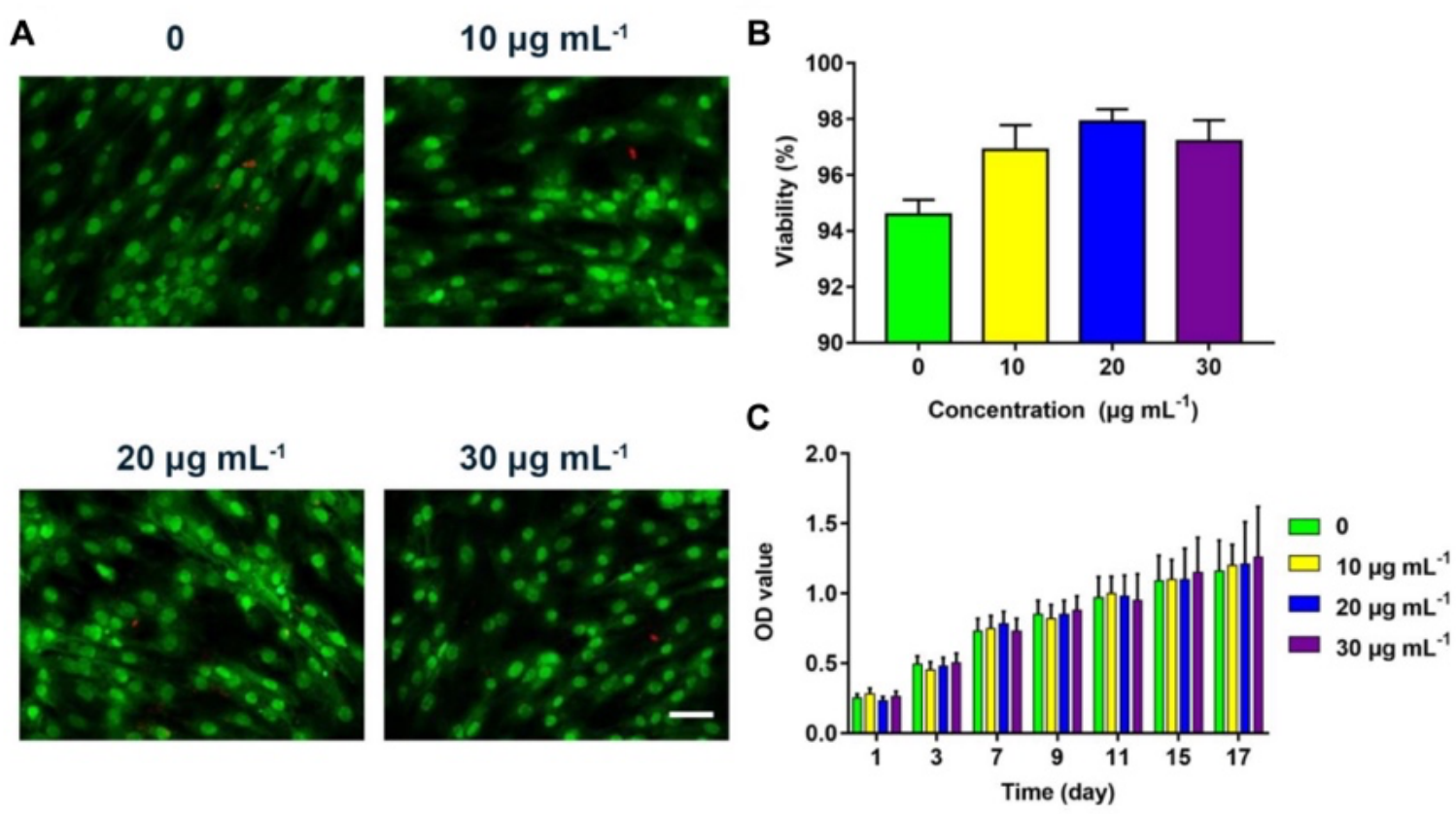

Figure R2. The effects of different concentrations of PbS QDs on cell proliferation and cell viability. (A). Live/dead cell staining for labeled MSCs (green fluorescence-live cells, red fluorescence-dead

cells). Scale bars represent 50 μm. (B). Quantification analysis of cell viability of labeled MSCs. (C) Cell proliferation of labeled MSCs determined by CCK-8.(Figure from [1])

2) Long-term In vivo biosafety

In addition, long-term Pb ion level analysis of blood, feces, urine and major organs have been conducted in previous study[1] to evaluate the biosafety and clearance of PbS QDs fluorophores. Low concentration of Pb ion level was observed in blood, feces and urine in long-term analysis and the fluorophores did not deposit in major organs, suggesting high biosafety of our PbS QDs (Figure R3).

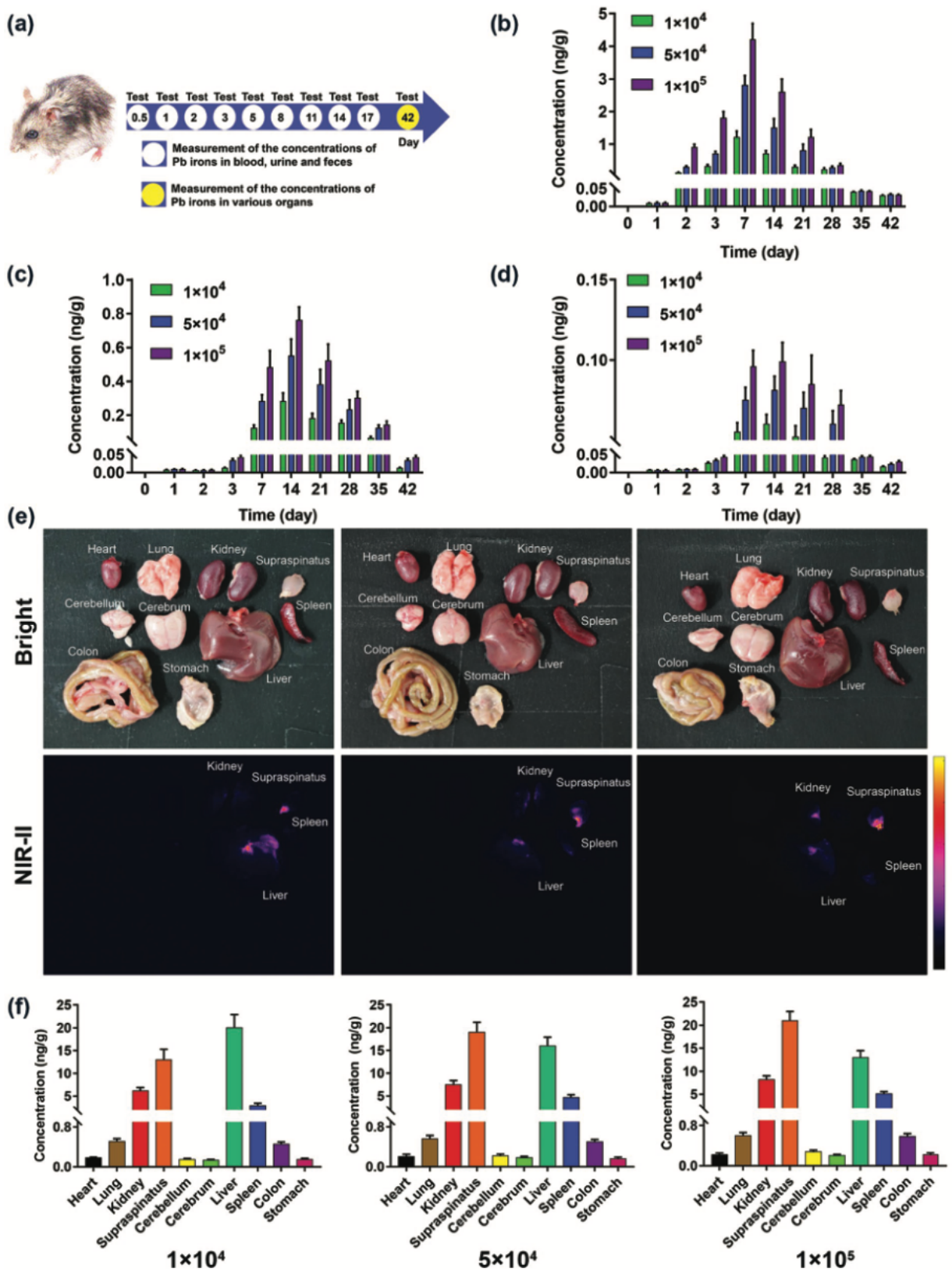

Figure R3. *In vivo* quantitative analysis of the distribution and clearance processes. a) Schematic illustration of the study protocol of investigating the clearance of Pb ions. b) Determination of the concentration of Pb ions in whole blood. c) Determination of the concentration of Pb ions s in feces. d) Determination of the concentration of Pb ions in urine. e) Bright field and NIR-II fluorescence images of various organs collected from the mice at 42 days after post-injection. f) Determination of the concentration of Pb ions in major organs. (Figure from [1])

3) Organ function biosafety

Moreover, the indicators of organ function were also measured and demonstrated in the Table R1, which showed all parameters were in range of normal value, indicating the low biotoxicity of QDs in current study.

Table R1. Indicators for liver and kidney function

Indicators	Mean ± SD	Normal value	In normal region

Liver Function			
ALT	60.42 ± 22.97 U/L	10.06-96.47 U/L	Yes
AST	189.17 ± 50.22 U/L	36.31-235.48 U/L	Yes
GGT	2.41 ± 0.98 U/L	0-7.78 U/L	Yes
ALP	197.56 ± 46.28 U/L	22.52-474.35 U/L	Yes
D-BIL	5.10 ± 0.49 μmol/L	0.45-33.89 μmol/L	Yes
T-BIL	12.73 ± 1.71 μmol/L	6.09-53.06 μmol/L	Yes
TBA	1.60 ± 0.45 μmol/L	0-8.51 μmol/L	Yes
ALB	31.09 ± 0.53 g/L	21.22-39.15 g/L	Yes
Kidney Function			
BUN	11.92 ± 1.40 mg/dl	10.81-34.74 mg/dl	Yes
CR	22.32 ± 4.77 μmol/L	10.91-85.09 μmol/L	Yes

3. The relevance of the disease models should be strengthened. Figure 2 employs an acute injury model (tendon transection and fracture), which does not accurately reflect chronic degenerative diseases such as osteoarthritis or rheumatoid arthritis. Nevertheless, the manuscript repeatedly refers to the diagnostic relevance of such conditions, indicating a weak alignment between the model and the intended clinical context. Similarly, the tibial defect model in Figures 4–5 represents a bone regeneration paradigm rather than a bone disease model. Its suitability for demonstrating functional diagnosis of bone diseases should be justified more clearly.

Response: Thank you for raising these important points. Musculoskeletal diseases refer to a large variety of health conditions that affect muscles, joints, ligaments, tendons, bones and cartilage[7, 8]. Among them, osteoarthritis is a chronic arthropathy characterized by disruption of joint cartilage[9] and rheumatoid arthritis is a chronic systemic autoimmune disease that primarily involves the joints[10]. Therefore, osteoarthritis and rheumatoid arthritis are kind of musculoskeletal diseases, which was also demonstrated in Medical Subject Headings (MeSH) terminology in the PubMed database[8].

Notably, as stated in title and abstract, the primary focus of the study is to visualize the kinematics during locomotion and enabling the general functional diagnosis of the musculoskeletal diseases based on the typical animal model. Accordingly, the animal model in Figure 2, which is an acute muscle and tendon injury model, is classified as a type of "musculoskeletal disease" according to the MeSH termminology[8]. In addition, the model shown in Figures 4 and 5 is a bone defect, which is primarily the result of trauma, bone tumors, and bone infections[11]. These conditions were also categorized under "musculoskeletal diseases" according to MeSH terminology[8]. Therefore, both models fall within the overall scope of the manuscript.

Furthermore, it was reported that these acute injuries could lead to the development of chronic degenerative diseases, including osteoarthritis[12]. The proposed imaging approach could lay the foundation for assessing the kinematics of such chronic degenerative diseases. We are planning to extend its application to these chronic degenerative diseases in future investigations.

Finally, to enhance the clarity of the theme, the manuscript has been revised according to the

reviewer's suggestion.

[1] YANG Y, CHEN J, SHANG X, et al. Visualizing the Fate of Intra-Articular Injected Mesenchymal Stem Cells In Vivo in the Second Near-Infrared Window for the Effective Treatment of Supraspinatus Tendon Tears [J]. Adv Sci (Weinh), 2019, 6(19): 1901018.

[2] YANG Y L, ZHANG F. Molecular fluorophores for in vivo bioimaging in the second near-infrared window [J]. European Journal of Nuclear Medicine and Molecular Imaging, 2022, 49(9): 3226-3246.

[3] OLSZTYŃSKA-JANUS S, KIEŁBOWICZ Z, CZARNECKI M A. ATR-IR study of skin components: Lipids, proteins and water. Part II: Near infrared radiation effect [J]. Spectrochimica Acta Part A: Molecular and Biomolecular Spectroscopy, 2018, 202: 93-101.

[4] HU Z, FANG C, LI B, et al. First-in-human liver-tumour surgery guided by multispectral fluorescence imaging in the visible and near-infrared-I/II windows [J]. Nat Biomed Eng, 2020, 4(3): 259-271.

[5] SHELLARD A, MAYOR R. Collective durotaxis along a self-generated stiffness gradient in vivo [J]. Nature, 2021, 600(7890): 690-694.

[6] ULUTAS E Z, PRADHAN A, KOVEAL D, et al. High-resolution in vivo kinematic tracking with customized injectable fluorescent nanoparticles [J]. Science Advances, 2025, 11(40): eadu9136.

[7] MARTIN C. Musculoskeletal Diseases [M]//MICHALOS A C. Encyclopedia of Quality of Life and Well-Being Research. Dordrecht; Springer Netherlands. 2014: 4228-4231.

[8] NATIONALLIBRARYOFMEDICINE. Musculoskeletal Diseases [J/OL] 1992. https://www.ncbi.nlm.nih.gov/mesh/?term=musculoskeletal+disease.

[9] YASEEN K. Osteoarthritis (OA) [J/OL] 2024. https://www.merckmanuals.com/professional/musculoskeletal-and-connective-tissue-disorders/joint-disorders/osteoarthritis-oa.

[10] YASEEN K. Rheumatoid Arthritis (RA) [J/OL] 2024. https://www.merckmanuals.com/professional/musculoskeletal-and-connective-tissue-disorders/joint-disorders/rheumatoid-arthritis-ra.

[11] MIGLIORINI F, LA PADULA G, TORSIELLO E, et al. Strategies for large bone defect reconstruction after trauma, infections or tumour excision: a comprehensive review of the literature [J]. Eur J Med Res, 2021, 26(1): 118.

[12] WHITTAKER J L, CULVENOR A G, JUHL C B, et al. OPTIKNEE 2022: consensus recommendations to optimise knee health after traumatic knee injury to prevent osteoarthritis [J]. Br J Sports Med, 2022, 56(24): 1393-1405.
