# Supplementary material for: Next-generation functional diagnostic paradigm for musculoskeletal diseases based on live SWIR imaging
Source: Nat Commun. 2026 Sep 14;17:11269. doi: 10.1038/s41467-026-77137-4 (PMC13720147; doi:10.1038/s41467-026-77137-4)
Supplement: Supplementary file 3 [file 41467_2026_77137_MOESM1_ESM.pdf]

Next-generation functional diagnostic paradigm for musculoskeletal diseases based on live SWIR imaging

Huizhu Li[1#], Sijia Feng[1#], Juan Liyau[2], Huaixuan Sheng[1], Shunyao Li[1], Xiao Zhang[1], Mo Chen[3], Liman Sai[4], Lu Fang[5], Chengxuan Yu[1], Kai Xu[1], Yan Wo[6], You Liang[2], Shiyi Chen[1], Yueming Wang[6*], Fuchun Chen[5*], Yunxia Li[1*], Jun Chen[1,7*]

1 Department of Sports Medicine, Huashan Hospital, Fudan University, Shanghai 200040, China.

2 Department of Mathematics, Toronto Metropolitan University, Toronto, ON, Canada.

3 Department of Joint Replacement, Sports Medicine, and Trauma, Department of Orthopedics, Renji Hospital, Shanghai Jiaotong University School of Medicine, Shanghai, 200001, China.

4 Department of Physics, Shanghai Normal University, Shanghai 200234, China.

5 Shanghai Institute of Technical Physics (SITP) of the Chinese Academy of Sciences (CAS) 200083, China.

6 Department of Anatomy and Physiology, Shanghai Jiao Tong University School of Medicine, Shanghai 201318, China.

7 Institutes of Integrative Medicine, Fudan University, Shanghai 200032, China.

#These authors contributed equally to this work.

Corresponding author*

Yueming Wang: fiona.w@sjtu.edu.cn

Fuchun Chen: fuchun.chen@mail.sitp.ac.cn

Yunxia Li: liyunxia912@aliyun.com

Jun Chen: biochenjun@fudan.edu.cn

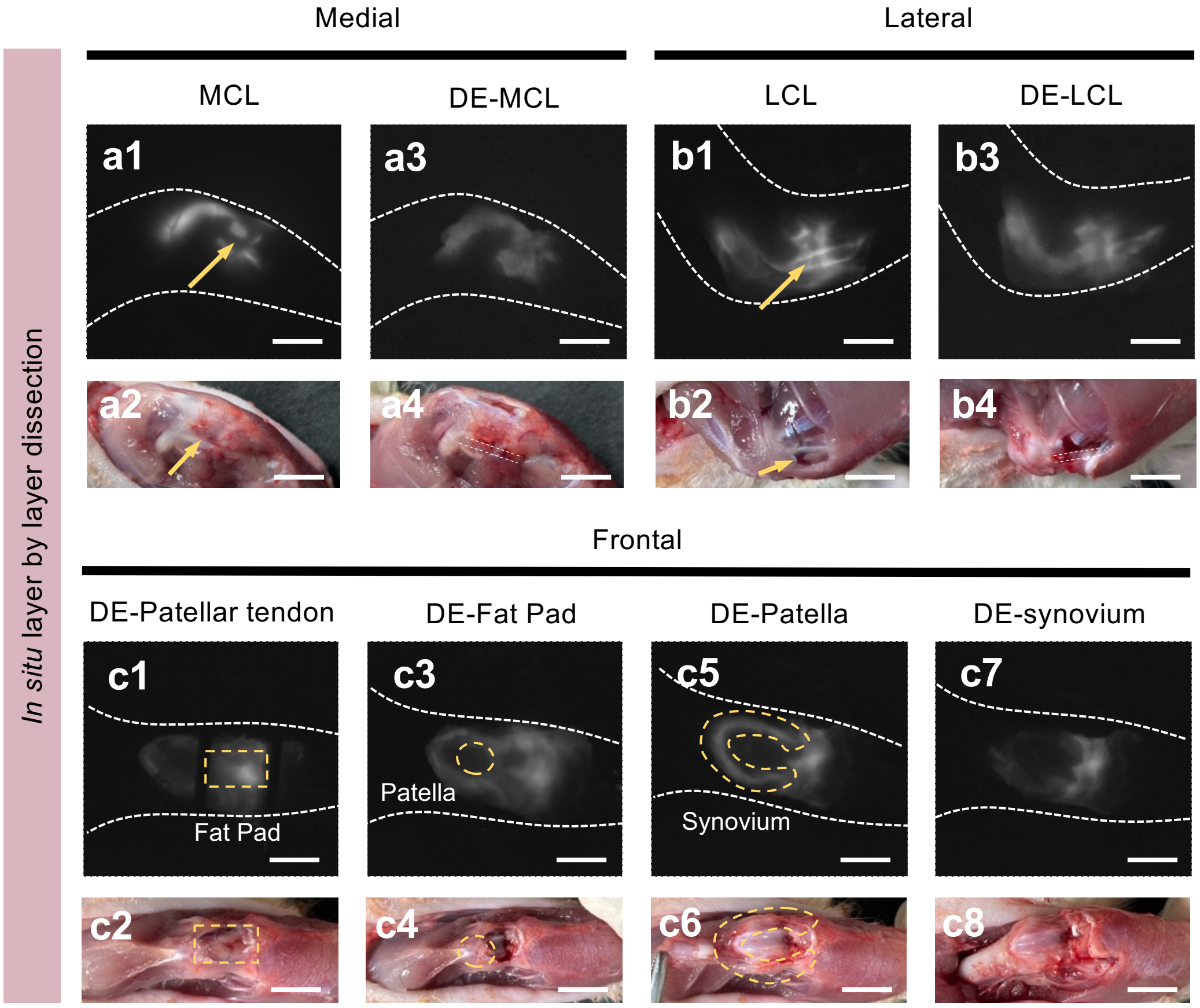

Supplementary Fig. 1. SWIR images of knee anatomic structures with layer-by-layer dissection. (a) SWIR image with intact MCL (a1) and image after removing MCL (a2). **(b)** SWIR image of intact LCL (b1) and image after removing LCL (b2). **(c)** SWIR images of knee structures from frontal view after removing patellar tendon (c1), fat pad (c2), patella (c3) and synovium (c4). PT-patellar tendon. Scale bar:1 cm. MCL, Medial collateral ligament; LCL, Lateral collateral ligament.

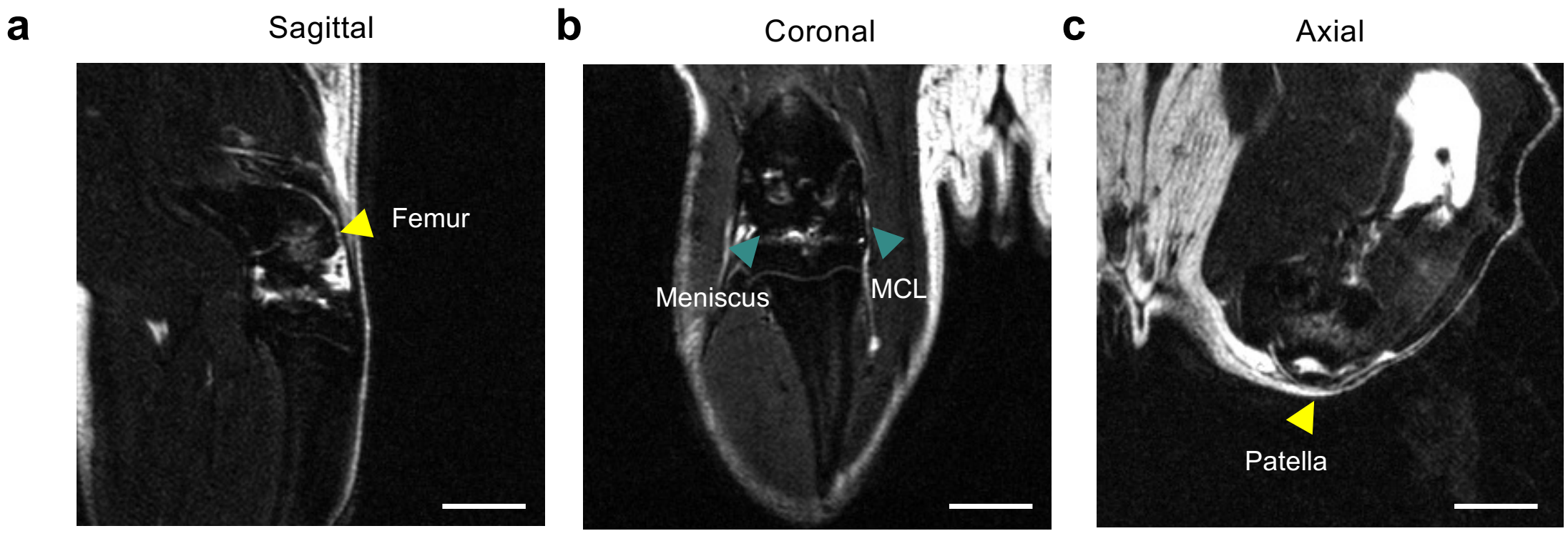

Supplementary Fig. 2. MRI image sections of normal rat knee. (a-c) MRI images of normal rat knee from sagittal (a), coronal (b) and axial (c) view. Scale bar: 0.5 cm.

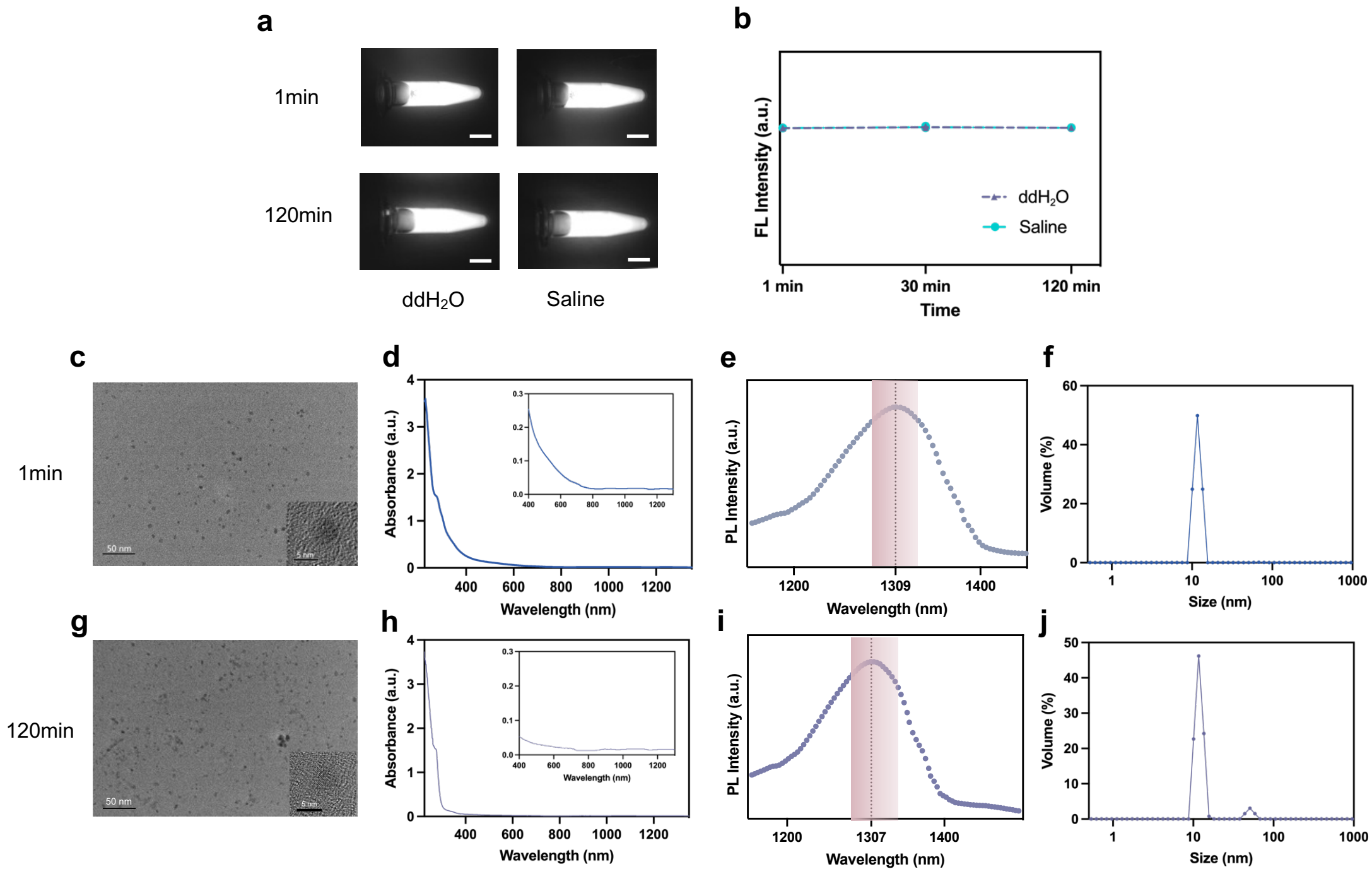

Supplementary Fig. 3. Characterization of SWIR fluorophores. (a) SWIR fluorescence images of SWIR fluorophores under the excitation of 808 nm with ddH_2O and saline buffer after 1 min and 120 min of synthesis. Scale bar: 0.5 cm. (b) FL intensity variation of SWIR fluorophores after 1 min, 30 min, 120 min of synthesis. **(c-f)** TEM images (c), absorbance spectrum (d), photoluminescence spectrum (e) size distribution (f) of SWIR fluorophores 1 min after synthesis. **(g-j)** TEM images (g), absorbance spectrum (h), photoluminescence spectrum (i) size distribution (j) of SWIR fluorophores 120 min after synthesis.

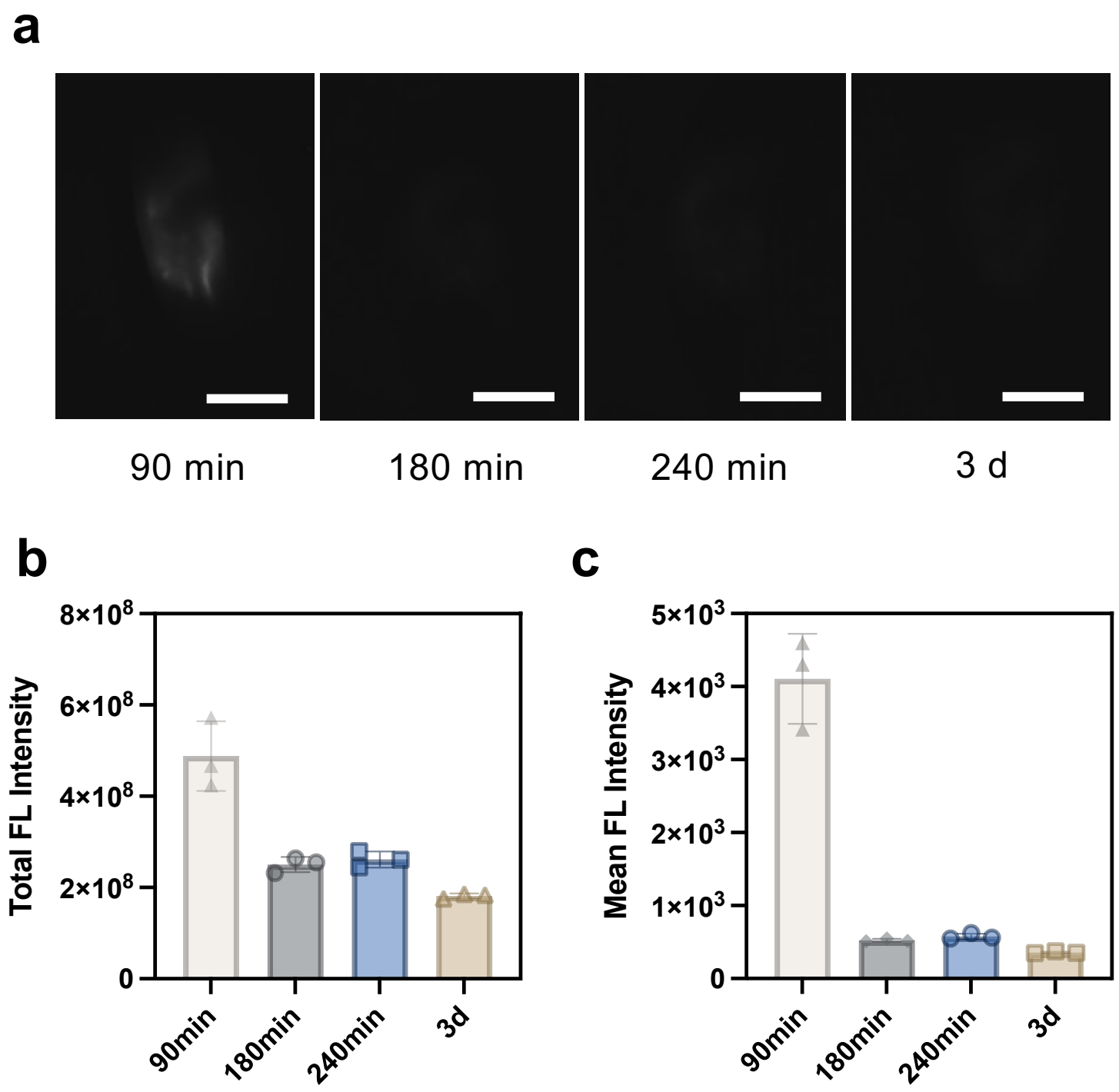

Supplementary Fig. 4. Clearance of SWIR fluorophores. (a) SWIR image of knee structures after 90 min, 180 min, 240 min, 3 d after injection. **(b-c)** Total FL intensity (b) and mean FL intensity (c) measured from (a). Scale bar: 1 cm. Three independent animals were included (n=3).

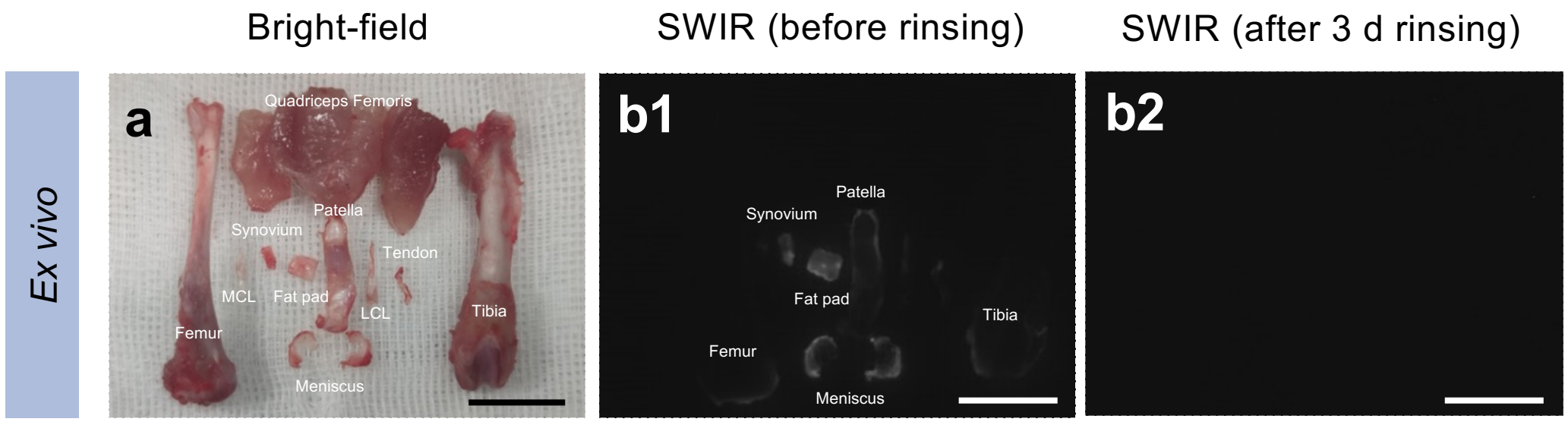

Supplementary Fig. 5. Ex vivo observation of anatomic structures. (a) *Ex vivo* bright field image of knee anatomic structures. **(b)** SWIR image of knee anatomic structures (b1) and after rinsing (b2). Scale bar:1 cm.

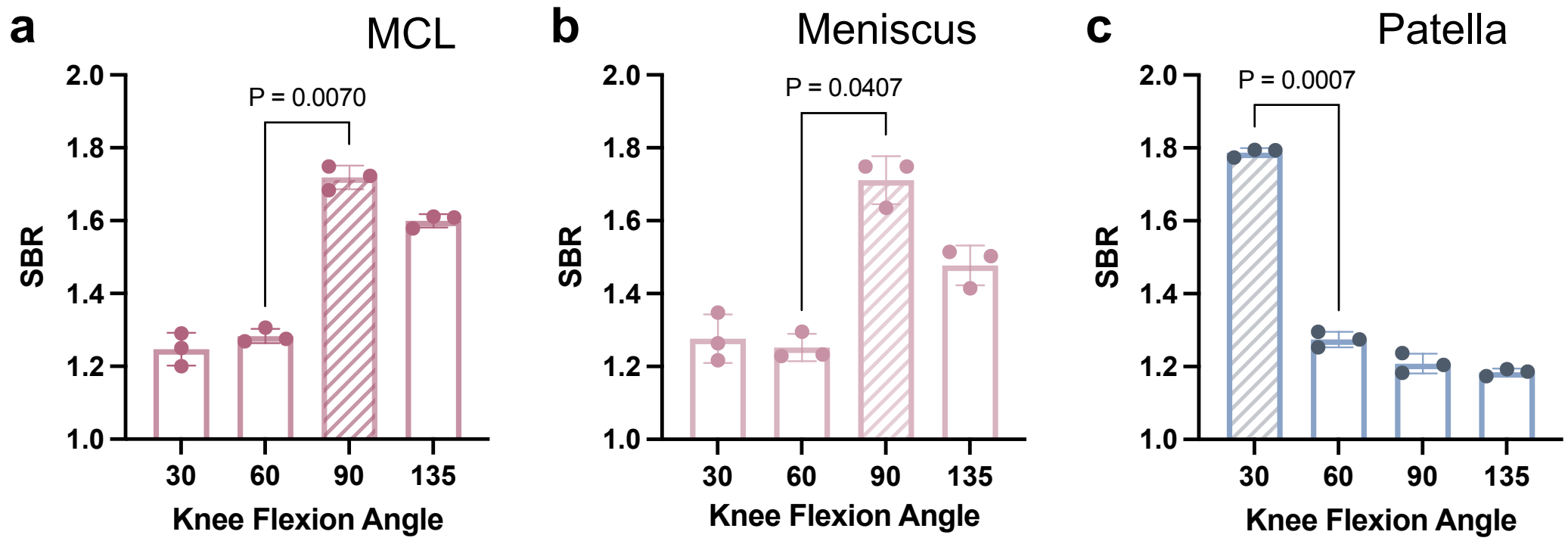

Supplementary Fig. 6. Signal to background ratio (SBR) variation of anatomic structures. (a-c) Signal to background ratios of MCL (a), meniscus (b), and patella (c) at representative knee flexion angles. Three independent animals were included (n=3). P values were calculated by repeated measures ANOVA with Tukey's test for multiple comparisons.

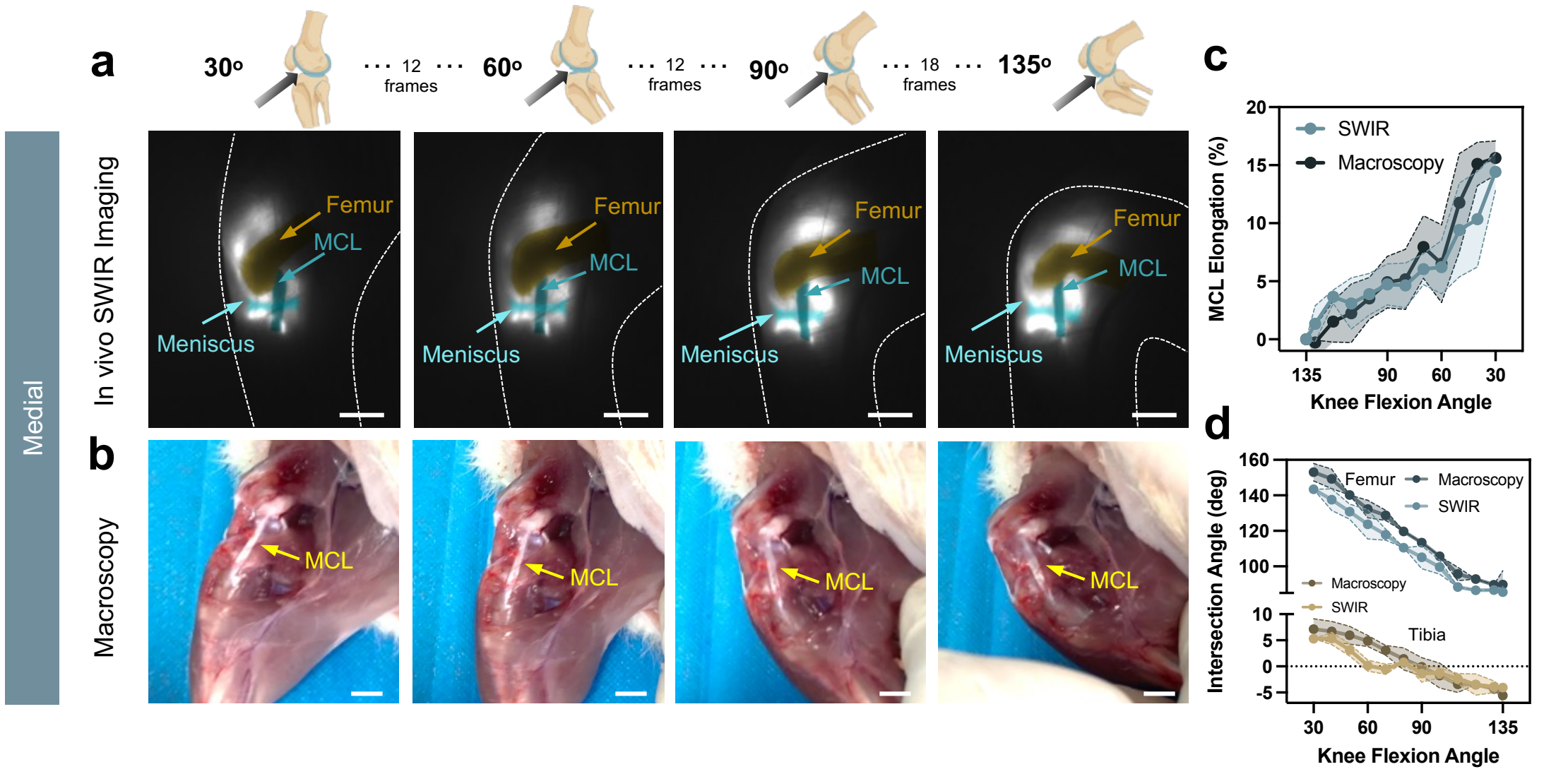

Supplementary Fig. 7. Validation of MCL kinematics by comparing the motion kinematics measured from SWIR imaging and macroscopy. (a-b) SWIR (a) and bright-field macroscopic (b) images of knee anatomic structures with representative flexion angles from medial view. Dark yellow: Bone (Femur), Teal blue: MCL, Mint: Meniscus. **(c)** MCL elongation during knee flexion, which was compared between data measured from SWIR imaging and bright-field macroscopy. **(d)** Intersection angle of MCL-Femur and MCL-Tibia during knee flexion, which was compared between data measured from SWIR imaging and bright-field macroscopy. Scale bar: 0.5 cm. Three independent animals were included (n=3).

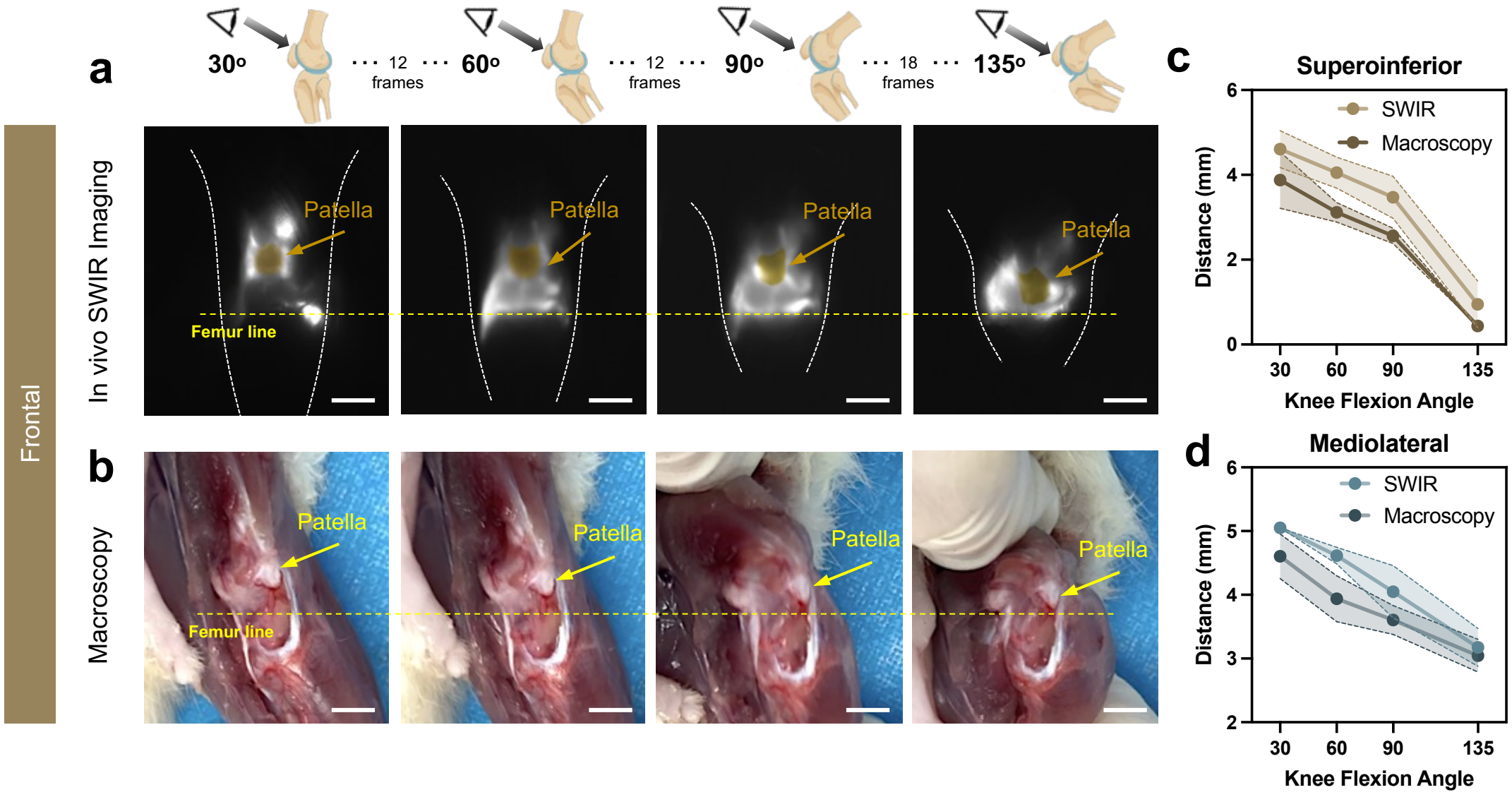

Supplementary Fig. 8. Validation of patella motion kinematics by comparing the patella displacement measured from SWIR imaging and macroscopy. (a-b) SWIR and bright-field macroscopic mages of knee anatomic structures with representative flexion angles from frontal view. Dark yellow: Bone (Patella). **(c-d)** Superoinferior and mediolateral patella displacement with varying knee flexion angles, which was compared between data measured from SWIR imaging and bright-field macroscopy. MCL: Medial collateral ligament. Scale bar: 0.5 cm. Three independent animals were included (n=3).

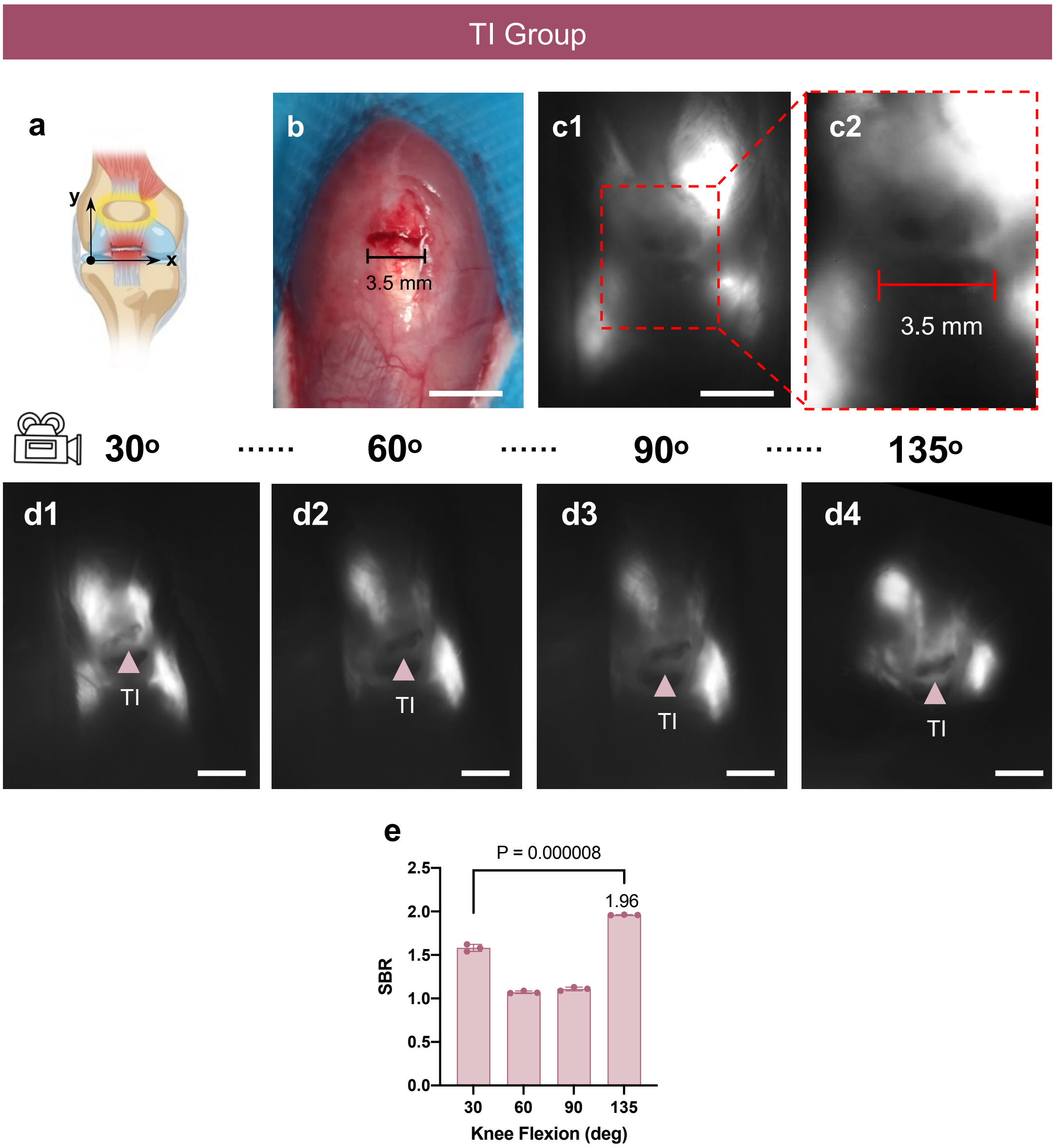

Supplementary Fig. 9. SWIR in vivo dynamic imaging of rat tendon injury (TI) model. (a) Schematic of rat TI model. **(b-c)** Bright field photograph (b), SWIR images (c1), and zoom-in image (c2) of TI. **(d1-d4)** SWIR images of TI from the coronal view with the flexion angle of 30°(d1), 60°(d2), 90° (d3), 135° (d4). **(e)** Signal to background signals of TI. Three independent animals were included (n=3). P values were calculated by repeated measures ANOVA with Tukey's test for multiple comparisons.

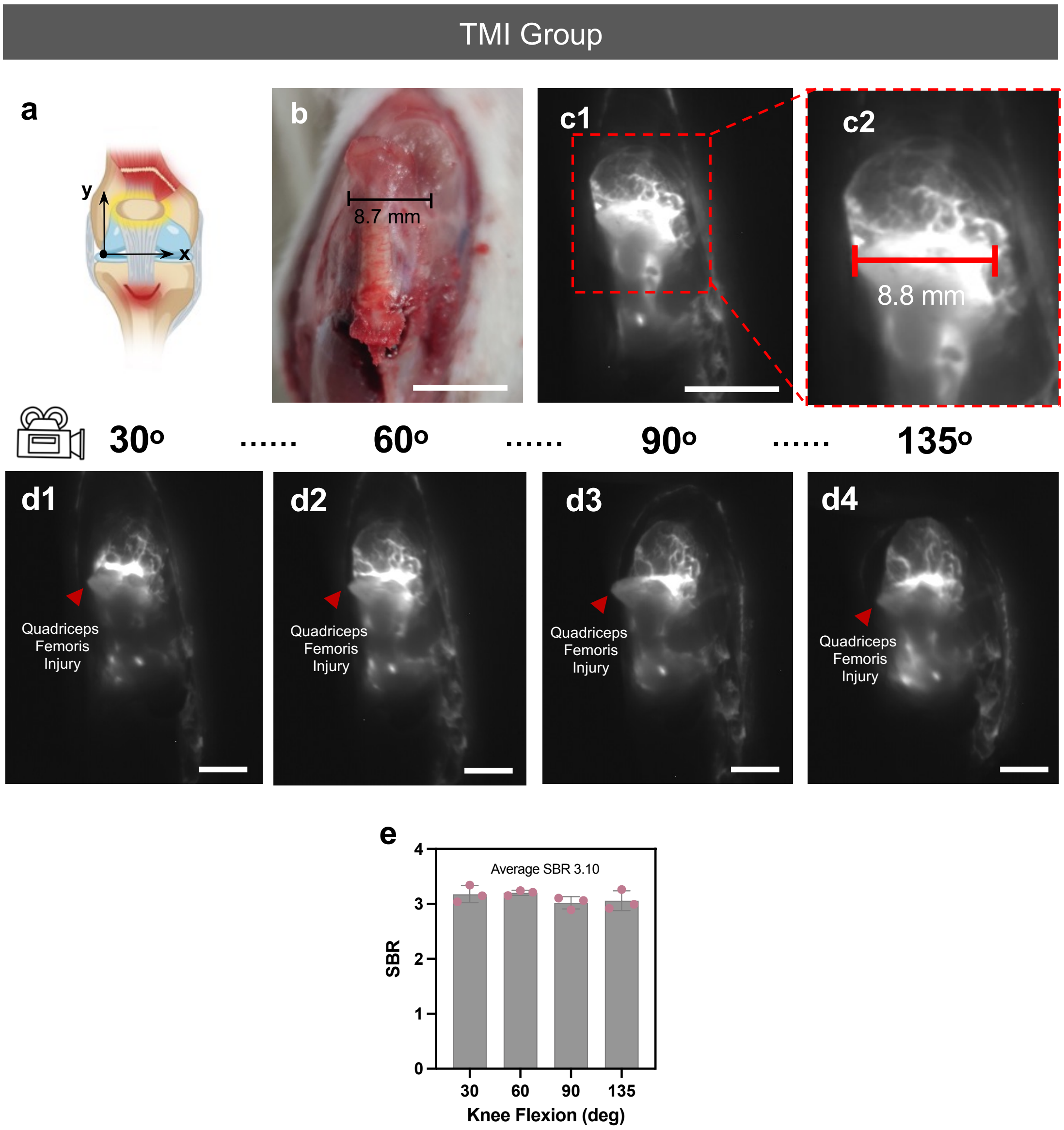

Supplementary Fig. 10. SWIR in vivo dynamic imaging of rat combined tendon and muscle injuries (TMI) model. (a) Scheme of rat TMI model. **(b-c)** Bright field photograph (b), SWIR image (c1), and zoom-in image (c2) of TMI. Scale bar: 1 cm. **(d1-d4)** SWIR images of TMI from the coronal view with the flexion angle of 30°(d1), 60°(d2), 90° (d3), 135° (d4). Scale bar: 0.5 cm. **(e)** Signal to background ratio (SBR) of injured muscle. Three independent animals were included (n=3).

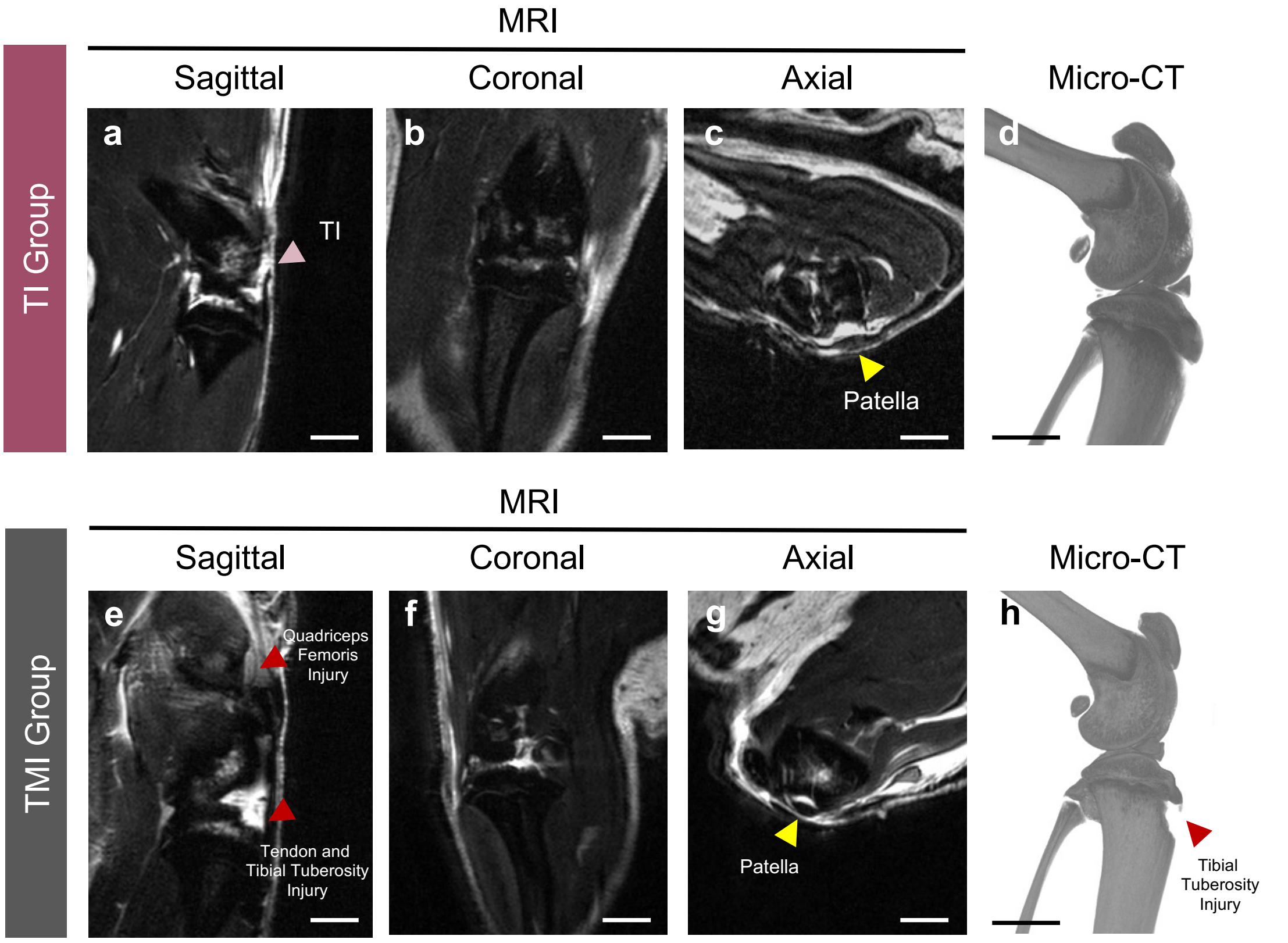

Supplementary Fig. 11. MRI and micro-CT of rat tendon injury (TI) model and muscle injuries (TMI) model. (a-d) MRI images from sagittal (a), coronal (b), axial (c) view, and micro-CT image (d) of TI model. **(e-h)** MRI images from sagittal (e), coronal (f), axial (g) view, and micro-CT image (h) of TMI model. Scale bar: 0.5 cm.

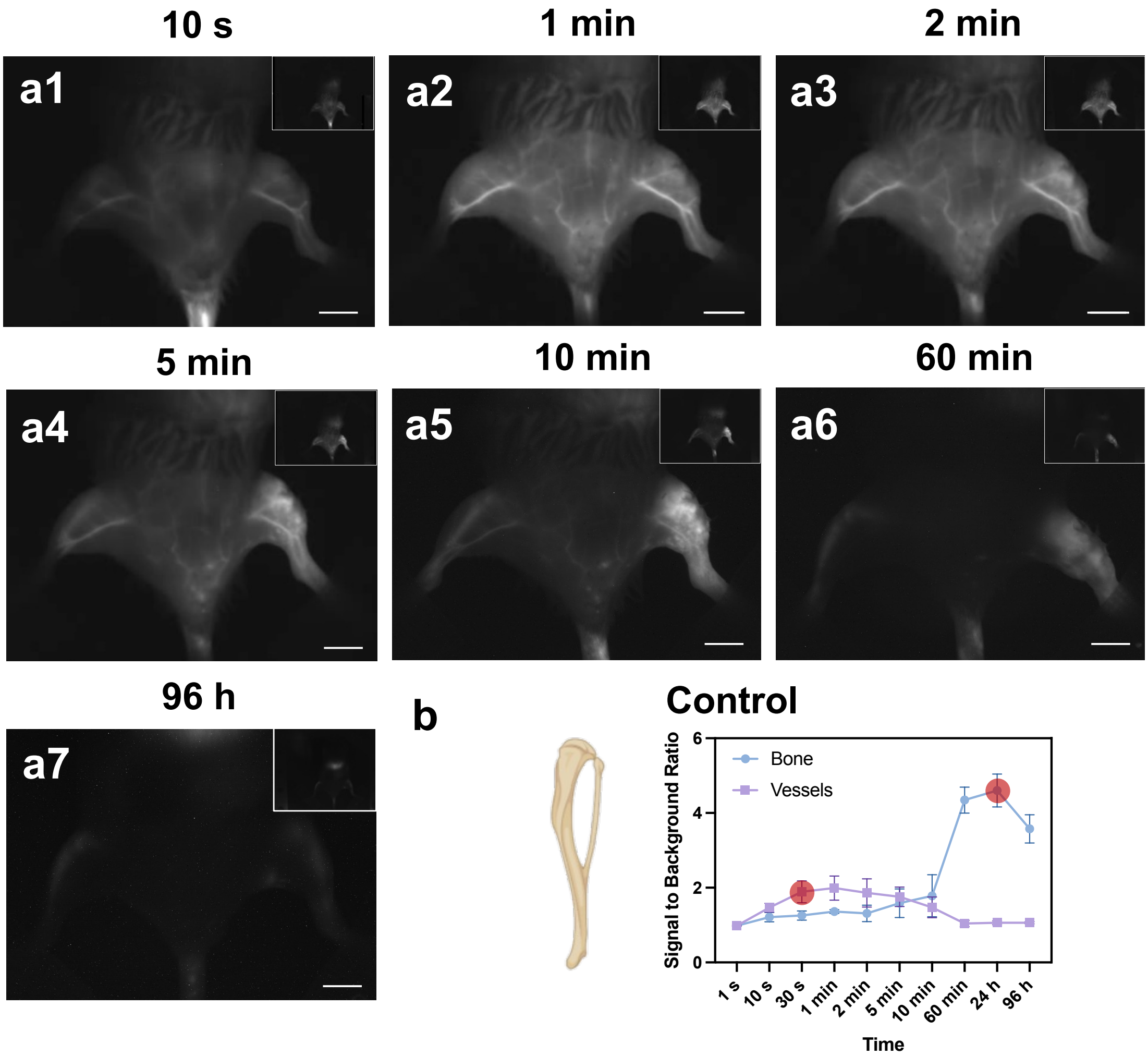

Supplementary Fig. 12. Time window of mouse tibial defect model. (a1-a7) SWIR images of tibial defect mouse model at 10 s, 1 min, 2 min, 5 min, 10 min, 60 min, 96 h after tail vein injection of SWIR fluorophores. SWIR images obtained at 1 s, 30 s, and 24 h after tail vein injection were showed in Figure 4a. (b) Calculated SBR of bone and vessels from control side. Scale bar: 1 cm. Three independent animals were included (n=3).

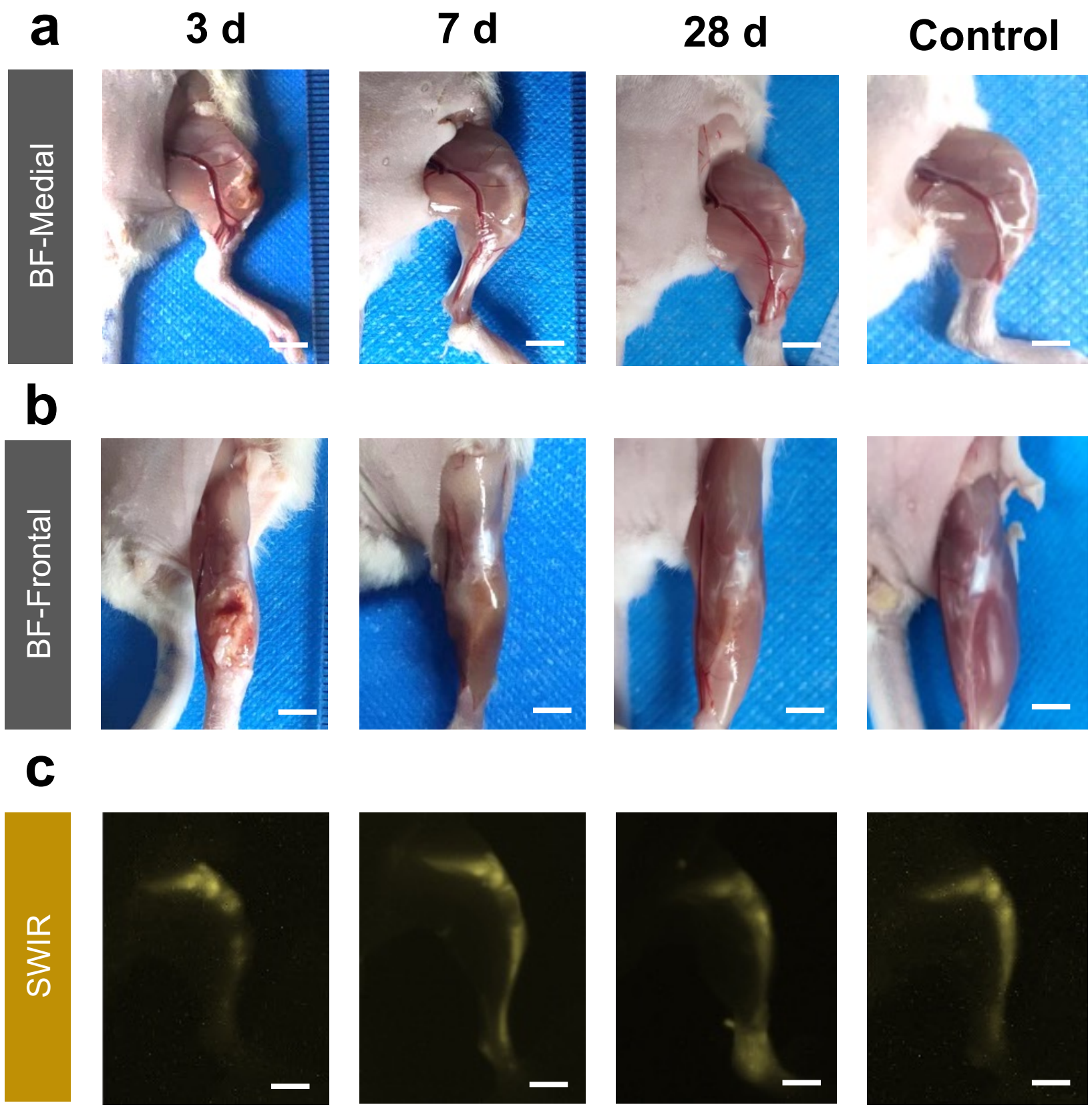

Supplementary Fig. 13. *In situ* analysis of mouse tibial defect model. (a-b) Bright field (BF) macroscopy images from medial (a) and frontal view (b). **(c)** *In situ* SWIR images with skin dissection at 3 d, 7 d, 28 d post-surgery from medial view. Scale bar: 0.5 cm.

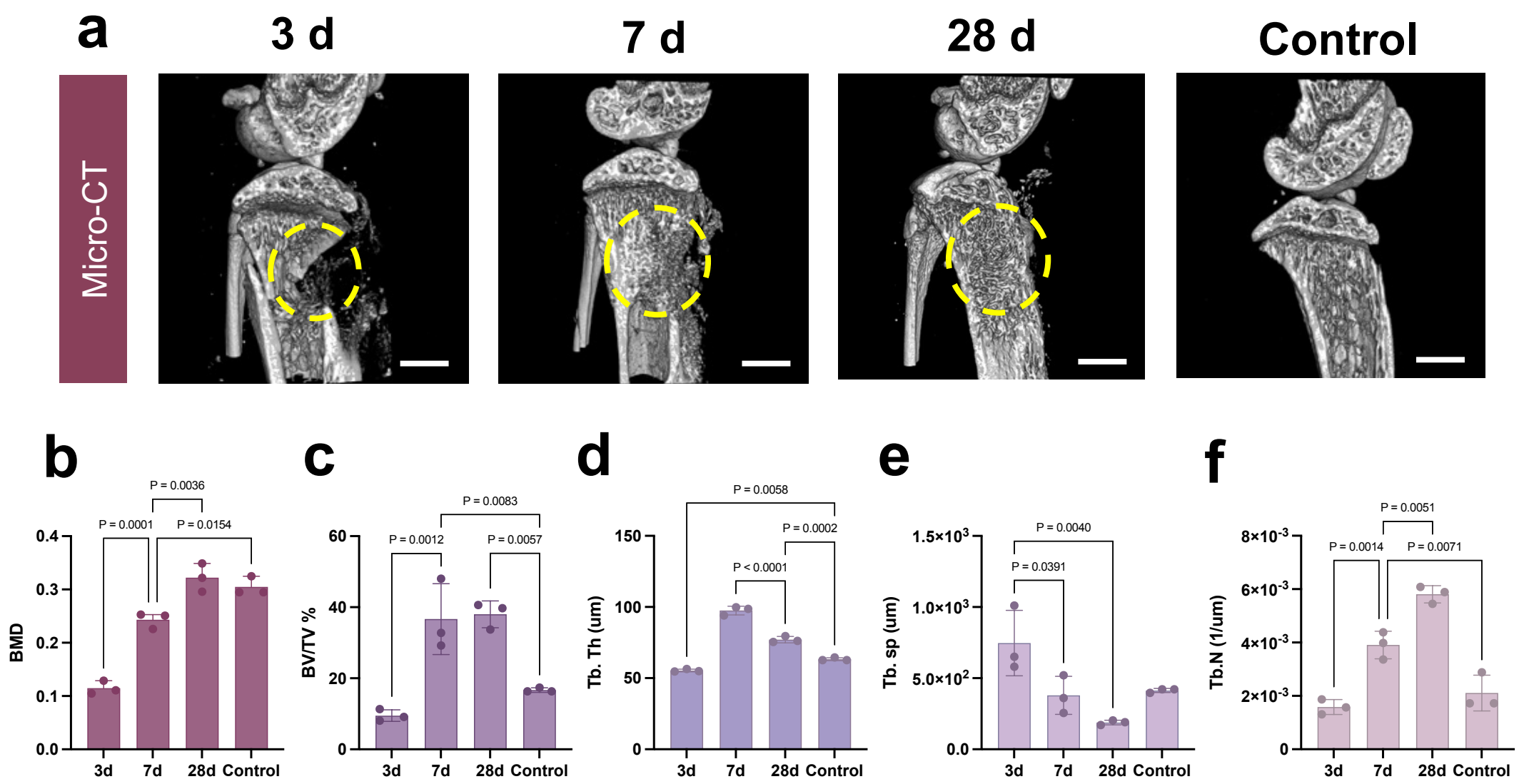

Supplementary Fig. 14. Micro-CT analysis of mouse tibial defect model. (a) Micro-CT images at 3 d, 7 d, 28 d post-surgery. **(b-f)** BMD (b), BV/TV% (c), Tb.Th (d), Tb. Sp (e), Tb.N (f) at 3 d, 7 d, 28 d post-surgery. BMD:Bone Mineral Density, BV/TV%: Bone Volume Fraction, Tb.Th: Trabecular Thickness, Tb. Sp: Trabecular Separation, Tb.N: Trabecular Number. Scale bar: 0.2 cm. Three independent animals were included (n=3). P values were calculated by one-way ANOVA with Tukey's test for multiple comparisons.

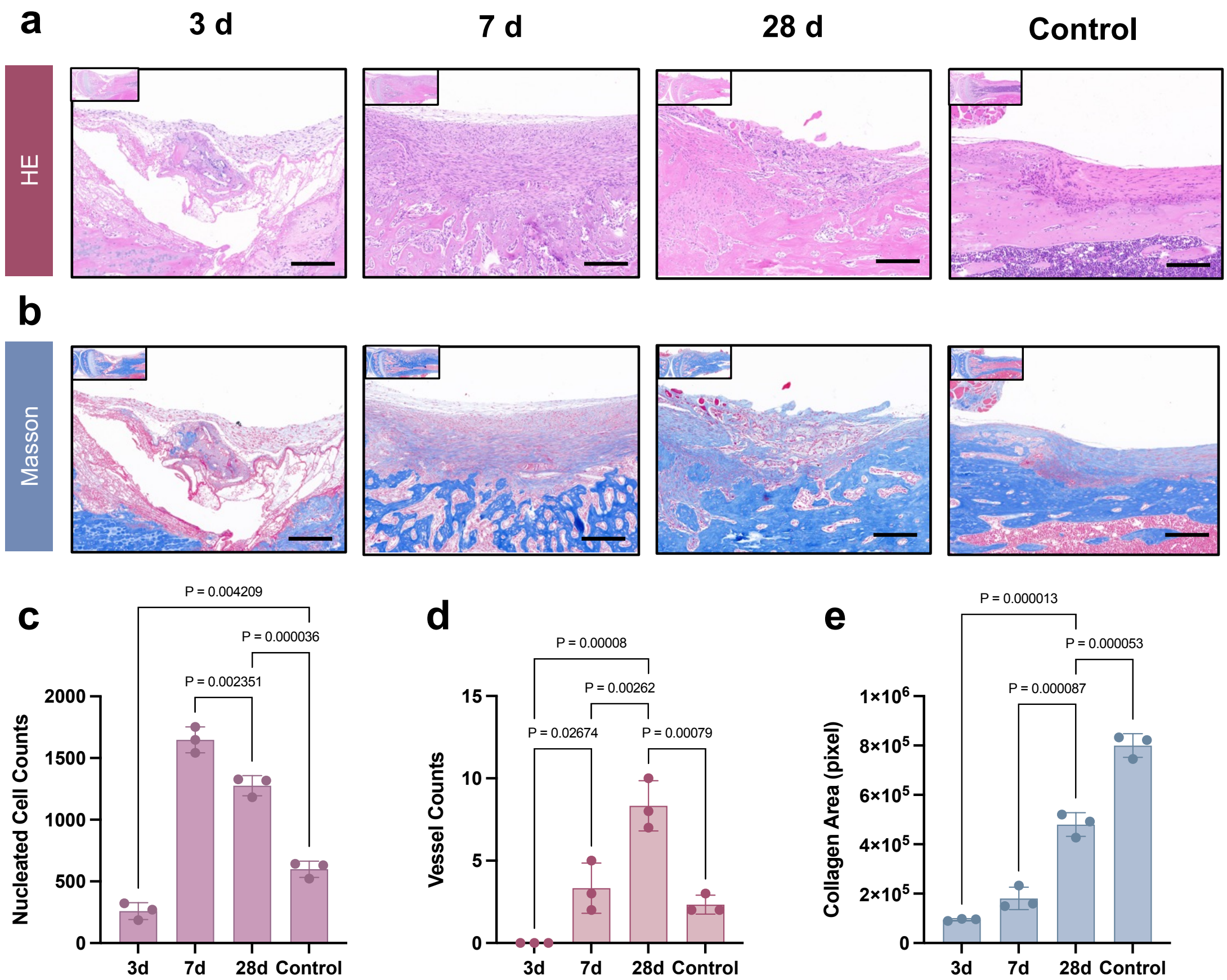

Supplementary Fig. 15. *Ex vivo* histological analysis of mouse tibial defect model. (a-b) HE (a) and Masson (b) staining of slices at 3 d, 7 d, 28 d post-surgery. **(c-e)** Nucleated cell counts (c), vessel counts (d), and collagen area (e) in the tibial defect area. Scale bar: 200 µm. Three independent animals were included (n=3). P values were calculated by repeated measures ANOVA with Tukey's test for multiple comparisons.

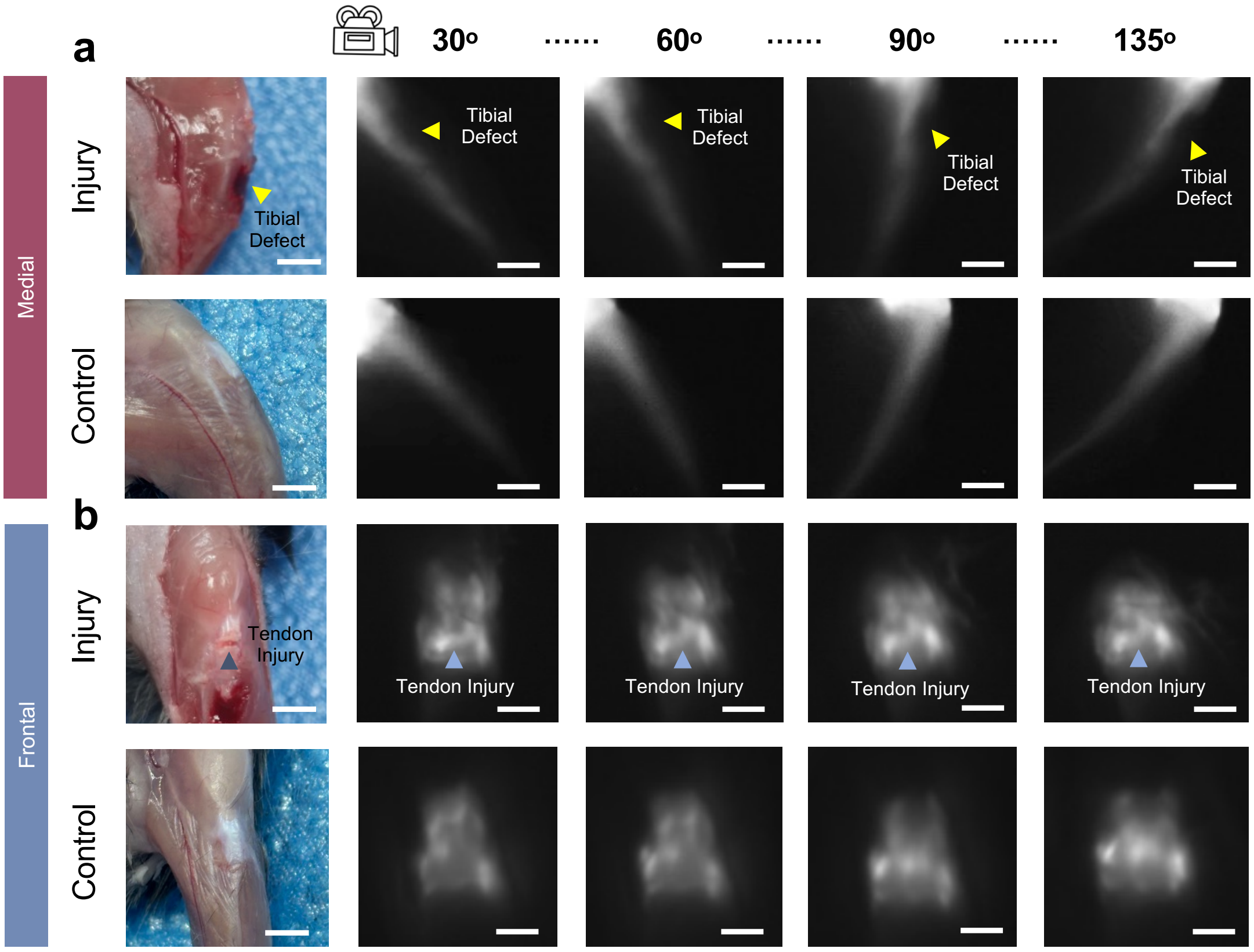

Supplementary Fig. 16. Real-time SWIR joint and bone imaging during entire knee flexion. (a-b) *In vivo* Bright-field photographs, and SWIR images at the timepoint 24.4 h of sequential imaging with different knee flexion angle from medial (a) and frontal (b) view. Scale bar: 0.5 cm.

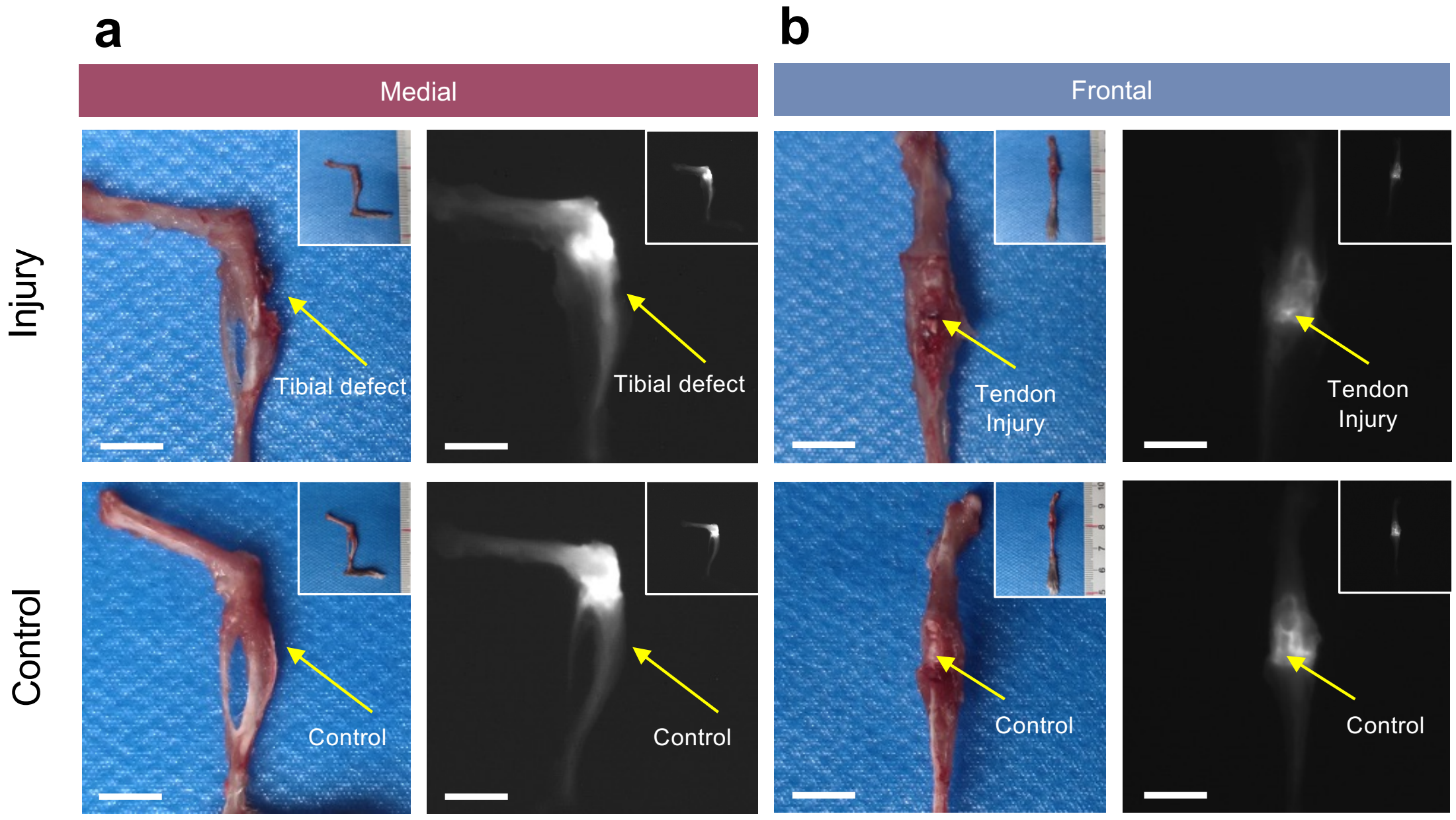

Supplementary Fig. 17. *Ex vivo* SWIR images for confirmation after *in vivo* joint and bone sequential imaging. (a) *Ex vivo* bright-field photographs, and SWIR images after dissecting the muscles at the timepoint 24.4 h of sequential imaging from medial view, showing the tibial defect area in the injury model. **(b)** *Ex vivo* bright-field photographs, and SWIR images after dissecting the muscles at the timepoint 24.4 h of sequential imaging from frontal view, showing the tendon injury site in the injury model. Scale bar: 0.5 cm.

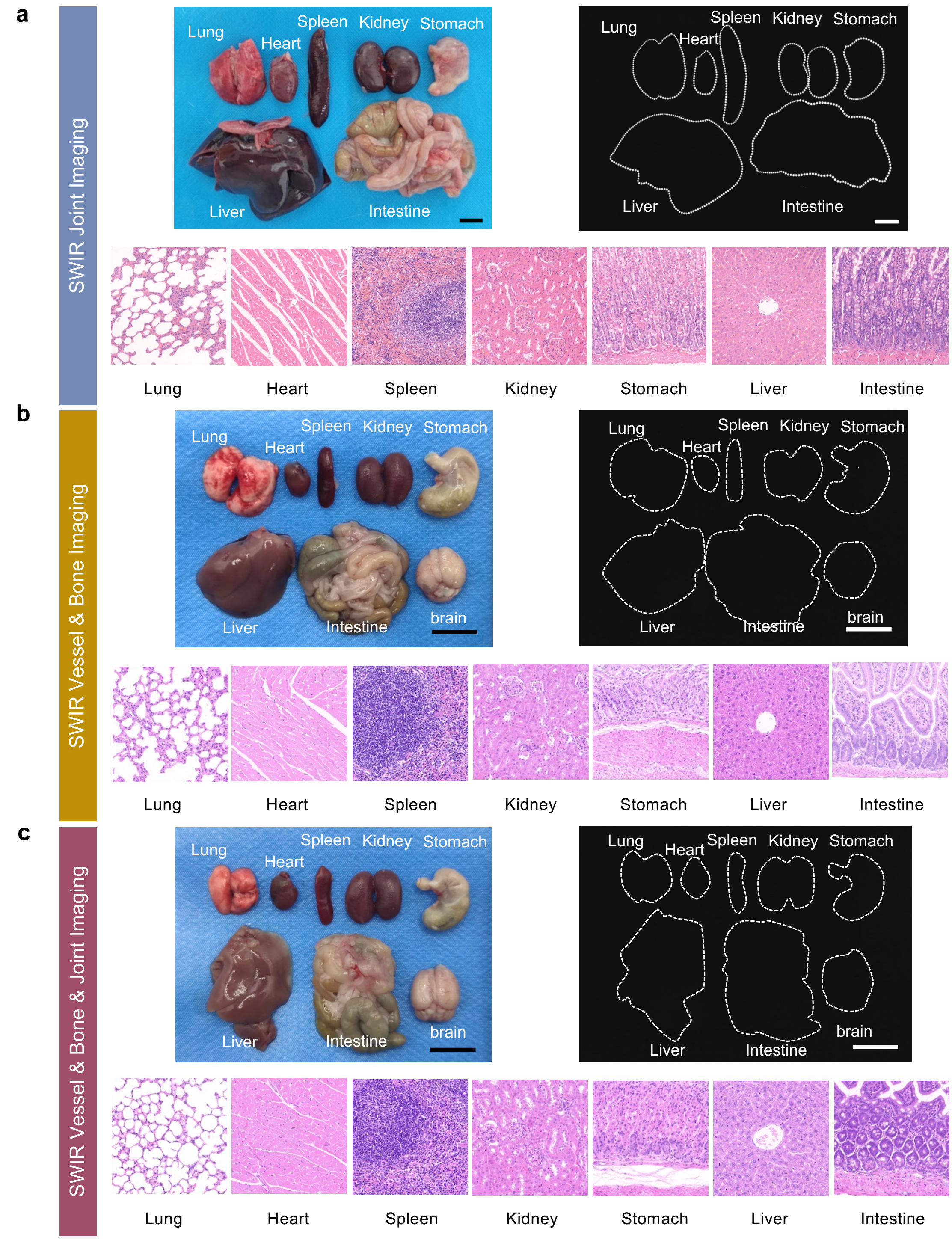

Supplementary Fig. 18. Biosafety investigation of SWIR fluorophores. (a-c) Bright-field image, SWIR image of and micrographs of HE-stained organs (Magnification: × 200) 7 d after SWIR joint imaging (a), SWIR vessel and bone imaging (b), and SWIR vessel, bone, joint imaging. Scale bar: 1 cm.

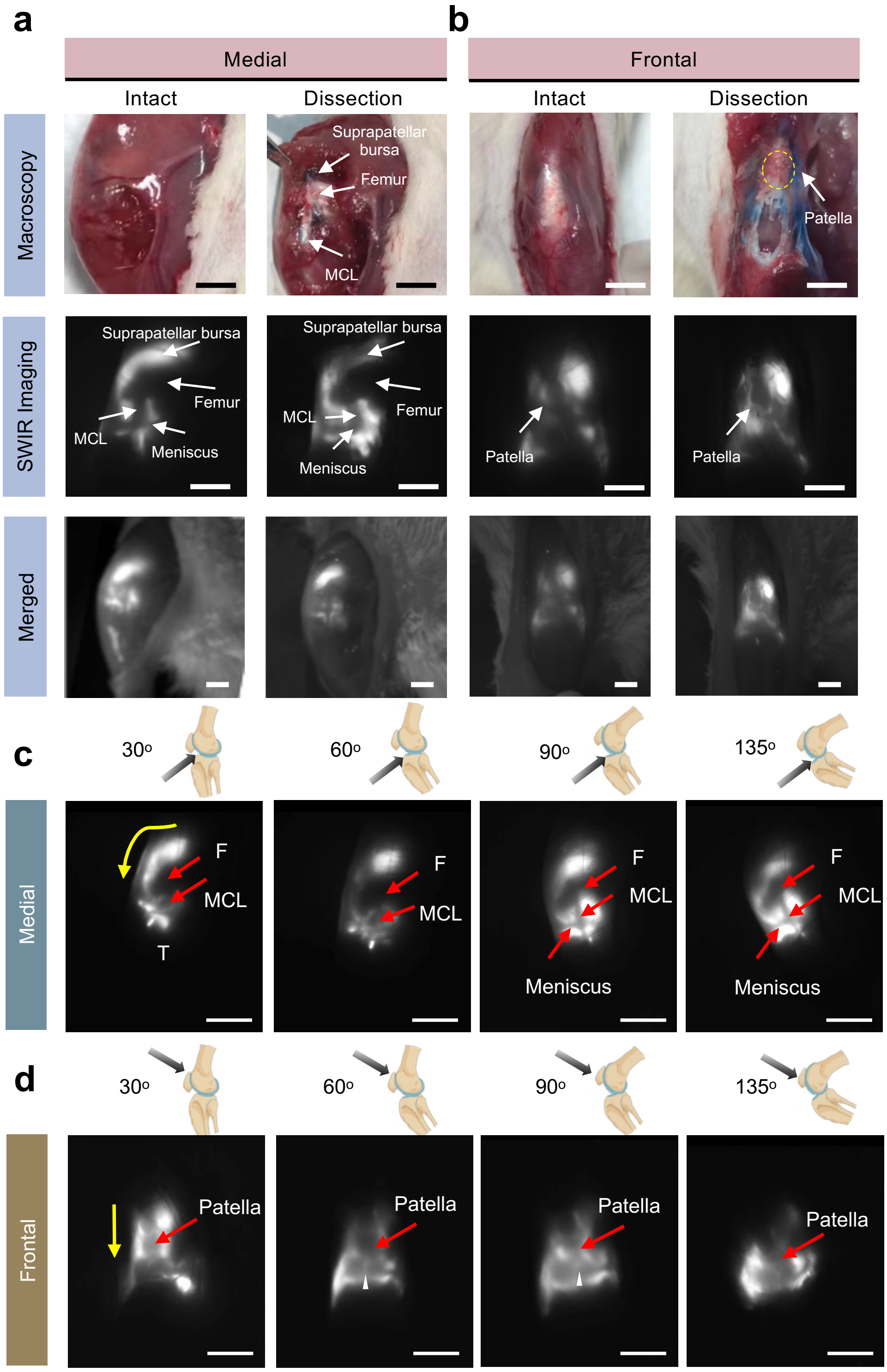

Supplementary Fig. 19. Images of SWIR joint imaging. a-b, Bright-field (Injection: methylene blue), original SWIR (Injection: SWIR fluorophores), and merged images of rat knee from the medial (a), and frontal view (b). Scale bar: 0.5 cm. **c,** Original SWIR images of knee anatomic structures with varying flexion angles from medial view. Scale bar: 1 cm. **d,** Original SWIR images of knee anatomic structures with varying flexion angles from frontal view. Scale bar: 1 cm.

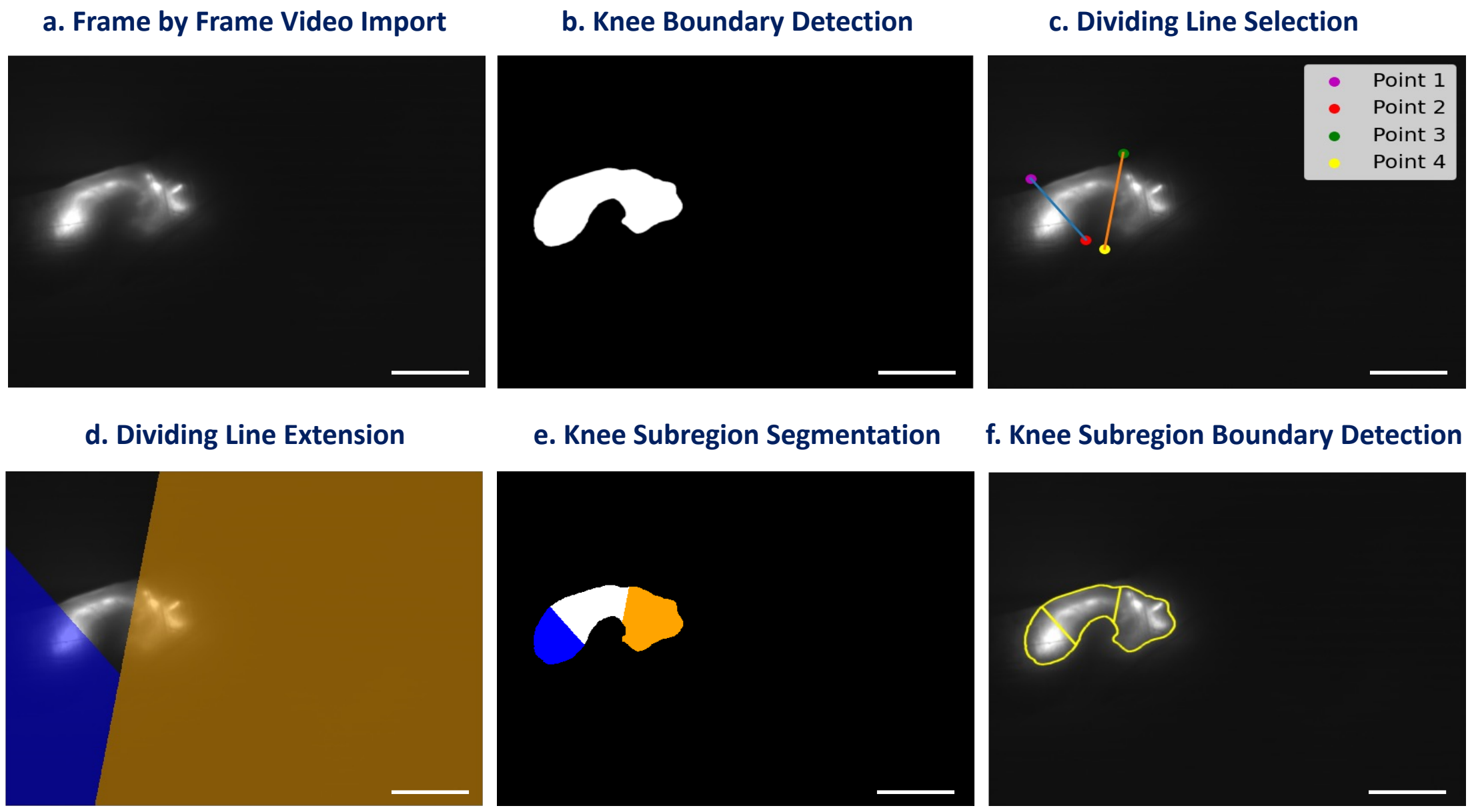

Supplementary Fig. 20. Data processing procedure for SWIR joint imaging. (a) Frame by frame video import. **(b)** Knee boundary detection. **(c)** Dividing line selection. **(d)** Dividing line extension. **(e)** Knee subregion segmentation. **(f)** Knee subregion boundary detection. Scale bar: 1 cm.
