# Supplementary material for: Next-generation functional diagnostic paradigm for musculoskeletal diseases based on live SWIR imaging
Source: Nat Commun. 2026 Sep 14;17:11269. doi: 10.1038/s41467-026-77137-4 (PMC13720147; doi:10.1038/s41467-026-77137-4)
Supplement: Supplementary file 4 [file 41467_2026_77137_MOESM2_ESM.pdf]

<u>Description of Additional Supplementary Files</u>

Supplementary Movie 1 Real time SWIR imaging of normal rat knee from the medial view.

Supplementary Movie 2 Real time SWIR imaging of normal rat knee from the frontal view.

Supplementary Movie 3 Real time SWIR imaging of rat tendon injury model from the frontal view.

Supplementary Movie 4 Real time SWIR imaging of rat multiple injuries model from the frontal view.

Supplementary Movie 5 Real time SWIR imaging of rabbit knee.

Supplementary Movie 6 Real time SWIR imaging of normal and locking human knee.

Supplementary Movie 7 Real time SWIR imaging of sequential bone and joint imaging.

Supplementary Movie 8 Demonstration of segmentation results for representative whole cycle of knee flexion and extension.
